# Transformer-Decoder GPT Models for Generating Virtual Screening Libraries of HMG-Coenzyme A Reductase Inhibitors: Effects of Temperature, Prompt Length, and Transfer-Learning Strategies

**DOI:** 10.1021/acs.jcim.4c01309

**Published:** 2024-11-07

**Authors:** Mauricio Cafiero

**Affiliations:** School of Chemistry, Food and Pharmacy, University of Reading, Reading RG6 6AD, U.K.

## Abstract

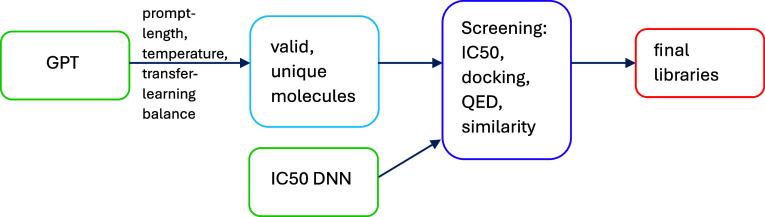

Attention-based decoder models were used to generate libraries of novel inhibitors for the HMG-Coenzyme A reductase (HMGCR) enzyme. These deep neural network models were pretrained on previously synthesized drug-like molecules from the ZINC15 database to learn the syntax of SMILES strings and then fine-tuned with a set of ∼1000 molecules that inhibit HMGCR. The number of layers used for pretraining and fine-tuning was varied to find the optimal balance for robust library generation. Virtual screening libraries were also generated with different temperatures and numbers of input tokens (prompt length) to find the most desirable molecular properties. The resulting libraries were screened against several criteria, including IC50 values predicted by a dense neural network (DNN) trained on experimental HMGCR IC50 values, docking scores from AutoDock Vina (via Dockstring), a calculated quantitative estimate of druglikeness, and Tanimoto similarity to known HMGCR inhibitors. It was found that 50/50 or 25/75% pretrained/fine-tuned models with a nonzero temperature and shorter prompt lengths produced the most robust libraries, and the DNN-predicted IC50 values had good correlation with docking scores and statin similarity. 42% of generated molecules were classified as statin-like by k-means clustering, with the rosuvastatin-like group having the lowest IC50 values and lowest docking scores.

## 1 Introduction

Inhibition of the HMG-Coenzyme A reductase (HMGCR) enzyme is the primary target for reduction of blood cholesterol.^1^ Though there are many popular options for these drugs (a major class of which is called *statins*), research into new inhibitors continues.^2^ One modern approach for the creation of novel drugs such as statins is the generation of virtual screening libraries that can be evaluated in silico quickly to arrive at promising candidates for synthesis and further testing. In this work, several variations on a generative, pretrained (GPT), attention-based decoder model are tested for their ability to generate robust virtual screening libraries for use in the process of drug design. Attention-based decoders^3^ or transformer-decoders, are most well-known for being the machine learning (ML) model behind OpenAI’s products, such as ChatGPT.^4^ Bagal et al. developed a transformer-decoder model for generating libraries of molecules, MolGPT,^5^ though it is unclear if there is any pretraining in that model. In that work, the authors train the transformer-decoder to replicate a molecule starting from a SMILES string and a set of properties. The trained model is then used as a generative tool to create libraries of molecules with specific properties. Thus, in order to create a library for a specific purpose, such as inhibition of HMGCR, one would request a library with molecules that have the properties of statin drugs, such as log *P*, etc. Yang et al., on the other hand, use a transformer-based *encoder* with transfer learning to develop a generative model and apply it to the generation of BRAF inhibitors.^6^ The authors then screened the molecules with docking calculations and synthetic accessibility scores (SAS) to support the libraries generated with their method. Tysinger et al. used a transformer-based *encoder*–*decoder* to design a model for hit expansion, or finding variations on a given scaffold.^7^ Their model was found to be highly generalizable to a range of targets.

Previous efforts to develop generative models for screening libraries largely used recurrent neural networks. Urbina *et al*.^100^ recently developed a RNN-based model that also incorporated retrosynthetic analysis and fragment analysis to create libraries of lead molecules. Another type of model that can be used for library generation is a variation on the convolutional neural network, the PixelCNN. Noguchi and Inoue recently developed a PixelCNN-based model that can build libraries from a target fragment, allowing for more directed searching of chemical space.^8^

The current work, which uses a transformer-decoder, explores the effects of temperature and prompt length on molecular generation. Temperature refers to how each successive atom in a molecular structure is chosen. At temperature zero, the most probable next atom is chosen based on what the model has learned. At higher temperatures (which for these models often run from zero to two or so), the most probable next atom may be chosen, but there is also a chance that a *less probable* next atom is chosen. While this may affect that percentage of valid molecular structures generated by a model, it increases the chemical space that is explored. Prompt length refers to how the model begins to build a molecular structure. Generation may be started with a single atom or may be stated with a full scaffold. In this work, the generation is started with the first *n* atoms (for various values of n) from a randomly chosen molecule (a *seed* molecule), and the generated molecule is then rejected if it simply recreates the seed, ensuring that novel molecules are generated. This work is unique in the area of ML-based molecule generation in that it simultaneously explores three variables in the ML process: transfer-learning strategy, prompt length, and temperature, and the resulting data set shows how different combinations of these variables result in higher and lower quality libraries of generated molecules.

In order to evaluate the generated libraries, various virtual screening techniques must be used. In the current work, a deep neural network (DNN) is trained to predict IC50 values of molecules in the HMGCR enzyme. Samizo and Kaneko have used a variety of ML techniques to screen molecules that are effective inhibitors of HMGCR.^9^ For example, they used various linear regression methods, support vector regression methods, decision trees, random forests, and gradient-boosting methods with RDKit and Mordred descriptors to predict IC50 values for HMGCR inhibitors. Khoa et al. compared classical scoring functionals for binding affinity of ligands to HMGCR to ML-based scoring functions and found that the classical functions outperformed the ML functions.^10^ The ML models used in their work not only included random forest and gradient-boosting methods like the work of Samizo and Kaneko but also included neural network and DNN-based methods. This work also screens libraries by searching for fragments and evaluating the similarity to known statins. Moorthy et al. performed QSAR modeling of the inhibitory power of ligands for HMGCR and found that the polar functional groups and fragments were crucial for the inhibitory activity of the molecules.^11^ In this work, the generated libraries are screened for the presence of various polar (and nonpolar) moieties, which are present in known statins.

## 2 Methods

### 2.1 Dense Neural Network for Predicting IC50 Values

A data set of all compiled HMGCR inhibitors was downloaded from BindingDB.org.^12^ Data were filtered to remove duplicates, outliers, and null values, and to include only those entries with a specific IC50 value, i.e., values with > or < were excluded. This resulted in a data set of 905 inhibitor SMILES strings and corresponding IC50 values in units of nanomolar (nM). This data set will be referred to as the BDB905 data set in the rest of this work. The molecule SMILES strings were featurized with Mordred descriptors^13^ as implemented in Deepchem.^14^ Morgan Fingerprints and RDKit descriptors^15^ were also tested but not used in production. The Mordred descriptors were reduced from 1613 features to 75 features using principle component analysis as implemented in Scikit Learn.^16^ IC50 values (in nM) ranged from 0.16 nM to over 10^9^ nM, and so they were transformed with the natural log function for the fitting process (called ln-IC50 here), leading to a much smaller range (−2 to about 13) that could be fit more easily.

**Figure 1:**
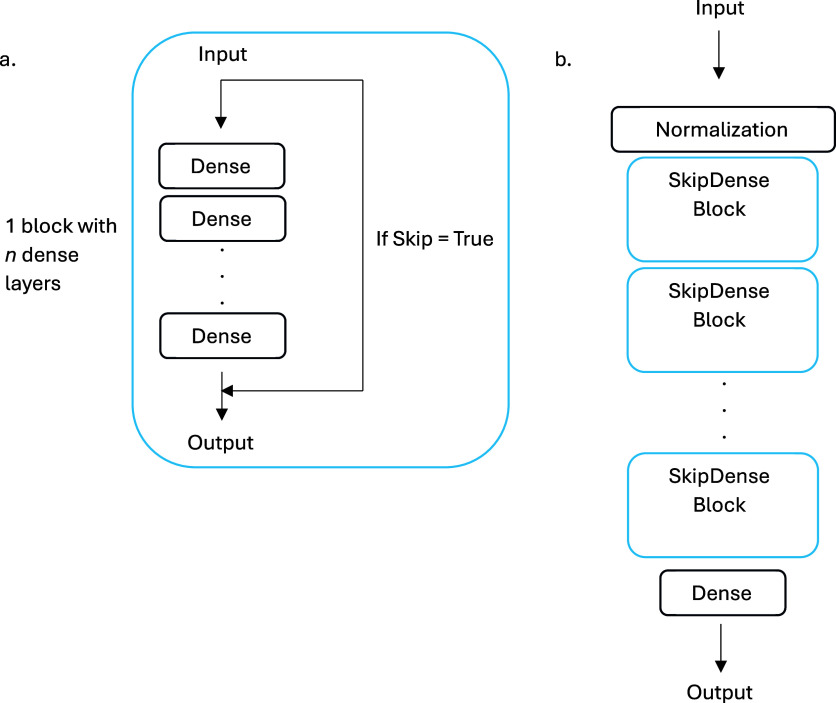
(a) SkipDense block and (b) DNN featuring SkipDense blocks.

The BDB905 data set was fit using a modified form of the DenseNet architecture^17^ wherein the network is made of blocks (referred to here as SkipDense blocks), each containing *n* dense layers and an optional skip connection from the block input to the block output, essentially allowing the block input to skip that block entirely while a second copy of the input progresses through the block as normal (Figure 1). DenseNet has been shown to have excellent performance for nonlinear fitting.^17^ The models used here had a normalization layer (for RDKit descriptors only; for Mordred descriptors, normalization was performed prior to PCA reduction), one, two, or three SkipDense blocks, and a dense output layer. The models were trained with the Adam optimizer and used a learning rate of 0.002, an l2 regularization constant of 0.01, 400 nodes per layer, and 4 dense layers per block. LeakyReLU activation was used on all dense layers except the output, which used linear activation.

The BDB905 data set was split into training and validation sets (90/10 split), and models were trained for 150 epochs. The mean absolute error was used as the loss function for optimization, and the training and validation scores were calculated for each model trained. Eight models were trained and evaluated: 1 SkipDense block with a skip, 1 SkipDense block with no skip, 2 SkipDense blocks with skips in both, 2 SkipDense blocks with no skips, 2 SkipDense blocks with no skip in the first and a skip in the second, 2 SkipDense blocks with a skip in the first and no skip in the second, 3 SkipDense blocks with no skip in the first and skips in the second and third, and 3 SkipDense blocks with no skip in the first and third blocks and a skip in the second block. Since the models had similar losses and scores, a set of four statin molecules was used as a “fine-tuning” of sorts to select the final model for production. These four statins, cerivastatin, simvastatin, atorvastatin, and rosuvastatin (which are part of the training set), have known IC50s of 3.54, 2.74, 1.16, and 0.16 nM, respectively.^18^ The predicted IC50 scores for these four molecules were extracted from each trained model so that their accuracy and relative ordering could be used as a final criterion to select the best model. These four relative IC50 values were used as the four compounds have well-established relative efficacies that have been verified clinically, and so the model’s ordering of these can provide a “final check” of the model’s accuracy. With training/validation scores of 0.90+/0.80+ (see Table 1), it is believed that the accuracy of predicted IC50 values for any of these models will be high, regardless of ordering.

**Table 1:** Loss (MAE, nM) and *R*^2^ Scores for Training and Validation Sets of the BDB905 Data Set with Various DNN Models^a^

model	training loss	validation loss	training score	validation score	known MAE
1 block, skip = false	0.8693	1.3471	0.92	0.85	5.83
1 block, skip = true	0.8616	1.4267	0.92	0.82	4.95
2 blocks, skip = true, true	0.8040	1.4208	0.92	0.82	4.60
2 blocks, skip = false, false	0.8996	1.3859	0.92	0.86	3.72
2 blocks, skip = false, true	0.8834	1.3713	0.89	0.84	3.52
2 blocks, skip = true, false	0.8540	1.3669	0.92	0.84	3.35
3 blocks, skip = false, true, true	0.9060	1.3920	0.91	0.84	6.58
3 blocks, skip = false, true, false	0.8770	1.4157	0.92	0.85	5.69
2 blocks, skip = false, true*	0.8100	1.3200	0.92	0.84	4.19

a The final column is the MAE in nM against four experimental statin values: cerivastatin, simvastatin, atorvastatin, and rosuvastatin. *indicates a retrained model.

### 2.2 Pretrained Attention-Based Decoders for Drug-like Molecules

A data set of 40,000 molecular SMILES strings was downloaded from the in vitro data set at ZINC15,^19^ which consists of substances that are reported or inferred active at 10 μM or less in binding assays. The in vitro data set is the second-to-lowest classification of biogenic molecules in ZINC15, meaning that while every compound in the data set has a measured or inferred bioactivity via *some* binding assay (these can vary greatly in the data set), they have *not necessarily* been tested in vivo, and they may or may not be FDA-approved or world drugs. This also means that the data set does contain *some* FDA-approved drugs and does contain *some* drugs that have been tested in vivo. Overall, this data set will be considered *drug-like* and will be referred to as the ZN1540K data set in the rest of this work. SMILES strings were tokenized using the SmilesTokenizer from DeepChem^14^ and the vocabulary file provided at their GitHub page.^20^ Tokenized SMILES strings were then padded with padding tokens ([PAD]) to the length of the longest SMILES string in the data set. Inputs for each tokenized SMILES string were created as strings with a length of one less than the longest SMILES string in the data set, missing the final token. Ground truth for each SMILES string was the same as the input string but shifted by one, i.e., missing the first token and including the last token.

**Figure 2:**
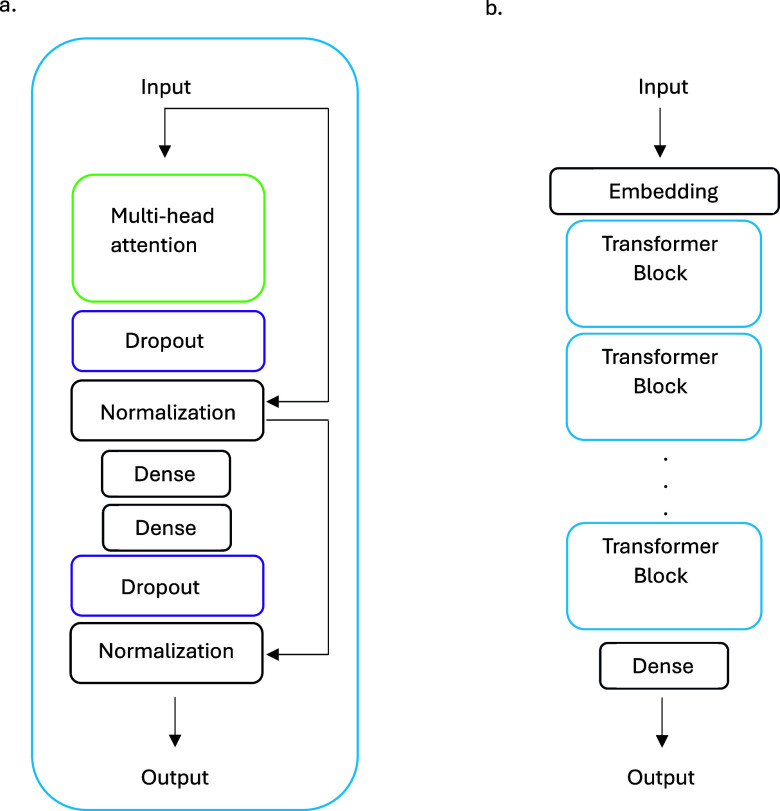
(a) Transformer block with a multihead attention layer and (b) GPT model used in this work.

The ZN1540K data set was then used to pretrain four attention-based decoder models, which we will refer to as Generative pretrained (GPT) models. Each GPT consisted of an input layer, one to four transformer blocks, and a dense output layer (Figure 2). The general structure of this model was adapted from a text-based decoder.^21^ The transformer block used 256 nodes in the dense layers, ReLU activation in the first dense layer, and a dropout rate of 10%. Attention layers in the transformer blocks all used 4 attention heads and a key dimension of 256. The embedding layer had a dimension of 256, and the output dense layer had a dimension of 85 [the size of the vocabulary for the combined ZN1540K and ChEMBL1081 (see below) data sets] and used SoftMax activation to generate token probabilities. The training of the GPT models used the Nadam optimizer and sparse categorical cross-entropy for the loss function.

### 2.3 Transfer Learning for HMGCR Inhibitor Generating Models

A data set of all HMGCR inhibitors was downloaded from ChEMBL.^22^ As this data set was used to train the GPT models on SMILES strings, all entries could be used, regardless of whether they had a valid IC50 value and accounted for 1081 unique SMILES strings. We will refer to this data set as the ChEMBL1081 data set for the rest of this work. This data set is distinct from the BDB905 data set used above, despite having some overlap. In order to estimate this overlap, the SMILES for both data sets were converted to canonical SMILES using RDKit and compared against each other. While canonical SMILES can still differ between two algorithms, in this case all were generated by the same algorithm. Only 124 canonical SMILES strings were found to be common between the two data sets. To further emphasize the difference between the two, the BDB905 data set had 905 entries with a well-defined IC50 value, that is, 905 values with an implied “=” after all “<” and “>” values were removed. The ChEMBL1081 data set only had 232 entries with an “=” for the IC50 value, with 868 entries having “NaN” (the rest did not have IC50 values). When the IC50 value distributions for both data sets are examined, the values in the ChEMBL1082 data set had ∼190 values less than 100 nM, while the BDB905 data set had over 600 values less than 100 nM. The BDB905 data set also had values up to and past 100,000 nM, meaning that it had ∼two-thirds active and ∼one-third relatively inactive examples for the DNN to learn from, which is crucial. The ChEMBL1082 data set had values only up to 90,000 nM. The two separate data sets were used to provide more data diversity to the process.

The ChEMBL1081 data set was used to generate four models. In Section 2.2, it was specified that four GPT models were trained with one, two, three, and four transformer blocks. Additional transformer blocks were added to each of the initial GPT models so that they all had a total of four blocks. The added blocks were then trained on the ChEMBL1081 data set, while the weights for the pretrained blocks were frozen. For example: the GPT with 2 transformer blocks had the weights for those two blocks frozen. Two more blocks were added, and the new 4-block model was trained with only the weights for the two new blocks and the output dense layer trainable. All layer details were the same as in Section 2.2 and this training also used the Nadam optimizer and sparse categorical cross-entropy for the loss function. Pretrained blocks were frozen to allow only the newly added blocks to learn from the ChEMBL1081 data set.

The first of the resulting four models was referred to as NoX (no blocks transferred, all four blocks pretrained, none trained on the ChEMBL1081 data set). This model serves as a control, as it was trained on 40,000 drug-like molecules, but had no specific HMGCR training. The next three models were referred to as the 1X, 2X, and 3X models, having one, two, or three pretrained blocks transferred, and three, two, and one blocks fine-tuned on the ChEΜBL1081 data set. Finally, a fifth model with four transformer blocks was trained only on the ChEMBL1081 data set, with no transferred blocks. This was referred to as the SO models (statin-only). All five models were trained for an additional 50 epochs with all weights on all blocks trainable, allowing cooperativity between the blocks, which had before been divided into “drug-like” blocks and “statin-only” blocks. These fully optimized models were referred to as the NoXALL, 1XALL, 2XALL, and 3XALL models.

A subset of the ChEMBL1081 data set was created containing only those molecules with known IC50 values. This yielded a set of 232 molecules with experimental IC50 values (referred to as the ChEMBL232 data set in the rest of this work) and was used as a control/comparison for the DNN-predicted IC50 values. This comparison may be found in Table 2.

**Table 2:** Average Properties of the Docking Subset of the Training Data Sets^a^

	IC50 (nM)	QED	score (kcal/mol)	% similarpairs	% fluoro-phenyl	% decalin	% similar simvastatin	% similar atorvastatin
BDB905	93	0.46	–7.95	54.84	72.92	9.62	18.76	65.74
ChEMBL1081	60	0.39	–7.90	29.72	57.26	9.13	10.37	53.11

a IC50 is given as calculated by the DNN. For comparison, the BDB905 average experimental IC50 is 91 nM, and the ChEMBL1081 average experimental IC50 is 40 nM (only 232 compounds in this library had IC50 reported). % of molecules in the training data sets with given fragments and % of molecules with Tanimoto similarity to known statins of 0.25 or greater.

### 2.4 Generation of Molecule Libraries

All models were used to generate molecule libraries for the virtual screening. In the proof-of-concept models, molecules were generated by feeding each model a “seed” or prompt of 12 input tokens and asking it to predict the most likely next token in the sequence (called temperature = 0 molecule generation). This was done 80 times per seed, so that the resulting molecules consisted of 92 tokens each. Each model was fed 1000 12-token prompts, with the hope of generating 1000 molecules in each library. The seeds were generated by taking a random sample of 1000 (or, later, 5000) molecules from the combined ZN1540K and ChEMBL1081 data sets, tokenizing them, and choosing the first 12 tokens in each. Once generated, each token sequence was transformed back to a SMILES string for further analysis.

The library generation process was repeated with a higher “temperature” of 0.5 using a multinomial-like sampling strategy. Higher temperature, in this case, means that each token selected to add to the seeds is not necessarily the most probable next token but possibly a token of lesser likelihood. When the model is queried for the next most likely token, it does not just give the single token but provides the probability for every token in the vocabulary (85 total tokens for this work). In a temperature of 0 molecule generation, the token with the highest probability is chosen, but if the temperature is not zero, some other token is chosen. Higher temperature generation was performed by taking the probability distribution that the next token, *t*, given by the model will be the *i*-th vocabulary word, *f*_*i*_(*t*), and transforming it according to
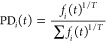
1and then using this PD(*t*) as the probability function for the Numpy random.choice tool. The token selected by this process was then added to the seed for a total of 92 tokens. The libraries generated with the five models from Section 2.3 are referred to as *1K12S* libraries, where 12 is the number of seed tokens or prompt length. Thus, for the five fully trained models, the resulting libraries are NoXA1K12S (*No X*fer-learning, *A*ll-layers trained, *1K* prompts, and *12 S*eeds tokens), 3XA1K12S (*3 X*fer-learning layers, *A*ll-layers trained, *1K* prompts, and *12 S*eeds tokens), etc.

After the proof-of-concept, 1000 prompt library was tested, three more libraries were created for each of the five models at two temperatures (0.0 and 0.5), leading to 30 total libraries. These libraries had 5000 prompts each and had prompt lengths of six, nine, or 12 tokens. These libraries are referred to as *5KnS* libraries, where n is the prompt length. Thus, for the five fully trained models in Section 2.3, the resulting libraries are NoXA5K12S (*No X*fer-learning, *A*ll-layers trained, 5K prompts, and 12 Seed tokens), 3XA5K12S (*3 X*fer-learning layers, *A*ll-layers trained, *5K* prompts, and *12 S*eed tokens), etc.

### 2.5 IC50 Prediction, Docking, Quantitative Estimate of Druglikeness, ADME Properties, Substructure Searching, and Tanimoto Similarity

Several strategies were used to screen the generated libraries. First, the DNN from Section 2.1 was used to predict an IC50 value for each molecule. The predicted IC50 values were used to separate the libraries into two sets: all molecules were included in a subset referred to here as “refined”, and if the predicted IC50 value for a molecule was less than 1000 nM, that molecule was also added to a “docking” subset. All molecules in the docking subset were then docked in the HMGCR binding site using the Dockstring^23^ package for Python. This package accepts a SMILES string as input and then prepares the molecule by protonating it at a pH of 7.4 using Open Babel,^24^ generating a conformation using ETKG from RDKit,^15^ optimizing the structure with MMFF94, and computing charges for all atoms using Open Babel, all while maintaining any stereochemistry in the original SMILES string. The prepared molecule is then docked into the protein binding site using AutoDock Vina^25^ with default values of exhaustiveness, binding modes, and energy range. The prepared HMGCR binding site from the DUD-E database^26^ was used for docking. Poses were visualized with PyMOL.

RDKit^15^ was used to calculate various ADME properties, including molecular weight, calculated log *P* (*A* Log *P*), hydrogen bond acceptors and donors (HBA and HBD), number of rotatable bonds, number of aromatic rings, polar surface area, and number of alerts for undesirable moieties. These properties were also used to calculate the quantitative estimate of druglikeness (QED),^27^ which uses a fit of ADME properties to predict how drug-like a molecule will be. As the QED value is a function of the ADME properties, only the QED is reported here, with the rest of the ADME properties available in the Supporting Information. RDKit was also used to search for several substructures from known statin drugs: the atorvastatin pharmacophore (3,5-dihydroxypentanoic acid, which binds to ASP 671, LYS 672, and LYS 673 in HMGCR), the HMG coenzyme-A pharmacophore, a fluorophenyl ring, a methanesulfonamide group (both found in type-2 statins), and a butyryl group and decalin ring (both found in type-1 statins). Absolute numbers and percentages of these substructure in the libraries are reported. The two most compelling substructures and similarities are reported here, and the rest are available in the Supporting Information.

Morgan fingerprints^28^ (radius of 2, so roughly equivalent to extended connectivity fingerprints of diameter 4) were used to find Tanimoto similarity^29,30^ for several sets of molecules. First, the average similarities of all of the molecules in each library were calculated by averaging the pairwise similarity between all unique sets of molecules; therefore, for a library of *n* molecules, there were *n*(*n* – 1)/2 unique similarity values. This was used as a measure of the amount of variation in each library. The average similarities were also calculated between all molecules in each library and a set of six statin molecules: atorvastatin, rosuvastatin, fluvastatin, simvastatin, lovastatin, and pravastatin. The first three in this set are well-known type-2 statins, and the last three are well-known type-1 statins. This type of similarity to know actives is often used as a screening criteria.

**Figure 3:**
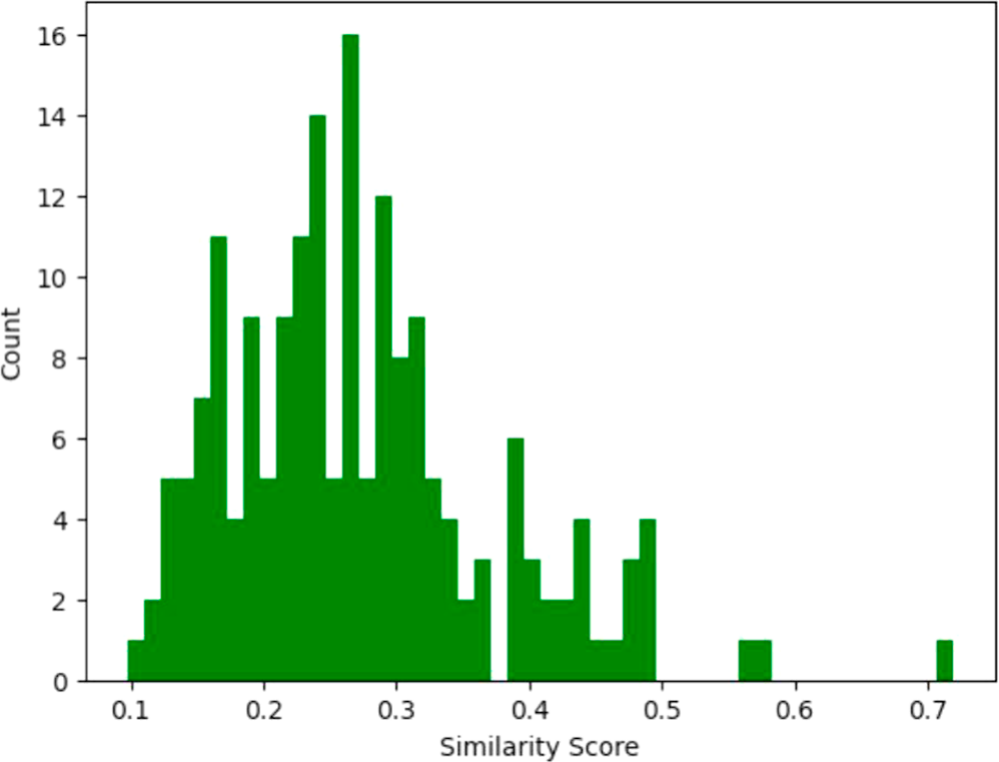
Distribution of average Morgan fingerprint-based Tanimoto similarities of the BDB905 data set, taken from a list of the molecules sorted by IC50 values and taken 5 at a time.

In order to use similarity as a screening criteria, a benchmark must be established, with the fingerprint and similarity method being used. In order to do this, the BDB905 data set, which contained 905 experimental IC50 values, was examined. The molecules in the data set were sorted by IC50 values from lowest to highest, and the average similarity for each set of 5 consecutive molecules was calculated, i.e., the average similarity was found for molecules 1–5, 6–10, 11–15, etc., with the rationale that if the molecules have similar activity, then their similarity may correlate with that.^30^Figure 3 shows the distribution of similarities for this data set. The range that occurred most was ∼0.25, meaning that more sets of 5 molecules with similar IC50 values have similarities of ∼0.25 than any other value. It is worth noting that higher values most often correspond to lower IC50 groupings. For example, the highest similarity, 0.72, corresponded to the 11 to 15 grouping, which had an average IC50 value of 0.72 nM, and the second highest similarity, 0.57, corresponded to a grouping with an average IC50 of 1.22 nM. The lowest similarity, on the other hand, 0.12, corresponded to two groupings with average IC50 values of 7180 and 749,800 nM. Thus, in this work, 0.25 is used as the cutoff value for similarity: if the similarity is 0.25 and above, there is a chance of similar activity.

The final set of unique, submicromolar molecules was analyzed for ease of synthesis using the SAS, which breaks molecules down by fragments and uses fragment information from the ChEMBL database to estimate ease of synthesis.^31^ This method has been found to agree with expert analysis with an *R*^2^ value of 0.89.

## 3 Results and Discussion

**Figure 4:**
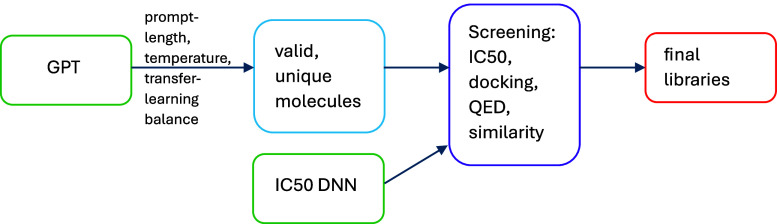
Workflow for generating libraries.

Figure 4 summarizes the procedure for generating libraries, refining them, and screening them. Each step is discussed below.

### 3.1 DNN for IC50 Results

Table 1 shows the training results for the DNN models with various configurations of the SkipDense blocks. Loss values are less informative, as they are all fairly close; therefore, *R*^2^ scores are a better indicator of the accuracy of the model. The validation sets lagged the training sets by about 0.08 (8%) on average, but the smallest gap between training and validation was for the 2-block model, with no skip in the first block and a skip in the second block (2BFT). The values here may be compared with the HMGCR predictive IC50 models of Samizo and Kaneko, who found a *R*^2^ of 0.728 for their test data using their best 2D model, a gradient boosting model, and *R*^2^ of 0.772 for their test data using their best 3D model, a Gaussian process regression model. The best validation data (called test data in their work) *R*^2^ value here, 0.86, is considerably more accurate than that in their work. The considerably simpler QSAR predictive HMGCR IC50 models of Moorthy et al. have *R*^2^ values for their test data of 0.78 to 0.83, closer to the values obtained in this work.^11^ A secondary criterion considered for model accuracy was how the models treated a subset of known statins: cerivastatin, simvastatin, atorvastatin, and rosuvastatin all have well-known IC50 values. The MAE for these molecules with each model is given in Table 1. The 2BFT model had the second smallest MAE for these test statins. The ordering that the models gave these predicted statins was also studied. The experimental values are in the order cerivastatin > simvastatin > atorvastatin > rosuvastatin. The “one block with skip” model was the only one to get the ordering correct, but it had a larger variance than other models. The 2BFT model was able to get three out of four correct in the ordering. For these reasons, the 2BFT model was retrained on the same data for the same number of epochs with different starting random conditions (initial weights, etc.). This reoptimization produced the lowest loss values of all the trials, raised the difference between training and validation scores slightly, and raised the known MAE slightly, but it also achieved correct ordering of the known statins. This model was used for all subsequent predictions of IC50 values. While all possible combinations of *skip* and *no skip* were tested for models with 1 and 2 SkipDense blocks. When testing models with 3 SkipDense blocks, no improvement was found in loss or *R*^2^ for the first two 3-block models tested, and the MAE for the known statins was larger than that for 2-block models, so no further testing of 3-block models was carried out.

### 3.2 Properties for Training Data Sets

The DNN and GPT training sets (ChEMBL1081 and BDB905) were analyzed for the same properties as the generated virtual screening libraries, DNN-predicted IC50, QED, and docking score, as well as for the presence of several moieties and similarity to known statins. Table 2 shows the average properties for docking subsets of the two training sets (those molecules with predicted IC50 less than 1 μm) as well as the average experimental IC50 for the BDN905 data set. More properties are available in the Supporting Information. The BDB905 docking subset had a low predicted IC50 value of 93 nM, comparable to the experimental average of 91 nM. Likewise, the ChEMBL1081 data set had a predicted value of 40 nM, compared to the experimental average of 60 nM. QED for both training sets was low, 0.46 and 0.39. Despite statins favoring low log *P* values, the average *A* log *P* values were both 4.00. Both had about 9 rotatable bonds, but while the BDB905 data set had about 55% similar pairs, the ChEMBL1081 data set only had 30% similar pairs. Both sets had similar average docking scores of −7.95 and −7.90. For reference, the docking scores for six known statins, executed with the same method, are −8.3 for atorvastatin, −8.5 for rosuvastatin, −8.6 for fluvastatin, −7.6 for simvastatin, −7.6 for lovastatin, and −7.1 for pravastatin. The average docking score for the three type-2 statins (first three) is −8.5, and the average for the three type-1 statins is −7.4. The overall average docking score for the six statins is −7.95, which is exactly what is reported here for these data sets.

**Figure 5:**
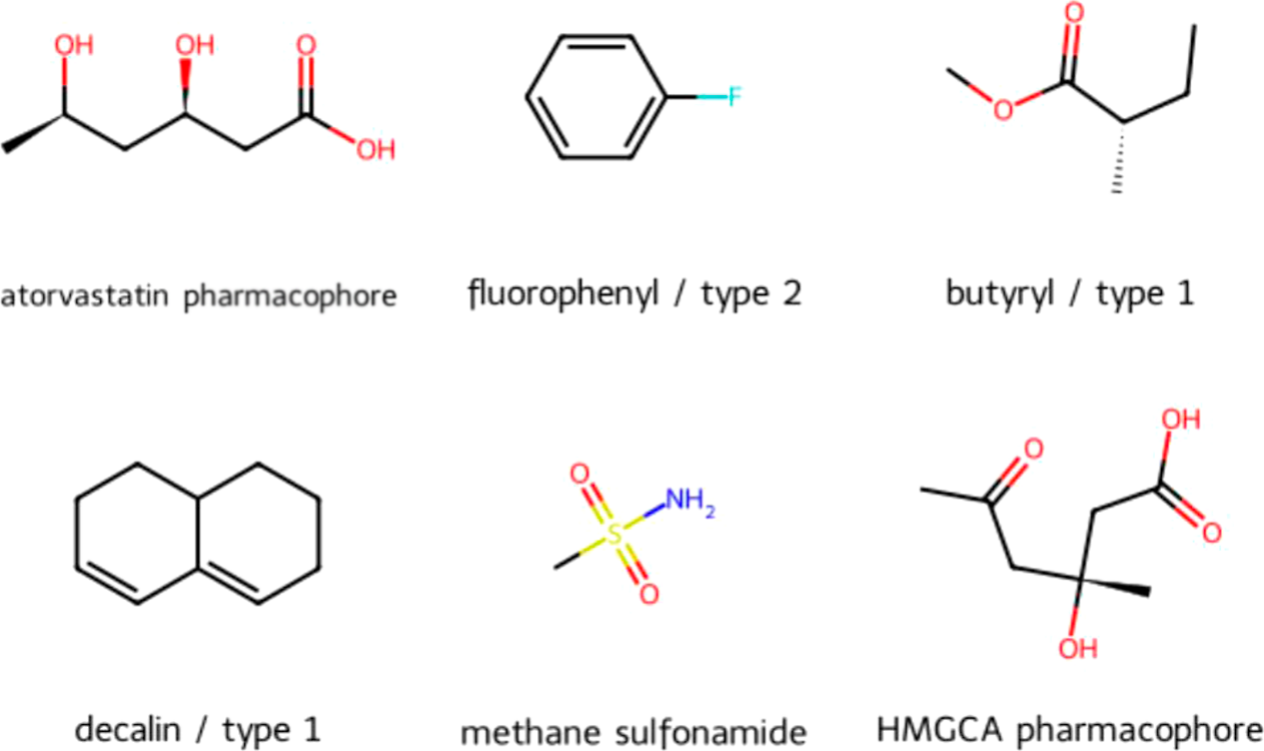
Fragments from known statins.

Figure 5 shows the six fragments searched for in the virtual screening libraries. Table 2 shows the % of molecules in the training data sets that contain each fragment (fragments not included here are in the Supporting Information). The type-2 statin fragments are present in much larger proportions (50–70%) compared to the type-1 fragments (10–15%), and so it will be expected that the libraries generated from these data sets will reflect this distribution. Finally, the percent of molecules with a greater than 0.25 Tanimoto similarity to known statins is also shown in Table 2 (statin similarities not included here are in the Supporting Information). Again, there is a much larger proportion of the type-2 statins (atorvastatin and rosuvastatin, 55–65%) compared to simvastatin, a type-1 statin (10–20%).

Pearson correlation coefficients were calculated for different combinations of properties of the two training data sets. Note that for these and all subsequent correlation analyses, the natural log of the IC50 was used rather than the IC50, in order to make the values more tractable. Pearson coefficients can vary from −1 to 1, indicating negative and positive correlation, and generally values from 0 to ±0.3 are considered weaker correlations, values from ±0.3 to ±0.5 are considered medium–strength correlations, and values from ±0.5 to ±1 are considered stronger correlations, though two-tailed *t* tests with 95% confidence (*p* = 0.05) were performed on the correlations to determine statistical significance. If the calculated *t*-score is greater than the value from the *t*-distribution table,^32^ then the correlation is significant, i.e., the null hypothesis that correlation is zero is disproved. Both data sets had weak/medium correlation between ln-IC50 and docking score (0.24 and 0.26 for BDB905 and ChEMBL1081, respectively) and slightly stronger correlation between docking score and number of aromatic rings (−0.39 and −0.32). *t* tests show that the ln-IC50/score correlations were statistically significant, as were the score/aromatic ring correlations. Other correlations were either very weak (docking score with QED) or inconsistent between the data sets, though several of them had statistically significant correlations. A comprehensive table of correlations and t-scores is included in the Supporting Information.

### 3.3 GPT Models for Producing Virtual Screening Libraries: Training

Four transformer (or attention-based decoder) models were pretrained on the 40,000 SMILES strings in the ZN1540K data set. They were trained to reproduce the input SMILES strings, or essentially, they were trained on the language of SMILES strings for drug-like molecules. The training results for these four models are shown in Table 3. The pretrained blocks were optimized for 75 epochs, except for the 2X pretrained blocks, whose loss stopped changing by 50 epochs. The trained weights for these models were then frozen for the next step of the process. Note that the NoX model, which had all 4 transformer blocks trained on the ZN1540K data set and had no fine-tuning applied, was optimized in two stages: 3 blocks were trained for 75 epochs, and then a fourth block was added and trained while the pretrained weights were frozen. Also note that the SO model had no pretraining. The other three models then had one, two, or three extra blocks added, and the new blocks were trained on the ChEMBL1081 data set while the pretrained blocks had frozen weights. The 1X model, which has 3 blocks trained on the ChEMBL1081 data set, needed the most epochs to reach loss-stability. As could be predicted, the 2X model, with two blocks being trained on the ChEMBL1081 data set needed fewer epochs, and the 3X model, with only 1 block being fine-tuned, needed the fewest epochs of training. The SO mode, which was training all four blocks on the ChEMBL1081 data set, needed only 50 epochs of training, owing to the fact that it had much less data to fit to four full blocks worth of weights (note that there are about 4.2 million parameters per block). The final models reached similar losses, except 3X, which was a bit higher. The 3X model was retrained to ensure this was not an error, and similar values were obtained the second time.

**Table 3:** Number of Epochs and Losses (Sparse Categorical Cross-Entropy) for the GPT Models Pretrained on the ZN1540K Data Set and the Fine-Tuned on the ChEMBL1081 Data Set

model	pretraining blocks	pretraining epochs	pretraining loss	blocks added	fine-tuning epochs	fine-tuning loss
NoX	3	75	0.0843	1	25	0.0792
1X	1	75	0.1311	3	150	0.0529
2X	2	50	0.0996	2	125	0.0696
3X	3	75	0.0843	1	100	0.1054
SO	0			4	50	0.0716

As proof-of-concept, the trained models were fed 1000 prompts with 12 seed tokens each to potentially generate 1000 molecule libraries. Those results are reported in the Supporting Information.

### 3.4 GPT Models with Varying Numbers of Seed Tokens and 5000 Prompts

Thirty new libraries were generated by feeding 5000 prompts with either six, nine, or 12 seed tokens to the five models. Tables 4 and 5 show the numbers of valid molecules generated (and percentages) for the *T* = 0.0 and *T* = 0.5 models. For a molecular generative model, a primary question is: can they generate a robust library of molecules? Table 4 shows the numbers of valid molecules generated by each fully trained model at *T* = 0.0, along with how many were duplicates and how many simply replicated the seed molecules. As with the 1000 prompt proof-of-concept model (see Supporting Information), the percentage of valid molecules generated (out of 5000 prompts) decreased from the NoX models to the SO models due to the models having fewer layers trained on the ZN1540K data set. In every case, though, the percentage of valid molecules increased when going from 12 to 9 to 6 seed tokens. This increase was modest for the NoX models (about 4%). The percent validity increases from 89 to 94% for the *T* = 0.0 libraries and from 84 to 88% for the *T* = 0.5 NoX libraries with 12 tokens, showing that the percentage of valid molecules can be increased with a smaller training data set by varying prompt length rather than using a larger training library. This increase was more dramatic for the SO models, going from 35 to 84% (*T* = 0.0) and from 32 to 76% (*T* = 0.5) valid molecules by changing prompt length. This increase in stable molecules generated can be attributed to the freedom the models have to generate molecules when starting with a shorter prompt: longer prompts can force a model to use a scaffold (or partial scaffold) that is not necessarily compatible with the learned patterns. A drawback, though, to the shortened prompts is that more duplicate molecules are generated. Tables 4 and 5 show that while about half of valid, generated molecules are duplicates for the 12 seed token models, up to 90% of valid, generated molecules are duplicates in the 6 seed token models at *T* = 0.0. For the 6 seed token, *T* = 0.5 models, though, the number is a slightly smaller ∼75%. Finally, when the seed molecules are removed from the libraries (the molecules from which the seed tokens were drawn), the number of molecules decreases more modestly, with ∼10% being seed molecules. The molecules with predicted IC50 values below 1 μm are then selected for the “docking” subset libraries, leaving only 1–3% of the original 5000 molecules for the *T* = 0.0 libraries and 2–7% for the *T* = 0.5 libraries, showing that overall the higher temperature models end up producing more desirable molecules. It should be noted that while the models with no fine-tuning (NoX) generate more molecules with submicromolar IC50 values, these sets have a higher average IC50 than the fine-tuned models (Table 8), meaning their values are clustered closer to 1 μM rather than 1 nM.

**Table 4:** Number of Molecules Generated by Each Model at *T* = 0.0 with Duplicates and Seeds Removed^a^

*T* = 0.0		molecules generated	%	after removing duplicates	%	after removing seeds	%	IC50 < 1000 nM	%
NoX	5K12S	4466	89.32	2288	45.76	1966	39.32	147	2.94
	5K9S	4558	91.16	1392	27.84	1237	24.74	80	1.6
	5K6S	4688	93.76	469	9.38	436	8.72	33	0.66
3X	5K12S	3586	71.72	1767	35.34	1584	31.68	148	2.96
	5K9S	3943	78.86	1122	22.44	1022	20.44	92	1.84
	5K6S	4375	87.5	989	19.78	891	17.82	36	0.72
2X	5K12S	3349	66.98	1624	32.48	1457	29.14	146	2.92
	5K9S	3634	72.68	1063	21.26	973	19.46	98	1.96
	5K6S	4589	91.78	401	8.02	365	7.3	43	0.86
1X	5K12S	2837	56.74	1267	25.34	1110	22.2	102	2.04
	5K9S	3316	66.32	867	17.34	781	15.62	69	1.38
	5K6S	4227	84.54	358	7.16	324	6.48	31	0.62
SO	5K12S	1763	35.26	690	13.8	545	10.9	62	1.24
	5K9S	2466	49.32	541	10.82	455	9.1	52	1.04
	5K6S	4213	84.26	261	5.22	231	4.62	21	0.42

a % is relative to 5000. Model designations are explained in Sections 2.3 and 2.4.

**Table 5:** Number of Molecules Generated by Each Model at *T* = 0.5 with Duplicates and Seeds Removed^a^

*T* = 0.5		molecules generated	%	after removing duplicates	%	after removing seeds	%	IC50 < 1000 nM	%
NoX	5K12S	4220	84.4	3827	76.54	3497	69.94	288	5.76
	5K9S	4306	86.12	3660	73.2	3447	68.94	284	5.68
	5K6S	4414	88.28	3445	68.9	3321	66.42	326	6.52
3X	5K12S	3538	70.76	2272	45.44	2079	41.58	197	3.94
	5K9S	3889	77.78	1733	34.66	1610	32.2	156	3.12
	5K6S	4375	87.5	989	19.78	891	17.82	111	2.22
2X	5K12S	3379	67.58	2066	41.32	2005	40.1	197	3.94
	5K9S	3686	73.72	1572	31.44	1458	29.16	155	3.1
	5K6S	4426	88.52	989	19.78	904	18.08	139	2.78
1X	5K12S	2738	54.76	1550	31.00	1384	27.68	142	2.84
	5K9S	3247	64.94	1359	27.18	1248	24.96	140	2.8
	5K6S	4170	83.4	912	18.24	826	16.52	133	2.66
SO	5K12S	1588	31.76	809	16.18	655	13.1	77	1.54
	5K9S	2373	47.46	790	15.8	690	13.8	92	1.84
	5K6S	3797	75.94	656	13.12	581	11.62	98	1.96

a % is relative to 5000. Model designations are explained in Sections 2.3 and 2.4.

**Table 6:** Number of Molecules with IC50 under 1 μM That Overlap between Different Libraries, *T* = 0.0^a^

*T* = 0.0, IC50 < 1000 nM		NoX			3X			2X			1X			SO		
		5K 12S	5K 9S	5K 6S	5K 12S	5K 9S	5K 6S	5K 12S	5K 9S	5K 6S	5K 12S	5K 9S	5K 6S	5K 12S	5K 9S	5K 6S
NoX	5K12S	147														
	5K9S	61	80													
	5K6S	24	26	33												
3X	5K12S	0	0	0	148											
	5K9S	0	0	0	43	92										
	5K6S	0	0	0	9	18	36									
2X	5K12S	0	0	0	29	13	5	146								
	5K9S	0	0	0	11	21	9	39	98							
	5K6S	0	0	0	6	11	16	11	24	43						
1X	5K12S	0	0	0	19	8	1	25	11	2	102					
	5K9S	0	0	0	7	15	5	10	16	7	32	69				
	5K6S	0	0	0	1	6	10	2	6	11	8	15	31			
SO	5K12S	0	0	0	18	10	2	23	9	4	21	8	2	62		
	5K9S	0	0	0	8	13	4	9	13	6	7	11	4	22	52	
	5K6S	0	0	0	2	5	6	3	7	8	0	3	6	4	10	21

a Model designations are explained in Sections 2.3 and 2.4.

**Table 7:** Number of Molecules with IC50 under 1 μM That Overlap between Different Libraries, *T* = 0.5^a^

*T* = 0.5, IC50 < 1000 nM		NoX			3X			2X			1X			SO		
		5K 12S	5K 9S	5K 6S	5K 12S	5K 9S	5K 6S	5K 12S	5K 9S	5K 6S	5K 12S	5K 9S	5K 6S	5K 12S	5K 9S	5K 6S
NoX	5K12S	288														
	5K9S	41	284													
	5K6S	46	56	326												
3X	5K12S	0	0	0	197											
	5K9S	0	0	0	44	156										
	5K6S	0	0	0	29	42	111									
2X	5K12S	0	0	0	22	18	22	197								
	5K9S	0	0	0	18	27	23	41	155							
	5K6S	0	0	0	22	29	51	39	49	139						
1X	5K12S	0	0	0	27	20	17	24	19	21	142					
	5K9S	0	0	0	16	30	24	21	28	29	34	140				
	5K6S	0	0	0	15	27	43	19	26	50	30	50	133			
SO	5K12S	0	0	0	28	20	16	21	21	20	23	16	13	77		
	5K9S	0	0	0	21	28	26	20	31	30	20	22	22	30	92	
	5K6S	0	0	0	14	21	34	17	25	38	13	17	34	20	31	98

a Model designations are explained in Sections 2.3 and 2.4.

**Table 8:** Average Properties of the Docking Subset of the 5 K, *T* = 0.5 Libraries Generated by Each Model^a^

*T* = 0.5, IC50 < 1000 nM		IC50 (nM)	QED	score (kcal/mol)	% similar pairs	% fluoro-phenyl	% decalin	% similar simvastatin	% similar atorvastatin
NoX	5K12S	384	0.29	–7.92	2.00	5.00	2.08	1.04	29
	5K9S	398	0.30	–7.75	3.00	4.00	1.41	0.35	18
	5K6S	397	0.29	–7.80	3.00	6.00	2.45	1.23	30
		**393**	**0.29**	***–7.82***	**2.67**	**5.00**	**1.98**	**0.87**	**25.39**
3X	5K12S	324	0.41	–7.58	62.00	16.00	11.17	28.93	10.08
	5K9S	281	0.41	–7.59	61.00	13.00	12.82	33.33	13.80
	5K6S	195	0.38	–7.76	58.00	5.00	9.91	44.14	22.31
		**267**	**0.40**	***–7.64***	**60.33**	**11.33**	**11.30**	**35.47**	**15.40**
2X	5K12S	243	0.39	–7.70	65.00	15.00	11.68	30.96	11.72
	5K9S	254	0.38	–7.67	56.00	14.00	10.97	30.32	12.75
	5K6S	195	0.40	–7.69	65.00	15.00	10.79	40.29	19.88
		**231**	**0.39**	***–7.69***	**62.00**	**14.67**	**11.14**	**33.86**	**14.78**
1X	5K12S	250	0.39	–7.60	45.00	12.00	11.97	33.10	12.23
	5K9S	243	0.39	–7.62	57.00	10.00	10.71	40.71	18.08
	5K6S	195	0.40	–7.64	70.00	12.00	11.28	45.11	24.42
		**229**	**0.39**	***–7.62***	**57.33**	**11.33**	**11.32**	**39.64**	**18.24**
SO	5K12S	248	0.42	–7.44	25.00	13.00	16.88	31.17	14.80
	5K9S	232	0.41	–7.50	33.00	13.00	15.22	35.87	15.29
	5K6S	129	0.41	–7.77	54.00	12.00	14.29	53.06	32.11
		**203**	**0.41**	***–7.57***	**37.33**	**12.67**	**15.46**	**40.03**	**20.73**

a Percent of library molecules with a given fragment, and percent of library molecules with Tanimoto similarity to known statins of 0.25 or greater. ***Bold italic*** rows show an average of the three models above them. Model designations are explained in Sections 2.3 and 2.4.

The 30 libraries in Tables 4 and 5 comprise 3695 total generated molecules with IC50 under 1 μm; 1160 of these were generated by the *T* = 0.0 models, and 2535 were generated by the *T* = 0.5 models. Of the 3695 molecules, there are 2183 *unique* molecules (59%), meaning that they exist in only one of the 30 libraries and are not duplicated in any of the other 29 libraries. Tables 6 and 7 show the number of molecules that are duplicated between each library. For both *T* = 0.0 and 0.5, the libraries generated with no transfer learning (NoX) have *no overlap* with any of the transfer-learning libraries or with the SO libraries. This indicates that the transfer learning with only ∼1000 molecules was sufficient to change the models significantly from the base model. For the *T* = 0.0 libraries, the largest overlap with other model/libraries (i.e., 3X models with 2X, 1X, SO models, etc.) is always with libraries with the same number of seed tokens, i.e., three X5K*9S* has its largest overlap with 2X5K*9S*, 1X5K*9S*, etc. This reiterates the fact that allowing the models to freely generate from a lower number of seeds leads to more duplication. The second most overlap comes from the next-closest, highest library. For example, for 3X5K9S, the greatest overlap with a 2X library is with 2X5K9S, but the second greatest overlap is with 2X5K12S, and the least overlap is with the 2X5K6S library.

The results for the *T* = 0.5 libraries in Table 7 show different behaviors from the *T* = 0.0 libraries. Not only do the *T* = 0.05 libraries produce more viable, submicromolar molecules than the *T* = 0.0 libraries, they also have less consistent overlap patterns. The largest overlap is not always with a library with the same number of seed tokens (see 2X5K6S overlapping with the SO libraries, for example). This is attributable to the less deterministic nature of the *T* = 0.5 generation process.

As the *T* = 0.5 models have been shown to produce more robust libraries than the *T* = 0.0 models, only data for the *T* = 0.5 libraries will be shown here. The *T* = 0.0 information can be found in the Supporting Information. Table 8 shows the average properties for the 15 *T* = 0.5 5 K libraries (more properties are available in the Supporting Information). The average IC50 values for these libraries generally reflect the trends in the 1 K libraries (Supporting Information), except that in the 5 K libraries, the IC50 values decrease monotonically with decreasing number of pretrained layers, with the SO5K6S *T* = 0.05 library having a very low 129 nM average IC50 (due to a more statistically significant sample). These data clearly show that less-pretrained layers lead to more statin-like molecules. The NoX libraries have IC50 averages 60–100 nM higher than any fine-tuned library. The 5 K libraries also have a fairly consistent QED value of 0.39/0.40 (agreeing with the ChEMBL1081 training set’s value of 0.39), while the 1 K libraries varied between 0.4 and 0.45. This is most likely due to the 5 K libraries sampling a larger portion of the chemical space than the 1 K libraries. Average molecular weights also agree with the 1 K libraries, with values between about 475 and 485 g/mol. The average *A* log *P* values for the 5 K libraries are lower than the 1 K libraries in all cases, and molecular weights are slightly higher, which may explain the fact that docking scores are slightly lower for the 5 K libraries as well, since docking scores tend to favor larger molecular weights.^25^ All docking scores are between the averages for type-1 and type-2 statins found in this work (−8.5 and −7.4 kcal/mol, respectively). Numbers of rotatable bonds are also slightly higher, again likely owing to larger molecular weights as well. In all cases, the *T* = 0.5 libraries have a higher percentage of similar pairs, though in the *T* = 0.0 libraries, the ones with more seed tokens have a larger percent similar pairs, and for *T* = 0.5, the ones with fewer seed-tokens have a larger percent similar pairs. Overall, between the *T* = 0.0 and *T* = 0.5 libraries, most properties are similar, but the *T* = 0.5 libraries have lower average IC50 values for all models. Further, in the *T* = 0.5 libraries, the IC50 decreases with decreasing prompt length in every model except the NoX model, which has nearly constant, much higher IC50 values for all prompt lengths, suggesting that the lack-of fine-tuning is expressed more in higher temperatures. The *T* = 0.5 models also generate more unique, submicromolar molecules, and so the higher temperature option appears more favorable.

Table 8 shows the percentage of each *T* = 0.5 library that contains the four fragments common in HMGCR inhibitors (fragments not included here are in the Supporting Information). The percentages for all libraries are generally higher than what was found for the 1 K libraries, with the NoX model producing 3–13% of molecules containing the fragments. These percentages increase considerably in all of the transfer-learning libraries for the atorvastatin pharmacophore, from ∼2% in the *T* = 0.0 NoX libraries to 30–35% in the *T* = 0.0 transfer-learning libraries. The *T* = 0.5 libraries start with a higher percentage of this fragment (11%) but the transfer-learning libraries increase to 40–54%. Fluorophenyl also starts with a low percentage in the NoX libraries (1–3% for *T* = 0.0 and 0.5) and increases to 40–60% with transfer learning. The butyryl fragment starts with about 4% in the *T* = 0.0 NoX libraries, increases modestly to 6–9% with transfer learning, and starts with 13% in the *T* = 0.5 NoX library and decreases for all transfer-learning libraries. The decalin fragment start with 0 and 5% in the *T* = 0.0 and *T* = 0.5 NoX libraries and increases to 10–20% with transfer learning. Type-2 statin-like molecule fragments are clearly present in larger fractions than the type-1 statin-like molecule fragments. This is further reflected in the percent Tanimoto similarity to statins, also shown in Table 8 (statin similarities not included here are in the Supporting Information). The NoX libraries have very little similarity to the statins at 0–2%, while the transfer learning libraries have 10–20% similarity to simvastatin (type-1) and 30–40% similarity to atorvastatin and rosuvastatin (type-2 statins).

Table 9 shows Pearson correlation coefficients for several properties of the 5 K libraries at *T* = 0.5. The higher temperature model has proven to generate more robust libraries, and so it is the focus of this discussion, but the same information for *T* = 0.0 can be found in the Supporting Information. For the 3X-, 2X-, and 1X-based libraries, the ln-IC50/docking score correlation always increases monotonically with decreasing numbers of seed tokens, going from moderate correlation with 12S to strong correlation with 6S. This indicates the 6S models are better able to generate viable HGMCR inhibitors than the 9S and 12S models. The NoX libraries have inconsistent correlations for this pair of properties, and the SO libraries show moderate correlations that fluctuate between 0.40 and 0.50. *t* tests showed that the ln-IC50/score correlation was statistically significant in almost every instance for the transfer-learning libraries, although not for the NoX-based libraries. The only other property pair shown here that has consistent correlation is docking score with the number of aromatic rings, which is supported by *t* tests that show these correlations are statistically significant. This is a moderate-to-strong inverse correlation that almost always increases with decreasing number of seed tokens. More correlations and *t*-test outcomes are shown in the Supporting Information, including correlations between ln-IC50 and atorvastatin similarity and docking score and atorvastatin similarity, both of which have a medium-to-strong correlation and are statistically significant.

**Table 9:** Pearson Correlation Coefficients for Various Properties of the Docking Subset of the 5 K Libraries Generated by Each Model at *T* = 0.5^a^

*T* = 0.5, IC50 < 1000 nM		ρ(ln-IC50/score)	ρ(ln-IC50/QED)	ρ(ln-IC50/ALogP)	ρ(score/QED)	ρ(score/rings)
NoX	5K12S	0.02	0.14	–0.16	0.31	–0.59
	5K9S	0.00	0.13	–0.27	0.31	–0.60
	5K6S	0.04	0.04	–0.22	0.40	–0.68
		**0.02**	**0.10**	***–0.22***	**0.34**	***–0.62***
3X	5K12S	0.34	0.14	–0.17	0.09	–0.44
	5K9S	0.40	0.13	–0.29	0.00	–0.40
	5K6S	0.56	0.29	–0.39	0.12	–0.64
		**0.43**	**0.19**	***–0.28***	**0.07**	***–0.49***
2X	5K12S	0.22	0.15	–0.17	0.16	–0.34
	5K9S	0.38	0.14	–0.25	0.17	–0.39
	5K6S	0.61	0.22	–0.27	0.21	–0.64
		**0.40**	**0.17**	***–0.23***	**0.18**	***–0.46***
1X	5K12S	0.32	0.23	–0.06	0.15	–0.50
	5K9S	0.54	0.25	–0.27	0.12	–0.63
	5K6S	0.66	0.35	–0.31	0.17	–0.73
		**0.51**	**0.28**	***–0.21***	**0.15**	***–0.62***
SO	5K12S	0.47	–0.01	–0.17	0.08	–0.60
	5K9S	0.45	0.18	–0.20	0.19	–0.53
	5K6S	0.55	0.14	–0.35	0.22	–0.71
		**0.49**	**0.10**	***–0.24***	**0.16**	***–0.61***

a Score = docking score; rings = number of aromatic rings. ***Bold italic*** rows show an average of the three models above them. All statistically significant correlations are underlined. Model designations are explained in Sections 2.3 and 2.4.

### 3.5 Cluster Analysis of Molecules Generated in the 5 K Libraries

In order to analyze the chemical space sampled by the 2183 unique molecules with submicromolar IC50 values, they were separated into 10 clusters using k-means cluster analysis in SciKitLearn.^16^ RDKit descriptors were used as features for the cluster analysis, and 3, 5, and 10 cluster groups were tested, with 10 resulting in the most meaningful groupings. Mordred descriptors were also tested as features for clustering with 5 and 10 cluster groups, but this did not result in a satisfactory classification. A set of six known statins was then classified into the existing 10 clusters. Table 10 shows some properties for the 10 cluster groups as well as the known statins in each group or, if the group does not have a representative statin, the general class of molecules to which the group is most similar.

**Table 10:** Properties of the 10 Groups Found by Classifying the 2183 Unique Submicromolar Molecules Found via the GPT Models^a^

	number of molecules	mean IC50 (nM)	median IC50 (nM)	mean score (kcal/mol)	mean QED	known statin or related class
group 0	340	352	296	–7.56	0.15	**flavonoids**
group 1	236	432	432	–7.40	0.50	**steroids**
group 2	254	469	457	–6.52	0.53	**miscellaneous**
group 3	403	289	149	–6.84	0.40	**Simvastatin, Lovastatin, Pravastatin**
group 4	307	143	19	–7.51	0.45	**Fluvastatin**
group 5	58	154	9	–7.98	0.34	**Rosuvastatin**
group 6	74	274	215	–6.39	0.17	**polyalkenes/terpenoid**
group 7	129	382	289	–6.17	0.10	**oligopeptides**
group 8	228	470	402	–7.71	0.34	**flavonoid/quinolone**
group 9	154	105	13	–7.53	0.23	**Atorvastatin**

a The last column lists known statins classified into each group or the general class of molecules the group most resembles.

Groups 4, 5, and 9 have a lower mean IC50 value than the other groups, but because these molecules were classified into clusters via an unsupervised learning method, the mean can be susceptible to outliers. Thus, the median value may be more informative. Groups 4, 5, and 9 do have considerably lower median IC50 values than the other groups, with group 5 having the lowest value, but the median values show that group 3 is also below the other groups. Groups 0, 1, 4, 5, 8, and 9 have lower docking scores than the other groups (−7.5 to −8 kcal/mol), but groups 0, 1, and 8 have high median and mean IC50 values. Thus, from this analysis, it can be concluded that groups 4, 5, and 9 are the best candidates for HMGCR inhibitors, and indeed, when the known statins are classified into the cluster groups, the three type-2 statins are in groups 4, 5, and 9. All three type-1 statins fall into group 3, which also has a lower IC50 value. The relative IC50 and docking score values for these groups make sense, as the type-2 statins are generally more potent than type-1 statins, and rosuvastatin (group 5) is the most potent statin.

**Figure 6:**
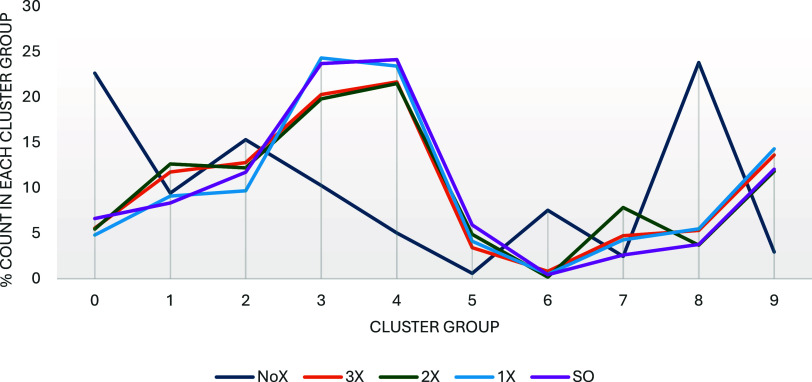
Average percentage of molecules in each k-means cluster group for each of the screening libraries with *T* = 0.5 (averaged over prompt length; the same table for *T* = 0.0 can be found in the Supporting Information).

When the 30 libraries are classified into these groups individually, the breakdown of what types of molecules comprise each library can be seen. The Supporting Information shows exact counts for molecules in each group for every library, and Figure 6 shows the % of molecules for each transfer-learning approach in each group for *T* = 0.5. The values in Figure 6 are averaged over prompt length, since they do not vary greatly or consistently for the 6, 9, and 12 token prompts. It can be seen that the NoX libraries have the most molecules in group 0, with high percentages in groups 1 and 2, and the second highest peak in group 8. None of these groups are statin-like, and in fact, almost half of the NoX libraries are flavonoid-like. By contrast, all of the libraries with transfer learning have their peaks in groups 3, 4, and 9: all statin-like groups. In general, all of the transfer-learning libraries have similar behavior, which is distinct from the NoX libraries. Interestingly, the transfer-learning libraries have relatively low percentages for the rosuvastatin-like group (3–6%, group 5), but the NoX libraries have only 1% for group 5, so the transfer learning does have an effect on that group. The same figures for the *T* = 0.0 libraries are presented in the Supporting Information and largely follow the same patterns.

Two-tailed *t* tests with 95% confidence (*p* = 0.05) were performed to assess whether the groups could be deemed independent of each other based on the mean IC50 values and the standard deviations. Group 3 (type-1) is independent of groups 4, 5, and 9 (all type-2), but groups 4, 5, and 9 were shown to not be statistically different. This makes sense as all three groups represent type-2 statins. Interestingly, group 6 was shown to be statistically the same as group 3, despite having a considerably lower mean IC50 and docking score. Groups 1, 2, and 8 were shown to be statistically similar as were groups 0 and 7. There are 307, 58, and 154 molecules in groups 4, 5, and 9, meaning there are about 519 potential type-2 statin molecules in the libraries, and there are 403 molecules in group 3, meaning there are about 403 potential type-1 statins in the libraries. This means that about 24% of the total unique molecules are type-2 statin-like molecules and about 18% are type-1 statin-like molecules, so overall 42% of the generated molecules have potential to be good HMGCR inhibitors and statin-like.

**Figure 7:**
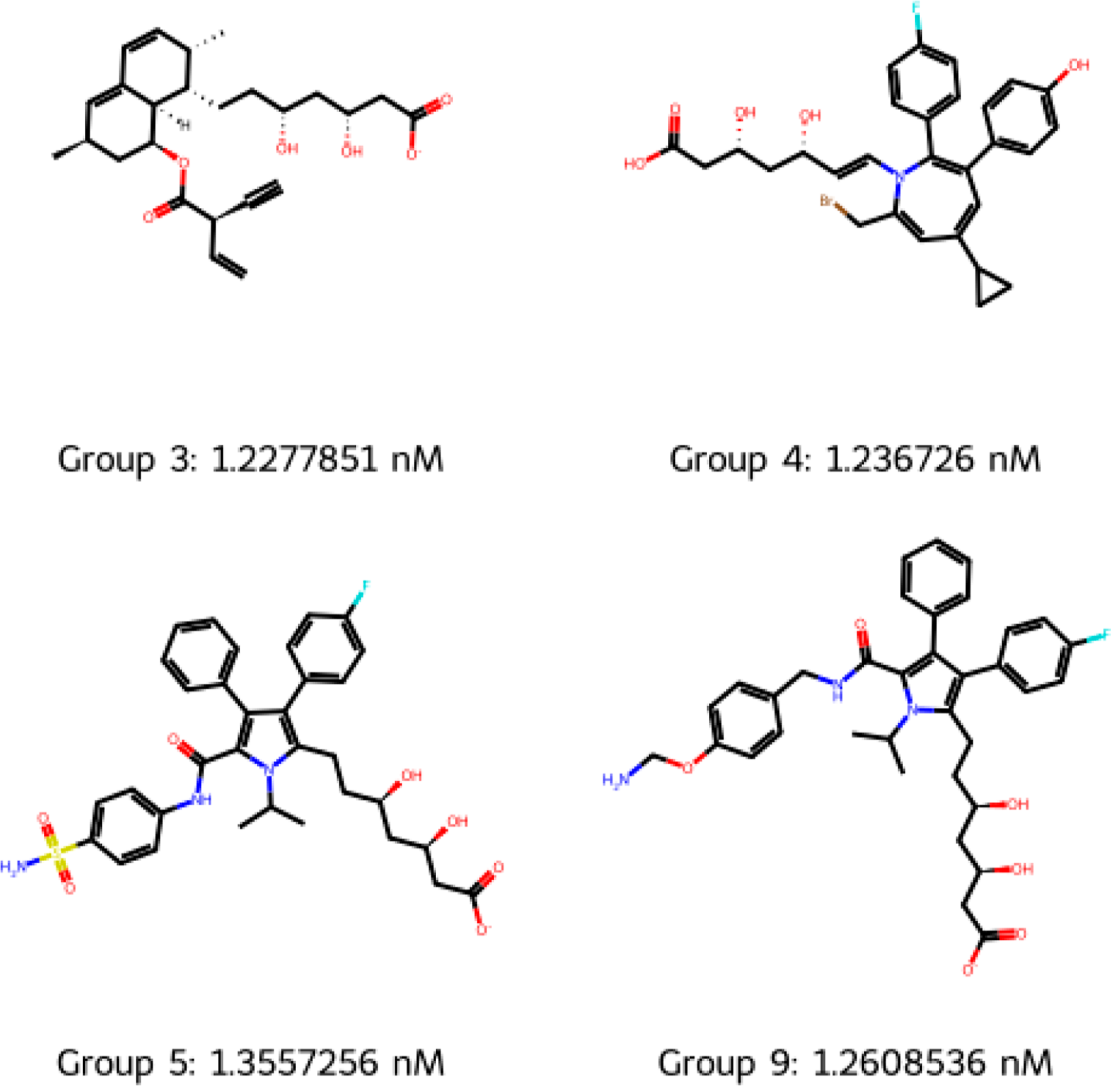
Representative molecules from the 3 k-means clusters associated with known statins. Molecule shown has the lowest IC50 value in each group.

**Figure 8:**
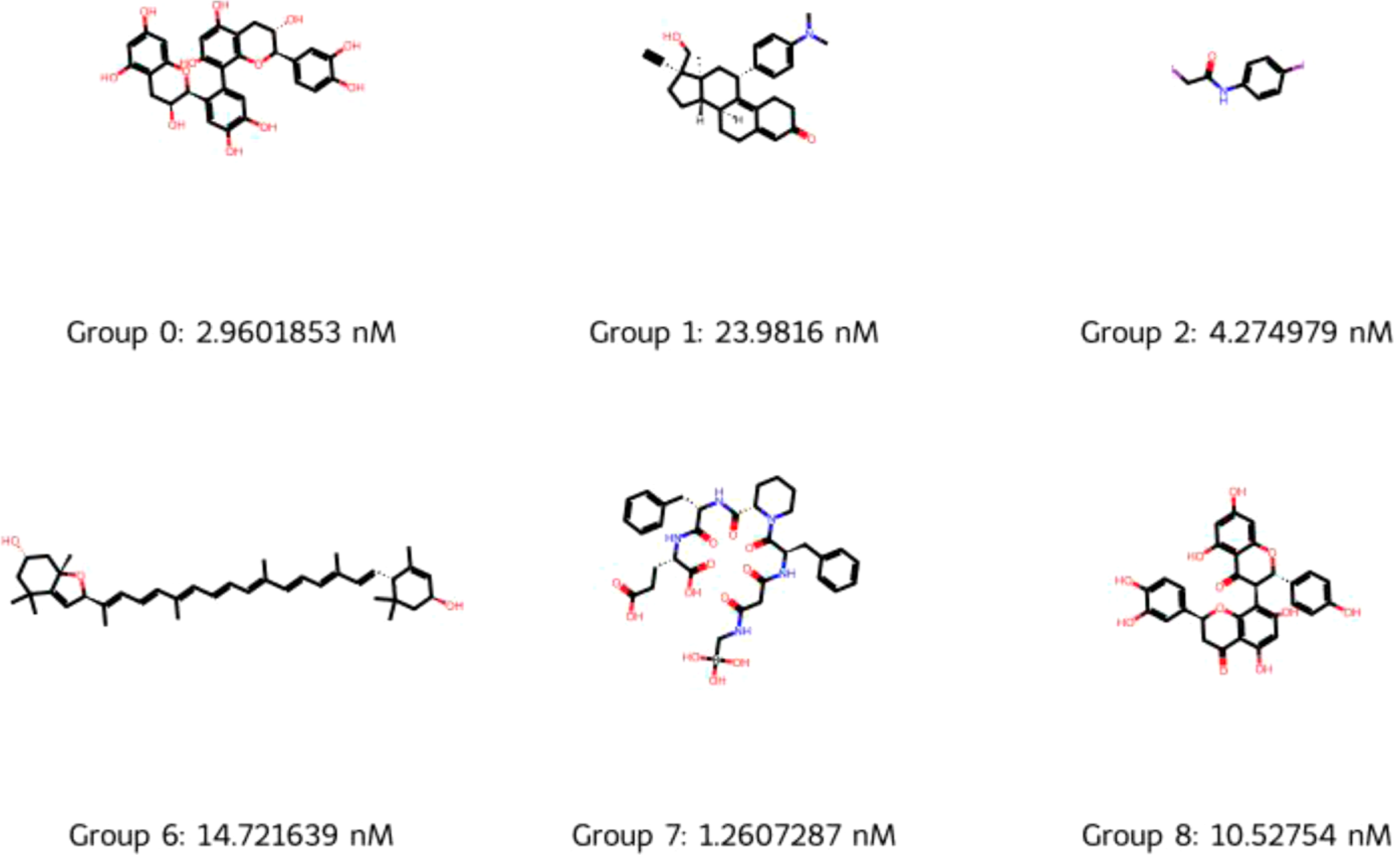
Representative molecules from groups 0, 1, 2, 6, 7, and 8 with the lowest IC50 values.

**Figure 9:**
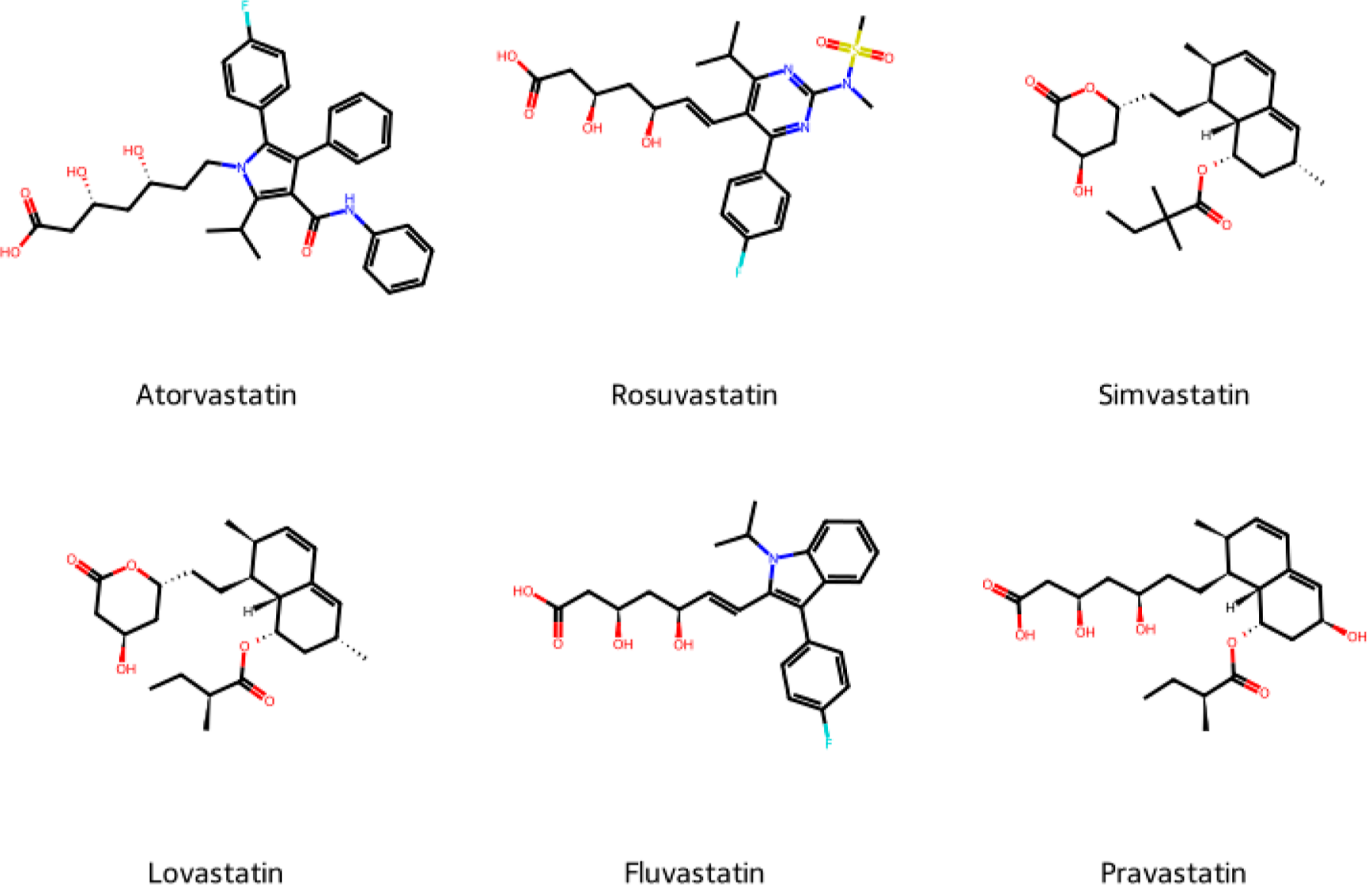
Structures of six known statins.

Figure 7 shows a representative molecule from each statin-containing group (groups 3, 4, 5, and 9) with the lowest IC50 values, while Figure 8 shows a representative molecule from each of the other groups. It is clear why the known statins (Figure 9) were classified into each group. Many group 3 molecules contain the decalin and the butyryl group characteristics of type-1 statins, while some have the atorvastatin pharmacophore (3,5-dihydroxypentanoic acid) and others have the simvastatin pharmacophore (4-hydroxytetrahydro-2*H*-pyran-2-one). Molecules from groups 4, 5, and 9 almost always contain a fluorophenyl group, indicative of type-2 statins. Group 4 is distinguished by having a carbon–carbon double bond either adjacent to or in close proximity to the pharmacophore (usually 3,5-dihydroxypentanoic acid); group 5 is distinguished by almost always having a methyl sulfonamide group; and group 9 has the 3,5-dihydroxypentanoic acid pharmacophore connected to a pyrrole group.

It can be seen in Figure 8 that groups 3 and 6 are not structurally similar, despite the *t*-test showing that their IC50 values are statistically similar. Group 6 is distinguished by long conjugated chains (polyalkenes) not present in group 3. Groups 0 and 8 do appear structurally similar (both are flavonoid-like), though the *t*-test showed them to be statistically different; group 8 could be classed as quinolone derivatives. Groups 0 and 7 were shown to be statistically similar, and structurally they both contain molecules with many polar groups, but group 7 molecules are primarily oligopeptide-like and group 0 molecules are flavonoids. Groups 1, 2, and 8 were shown to be statistically similar, but structurally, group 1 is steroid-like, group 8 is flavonoid/quinolone-like, and group 2 molecules appear to be a grouping of various “polar” molecules. Overall, the cluster analysis, supported by Table 10 and Figures 7 and 8, shows that the GPT model does sample a wide variety of chemical spaces. While 42% of molecules are at least somewhat “statin-like”, molecules with submicromolar IC50 values are found from the flavonoid, terpenoid, steroid, quinolone, oligopeptide families, and 254 molecules (∼12%) miscellaneous/unclassifiable molecules were also found to have submicromolar potency.

### 3.6 Docking Poses for Selected Molecules

**Figure 10:**
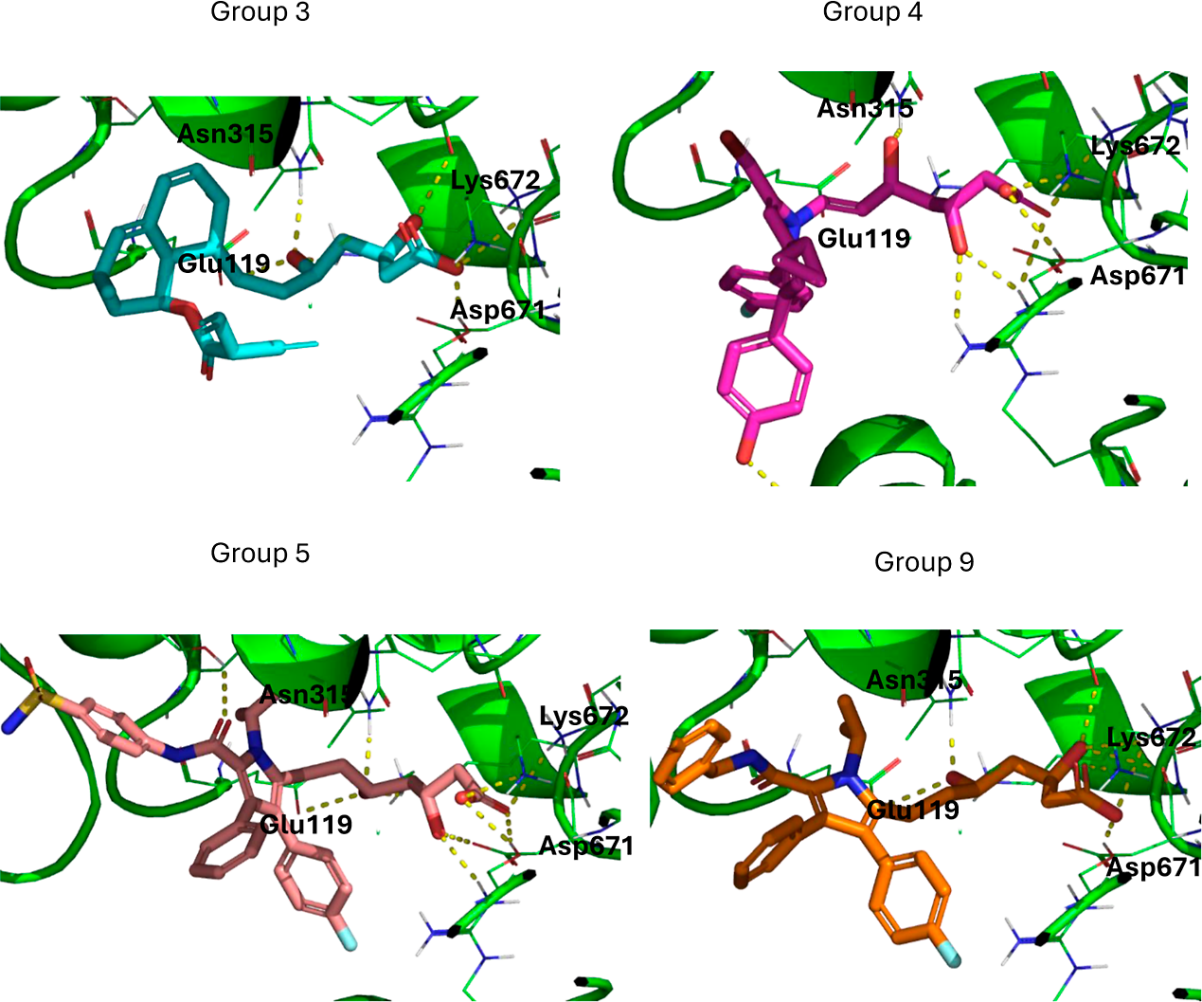
Lowest energy docking poses in HMGCR for the molecules from groups 3, 4, 5, and 9 with the lowest predicted IC50 values.

Docking poses for the molecules from groups 3, 4, 5, and 9 (the statin-like groups) with the lowest IC50 values are shown in Figure 10. In all cases, the pharmacophore forms hydrogen, ion-dipole, or ion–ion bonds with Lys672 and Asp 671 as the natural substrate HMG-Coenzyme A does. However, while the molecule groups 3, 5, and 9 form hydrogen bonds with Asn315 and Glu119, the molecule from group 4 only forms a bond with Asn315, and it appears strained. The group 4 molecule also sits in the active site differently, with the bulk of the molecule pointed out of the binding site, while the groups 3, 5, and 9 molecules sit more tightly in the whole active site. This may influence the fact that group 4 has a higher median IC50 value and a higher docking score than the other type-2 groups. Docking poses for the top molecules from the other six groups as well as poses for six known statins are available in the Supporting Information.

### 3.7 Synthetic Accessibility Score

**Figure 11:**
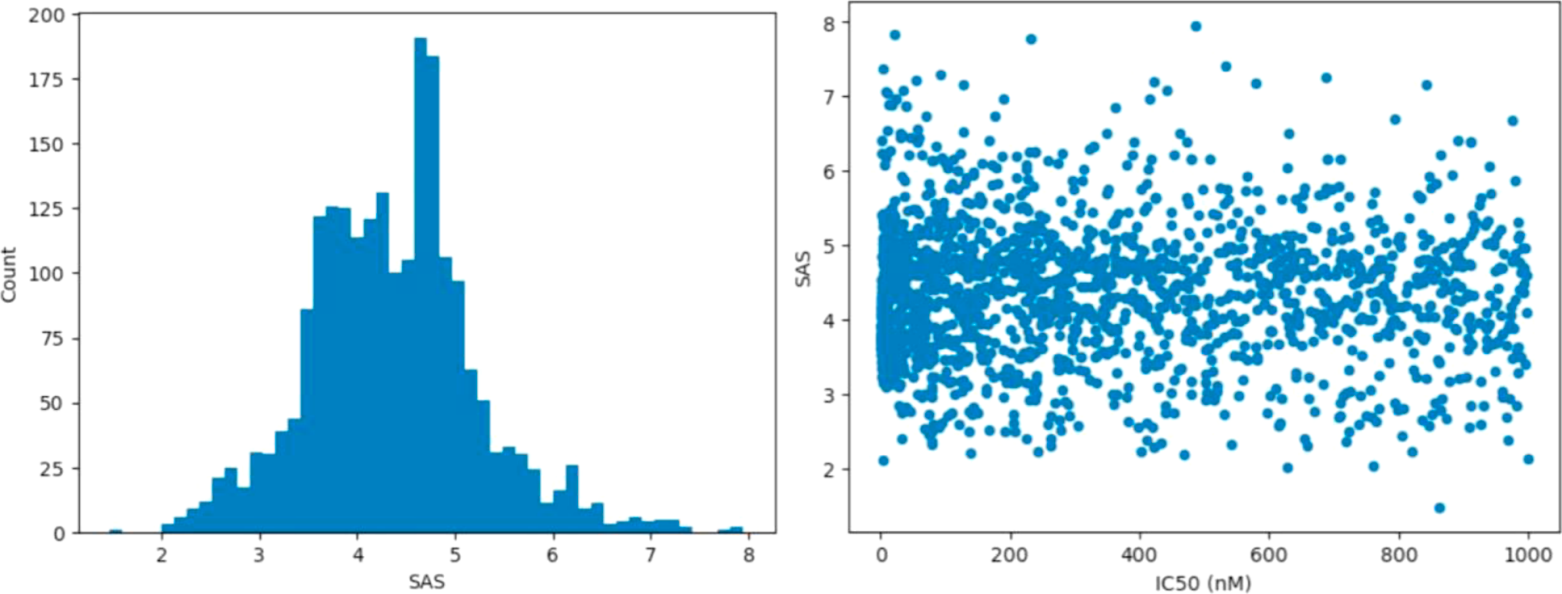
(left) Distribution of the SAS for the set of 2183 unique submicromolar molecules generated by all libraries and (right) SAS versus predicted IC50 values (nM) for the same molecule set.

The final set of 2183 unique submicromolar molecules was assessed for ease of synthesis using the SAS^31^ implemented in RDKit. In this scoring scheme, a value of 1 indicates relatively simple estimated synthesis, and a value of 10 indicates a very difficult/impossible synthesis. Figure 11 shows the histogram of SAS values for the set classified into 50 bins. The distribution is roughly Gaussian, with a mean of 4.4, a median of 4.4, and a standard deviation of 0.9. For comparison, the set of six statins, including atorvastatin, rosuvastatin, fluvastatin, simvastatin, lovastatin, and pravastatin has an average SAS of 4.0, with the highest value belonging to simvastatin at 4.7 (although in practice, this statin is only a synthetic modification of a natural product,^33^ so the true synthetic ease of making simvastatin is likely lower), and the lowest belonging to atorvastatin and fluvastatin at 3.3. There is no correlation between SASs and predicted IC50 values, meaning that potent molecules are no more difficult or easy to synthesize than less potent molecules (Figure 11).

## 4 Conclusions

It has been shown that pretraining of transformer-decoder-based ML models and fine-tuning/transfer learning can be good strategies for creating libraries of molecules for virtual screening for activity for a particular enzyme. Having more pretrained layers in the generative model leads to more stable models and produces a higher percentage of valid molecules, but having more fine-tuned layers in the model leads to molecules more suited to the enzyme (lower IC50 values and presence of more desired moieties). The percentage of valid molecules can also be increased by varying the prompt length, with the increase as large as 50% of the total (from 35 to 85% of valid molecules by decreasing prompt length). Correlation analysis shows that the best correlation between predicted ln-IC50 values and docking scores is produced by the 1X models, which have one pretrained layer and 3 fine-tuned layers. This implies that the best behavior for the models may require a baseline of general learning but that the bulk of the model can be fine-tuned. Shorter prompt lengths also result in better ln-IC50/docking correlations in all cases but one.

The higher temperature model produces more “refined” molecules and more submicromolar molecules. The higher temperature model also produces a higher proportion of molecules with the desired fragments and produces molecules with higher Tanimoto similarity to known statins. At the higher temperature, the smallest prompt length (6 seed tokens) produces molecules with the lowest average IC50 values, the lowest average docking score (with one exception), the strongest correlation between predicted IC50 and docking score, the highest proportions of desired fragments, and the highest proportions of molecules similar to known statins. Thus, the 1X model with 6 seed tokens at *T* = 0.5 is recommended as the best model to generate libraries for virtual screening. K-means clustering using RDKit descriptors was also effective at separating the generated molecules into the different types of statin as well as other molecule classes, allowing an easy method of choosing the most promising candidates for further testing.

## Data Availability

The data for this work is provided as supporting data for this article, as well as in the data repository at the University of Reading. All work was completed with freely available software, and data can be accessed with freely available software: Python and all libraries can be accessed with Anaconda (https://www.anaconda.com/) or Google Colab. PyMOL can be accessed via the website (https://pymol.org/) or via Anaconda.

## Supporting Information Available

The following data is provided as Supporting Information for this article, as well as in the data repository at the University of Reading: raw data tables for the properties of the “docking” libraries shown here as well as some properties for the larger “refined” libraries. Further properties are shown, including additional fragment analysis, similarity analysis, and more correlations, as well as results of *t* tests for statistical significance (XLSX)
Analysis of 1K libraries, including information on molecules generated, properties, and correlations (PDF)
CSV files of all generated libraries are provided, along with a TKinter-based GUI for exploring the libraries. Docking poses for molecules from each cluster group with the lowest IC50 value are provided, as well as poses for six known statins (ZIP)
