# Supplementary material for: Transformer-Decoder GPT Models for Generating Virtual Screening Libraries of HMG-Coenzyme A Reductase Inhibitors: Effects of Temperature, Prompt Length, and Transfer-Learning Strategies
Source: J Chem Inf Model. 2024 Nov 7;64(22):8464–80. doi: 10.1021/acs.jcim.4c01309 (PMC11600504; doi:10.1021/acs.jcim.4c01309)
Supplement: Supplementary file 2 — ci4c01309_si_002.pdf [file ci4c01309_si_002.pdf]

**Transformer-decoder GPT models for generating virtual screening libraries of HMGCR inhibitors: effects of temperature, prompt-length and transfer-learning strategies**

Mauricio Cafiero

School of Chemistry, Food and Pharmacy, University of Reading, Reading, UK, RG6 6AD

**Supporting Data 2: discussion of proof-of-concept 1000 prompt libraries.**

***figure numbers refer to the manuscript.**

Even before screening for pharmaceutical activity, one of the first questions to ask of these GPT models is: can they generate a robust library of molecules? Table S2.1 shows the numbers of valid molecules generated by each fully-trained model, along with how many were duplicates, and how many simply replicated the seed molecules. The models fully trained on the ZN1540K dataset, *i.e.* those models which were able to more fully learn the language of SMILES strings (NoX), had 89 and 84% valid molecules at T=0.0 and 0.5. Noguchi and Inoue reported 60 and 90% valid molecules for their PixelCNN and RNN models trained on 250,000 molecules from the ZINC database,[8] showing that using the smaller training dataset here did *not* greatly affect percent validity compared to those models. Though Urbina *et al* do not report exact numbers for their percent validity, examination of their Figure 2 shows that they achieved between 93 and 97% validity for their RNN, VAE (variational autoencoder) and GAN (generative adversarial network) models trained on > 1 million molecules from ChEMBL, again showing small improvement in percent valid molecules for a large increase in dataset size. More closely related to this work, the percent validity reported by Rishal *et al* for a transformer-decoder was 99% with a training set of > 1 million molecules. Below, this work will show improvement in percent validity by using different prompts or changing temperature rather than by using a larger training set. It is clear than the more blocks that were trained on the ZN1540K dataset, the more the models learned how to put together a valid SMILES string. "Invalid" molecules were those rejected for having a non-sensical SMILES string. This manifested most often as an unclosed ring, un-matched parenthesis, or similar issues. The SO model (with no ZN1540K training) could only generate 35% valid SMILES (these are for T=0.0, but T=0.5 is similar) due to the lack of generalized training. The other models fell in-between the SO and NoX models.

The next step in refining the libraries is to remove duplicate molecules. The amount of duplicates for T=0.0 models did not vary considerably between models, with all five models have between 13 and 23% duplicates. An interesting trend is that in every case, the T=0.5 models had less duplicates, and, for the NOXALL model, the T=0.5 models has *considerably* fewer duplicates, suggesting that creating robust libraries may be easier with higher temperature models. This makes sense as the T=0.5 libraries are by definition *less probable* molecules. Finally, the libraries are refined by removing generated molecules that replicated seeds, as these molecules are already "known." It is interesting to note that the model which replicated seeds with the highest percentage was the NoXALL model, which had only pre-training. All other models other than SO were pre-trained and fine-tuned and had % seeds around half of the NoXALL model. The refined libraries had between 75% and 20% total return on prompts after removing duplicates and seeds, meaning that for 1000 prompts, they created 750 to 200 generated molecules. This may be compared to the value of 63% reported by Yang *et al* for the same analysis (removing invalid, duplicate and seed molecules).[6] The models with more pre-trained blocks had higher total prompt return. When the "Docking" library was created by keeping only molecules with predicted IC50 values under 1 μM, the prompt return drops to between 2 and 7 percent, with T = 0.5 always generating more molecules.

**Table S2.1.** Number of molecules generated by each model at T = 0.0 and 0.5 with duplicates and seeds removed. % is compared to the 1000 prompts.

| | | Molecules Generated | % | After removing duplicates | % | After removing seeds | % | IC50 < 1000 nM | % |
|---|---|---|---|---|---|---|---|---|---|
| **NoX1K12S** | **0.0** | 886 | 88.60 | 648 | 64.80 | 588 | 58.80 | 52 | 5.20 |
| | **0.5** | 839 | 83.90 | 817 | 81.70 | 750 | 75.00 | 71 | 7.10 |
| **3X1K12S** | **0.0** | 709 | 70.90 | 492 | 49.20 | 466 | 46.60 | 48 | 4.80 |
| | **0.5** | 689 | 68.90 | 555 | 55.50 | 528 | 52.80 | 57 | 5.70 |
| **2X1K12S** | **0.0** | 669 | 66.90 | 464 | 46.40 | 438 | 43.80 | 48 | 4.80 |
| | **0.5** | 671 | 67.10 | 535 | 53.50 | 512 | 51.20 | 63 | 6.30 |
| **1X1K12S** | **0.0** | 547 | 54.70 | 362 | 36.20 | 340 | 34.00 | 32 | 3.20 |
| | **0.5** | 543 | 54.30 | 402 | 40.20 | 380 | 38.00 | 36 | 3.60 |
| **SO1K12S** | **0.0** | 345 | 34.50 | 215 | 21.50 | 198 | 19.80 | 22 | 2.20 |
| | **0.5** | 292 | 29.20 | 214 | 21.40 | 196 | 19.60 | 25 | 2.50 |

Table S2.2 shows selected properties for the docking subset of each generated library. The first column shows the average IC50 values for all molecules in the subset (*i.e.* molecules with IC50 less than 1 μm). The values generally decrease from the NoXALL model to the SO model due to increasing numbers of layers trained with HMGCR inhibitors, though even the SO model (195 nM) does not reach the IC50 values of the training set (93 and 60 nM, Manuscript/Table 2). The 1XALL model does increase to values between the 3XALL and 2XALL values, and the SO, T = 0.5 library is also an outlier in the trend. Yang *et al*, who also performed fine-tuning for a specific target after pre-training a transformer-based model, did not report improvement on IC50 due to fine-tuning, only reporting the final values. QED values are consistently around 0.4-0.45 in all models that used transfer learning, which is in-line with the training set values of 0.46 and 0.39 (Table 2) and higher than the no-transfer learning model (0.33 and 0.29). Molecular weight is also consistently between 460 and 480 g/mol for all models with transfer learning, and above 500 g/mol for the NoXALL model. It is notable that the transfer-learning models had an average molecular weight in line with the BDB905 dataset, which all had IC50 values, and the no-transfer-learning model was more in line with the ChEMBL1081 dataset, which did not all have IC50 values. Docking scores are more difficult to differentiate between the models, as they only vary between about -5 and +10.5 kcal/mol across the entire range, but while all models are relatively consistent, 1XALL has slightly lower scores of -7.74 and -7.70 kcal/mol, which are closer to the training dataset values of -7.95 and -7.90 kcal/mol than the other models. All libraries had average scores between the average for type-1 and type-2 statins reported above. Statin molecules tend to favour lower alog P values for better hepatoselectivity,[18] but the training sets had values of 4.0, which is close to the averages for the transfer-learning models here. The NoXALL model has a considerably lower alog P at about 3.10. Rotatable bonds can serve as an indication of “flatness,” which is in turn an indicator that a molecule is less likely to be toxic.[27] This property is also variable between all models, between about 6.5 to 8 rotatable bonds per molecule; the training sets had an average of about 9. The percent of similar pairs can be used as an indicator of the variety within the library: a library with higher percent similar pairs will have many molecules

in the same chemical space, while a library with lower similar pairs will sample a larger chemical space. Here, models trained with one dataset (NoXALL and SO) have ~20% similar pairs, while the mixed-dataset models have closer to 10% similar pairs. The 2XALL model is an exception here. The training sets had a percent similarity of 55% and 30%, indicating that the generated libraries had greater variety than the training sets.

**Table S2.2.** Average properties of the docking subset of the 1K libraries generated by each model at T = 0.0 and 0.5. MW = Molecular weight.

| | T | IC50 (nM) | QED | MW (g/mol) | aLog P | Score (kcal/mol) | Rotatable Bonds | % Similar pairs |
|---|---|---|---|---|---|---|---|---|
| **NoX1K12S** | **0.0** | 384 | 0.33 | 506 | 3.13 | -7.60 | 6.60 | 19.61 |
| | **0.5** | 425 | 0.29 | 523 | 3.10 | -7.71 | 7.32 | 20.44 |
| **3X1K12S** | **0.0** | 291 | 0.45 | 466 | 3.77 | -7.68 | 7.60 | 11.70 |
| | **0.5** | 326 | 0.43 | 461 | 3.71 | -7.69 | 7.52 | 7.96 |
| **2X1K12S** | **0.0** | 215 | 0.43 | 480 | 4.20 | -7.74 | 7.70 | 20.12 |
| | **0.5** | 204 | 0.39 | 485 | 4.00 | -7.70 | 8.00 | 14.03 |
| **1X1K12S** | **0.0** | 268 | 0.44 | 471 | 4.36 | -7.79 | 6.47 | 8.47 |
| | **0.5** | 255 | 0.39 | 494 | 3.54 | -7.70 | 7.90 | 9.37 |
| **SO1K12S** | **0.0** | 195 | 0.41 | 478 | 4.43 | -7.68 | 8.09 | 17.32 |
| | **0.5** | 228 | 0.43 | 465 | 4.39 | -7.53 | 7.76 | 18.67 |

Table S2.3 shows Pearson correlation coefficients for several properties for each of the generated libraries(more correlations are shown in the supporting data). The DNN-predicted ln-IC50 values and the docking scores generally have positive, medium correlations, meaning that a lower ln-IC50 is correlated with a lower docking score; the SO, T = 0.5 library has a strong correlation and the NoX, T = 0.0 library has a weak correlation. This correlation is largely stronger than that found for the trainings sets, which had a weak/medium correlation of 0.25. T-tests show that the transfer-learning-based libraries' correlations are statistically significant, while the correlations for the NoX libraries are split. ln-IC50 and QED have medium correlations in most cases, with the SO model being an exception. This is an interesting case, as a better (lower) ln-IC50 value correlates with a lower (less-druglike) QED score. This agrees with the medium/strong correlation of 0.47 found for ln-IC50 and QED in the BDB905 training set (though the ChEMBL1081 training set has weak correlation). T-tests show that this correlation is only sporadically statistically significant. The ln-IC50/alog P correlation is weak for these libraries, as in the training sets, but both the docking score/QED and docking score/number of aromatic rings correlations are medium-to-strong for most libraries, though only the docking score/number of rings correlation is statistically significant for most libraries.

**Table S2.3.** Pearson correlation coefficients for various properties of the docking subset of the 1K libraries generated by each model at T = 0.0 and 0.5. score = docking score; rings = number of aromatic rings. All statistically significant correlations are underlined.

| | T | ρ(ln-IC50/score) | ρ(ln-IC50/QED) | ρ(ln-IC50/aLogp) | ρ(Score/QED) | ρ(Score/rings) |
|---|---|---|---|---|---|---|
| **NoX1K12S** | **0.0** | 0.10 | 0.29 | -0.35 | 0.37 | -0.62 |
| | **0.5** | 0.27 | 0.23 | -0.06 | 0.36 | -0.51 |
| **3X1K12S** | **0.0** | 0.47 | 0.26 | -0.04 | 0.43 | -0.43 |
| | **0.5** | 0.28 | -0.01 | -0.10 | 0.20 | -0.43 |
| **2X1K12S** | **0.0** | 0.46 | 0.39 | -0.14 | 0.51 | -0.30 |
| | **0.5** | 0.48 | 0.17 | -0.21 | 0.32 | -0.30 |
| **1X1K12S** | **0.0** | 0.32 | 0.48 | -0.19 | 0.04 | -0.10 |
| | **0.5** | 0.44 | 0.26 | -0.13 | 0.19 | -0.33 |
| **SO1K12S** | **0.0** | 0.46 | 0.00 | 0.13 | 0.38 | -0.65 |
| | **0.5** | 0.57 | 0.10 | 0.05 | 0.34 | -0.62 |

Table S2.4 shows the percent of molecules with fragments from known type-1 and type-2 statins as well as percept of molecules with similarity to known statins. The NoXALL model has very low percentages for all fragments and similarities, though the T = 0.5 model does generate more type-1 fragments and has small similarities while the T = 0.0 model has zero similarities. All models with transfer-learning and the SO model have between 30-50% fragment population and similarities for type-2 fragments and statins, and about 10-15% for type-1 fragments and statins. This is in keeping with the training sets which have 50-70% fragment population and similarities for type-2 fragments and statins, and about 10-15% for type-1 fragments and statins. The T = 0.5 models tend to decrease the percentages for the type-2 statins, and increase the percentages for the type-1 statins, likely due to the fact that type-2 are over-represented in the training sets, so higher temperature allows the model to find different molecules, and type-1 are under-represented in the training sets, so the higher temperature allows the model to find those fragments.

**Table S2.4.** Percent of library molecules with a given fragment, and percent of library molecules with Tanimoto similarity to known statins of 0.25 or greater for each model at T = 0.0 and 0.5.

| | T | % Atorvastatin pharmacoph. | % Fluoro-phenyl | % butyryl | % decalin | % Similar Simvastatin | % Similar Rosuvastatin | % Si Atorva |
|---|---|---|---|---|---|---|---|---|
| **NoX1K12S** | **0.0** | 3.85 | 3.85 | 5.77 | 0.00 | 0.00 | 0.00 | 0. |

| | | | | | | | | |
|---|---|---|---|---|---|---|---|---|
| | **0.5** | 4.23 | 1.41 | 4.23 | 1.41 | 1.41 | 2.82 | 2. |
| **3X1K12S** | **0.0** | 29.17 | 35.42 | 8.33 | 8.33 | 10.42 | 27.08 | 35 |
| | **0.5** | 26.32 | 22.81 | 10.53 | 10.53 | 12.28 | 19.30 | 24 |
| **2X1K12S** | **0.0** | 35.42 | 50.00 | 4.17 | 8.33 | 8.33 | 43.75 | 43 |
| | **0.5** | 23.81 | 34.92 | 9.52 | 7.94 | 11.11 | 31.75 | 33 |
| **1X1K12S** | **0.0** | 18.75 | 31.25 | 6.25 | 6.25 | 6.25 | 25.00 | 28 |
| | **0.5** | 27.78 | 33.33 | 2.78 | 8.33 | 8.33 | 27.78 | 27 |
| **SO1K12S** | **0.0** | 36.36 | 40.91 | 9.09 | 18.18 | 18.18 | 36.36 | 40 |
| | **0.5** | 36.00 | 40.00 | 12.00 | 16.00 | 16.00 | 36.00 | 36 |
