# Supplementary figures and images for: Transformer-Decoder GPT Models for Generating Virtual Screening Libraries of HMG-Coenzyme A Reductase Inhibitors: Effects of Temperature, Prompt Length, and Transfer-Learning Strategies
Source: J Chem Inf Model. 2024 Nov 7;64(22):8464–80. doi: 10.1021/acs.jcim.4c01309 (PMC11600504; doi:10.1021/acs.jcim.4c01309)

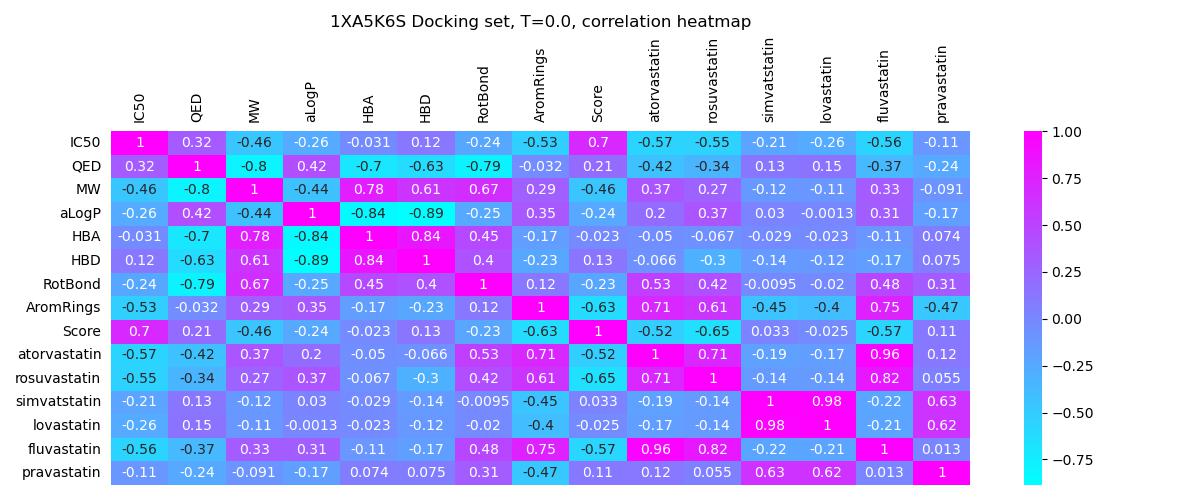

Supplement: Supplementary file 3 — ci4c01309_si_003.zip [file ci4c01309_si_003.zip › Datasets/xfer_Learning_files/1XA5K6S_model_gen_ic50_results/1XA5K6S_Docking_0p0_heatmap.jpg]

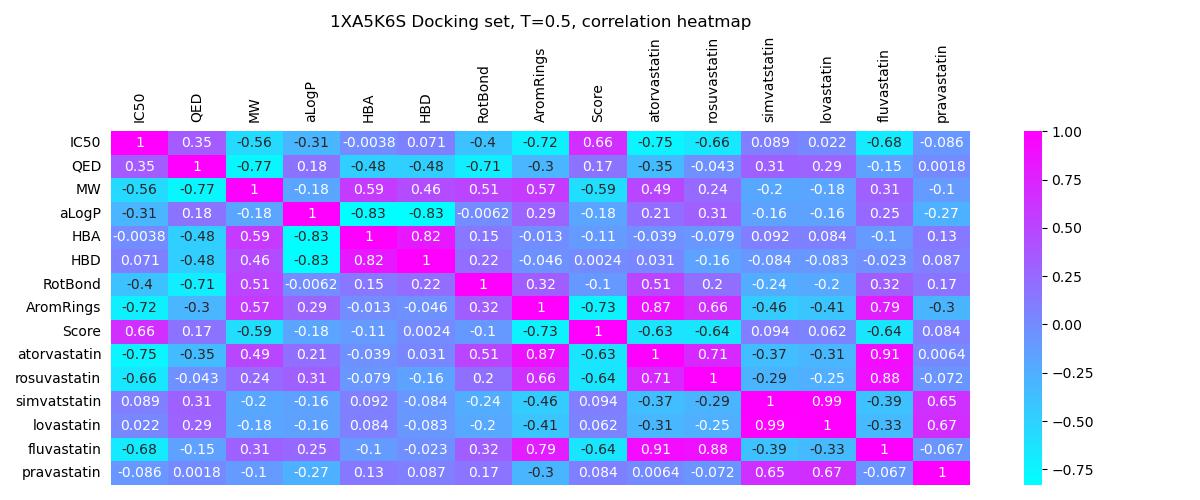

Supplement: Supplementary file 3 — ci4c01309_si_003.zip [file ci4c01309_si_003.zip › Datasets/xfer_Learning_files/1XA5K6S_model_gen_ic50_results/1XA5K6S_Docking_0p5_heatmap.jpg]

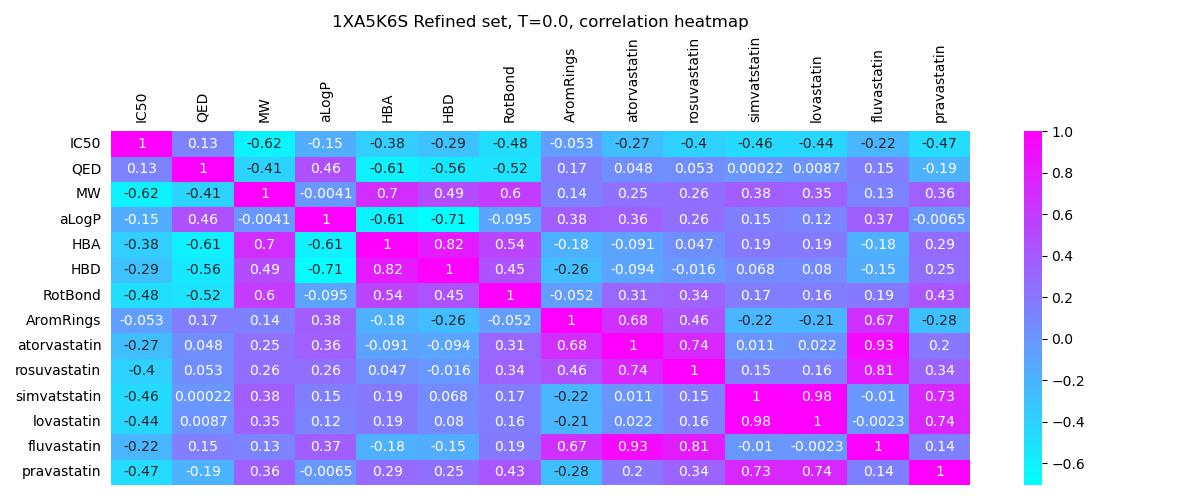

Supplement: Supplementary file 3 — ci4c01309_si_003.zip [file ci4c01309_si_003.zip › Datasets/xfer_Learning_files/1XA5K6S_model_gen_ic50_results/1XA5K6S_Refined_0p0_heatmap.jpg]

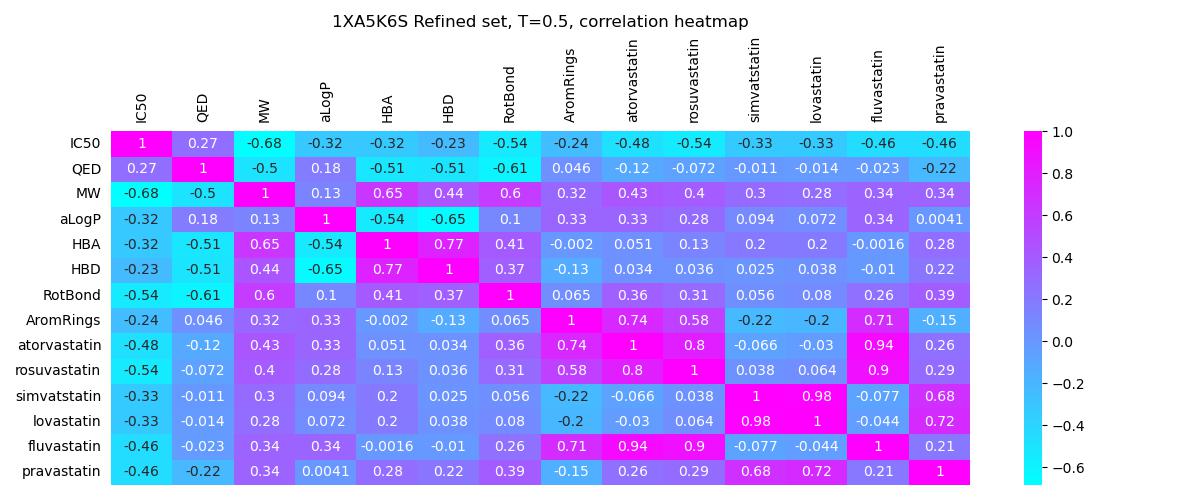

Supplement: Supplementary file 3 — ci4c01309_si_003.zip [file ci4c01309_si_003.zip › Datasets/xfer_Learning_files/1XA5K6S_model_gen_ic50_results/1XA5K6S_Refined_0p5_heatmap.jpg]

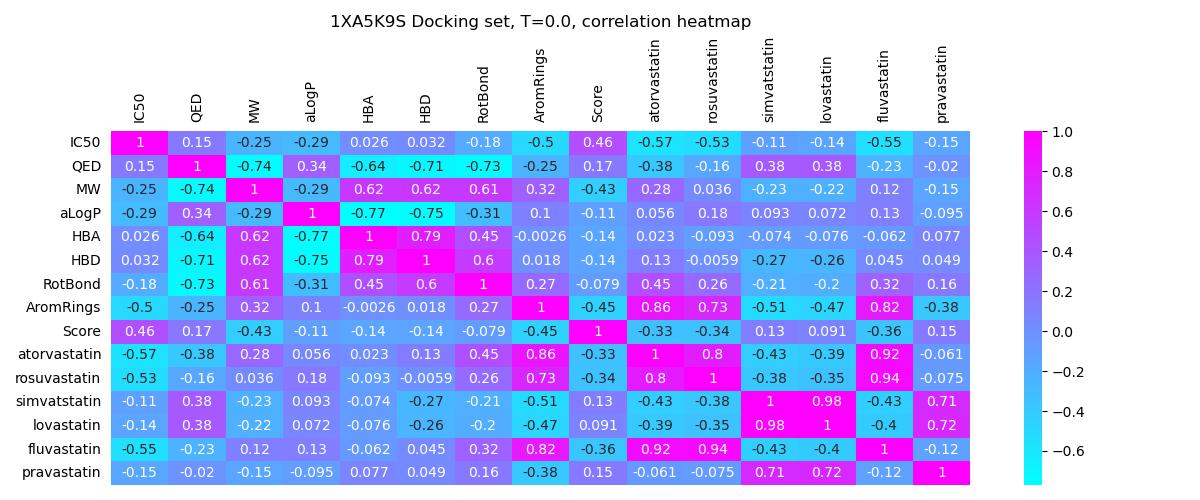

Supplement: Supplementary file 3 — ci4c01309_si_003.zip [file ci4c01309_si_003.zip › Datasets/xfer_Learning_files/1XA5K9S_model_gen_ic50_results/1XA5K9S_Docking_0p0_heatmap.jpg]

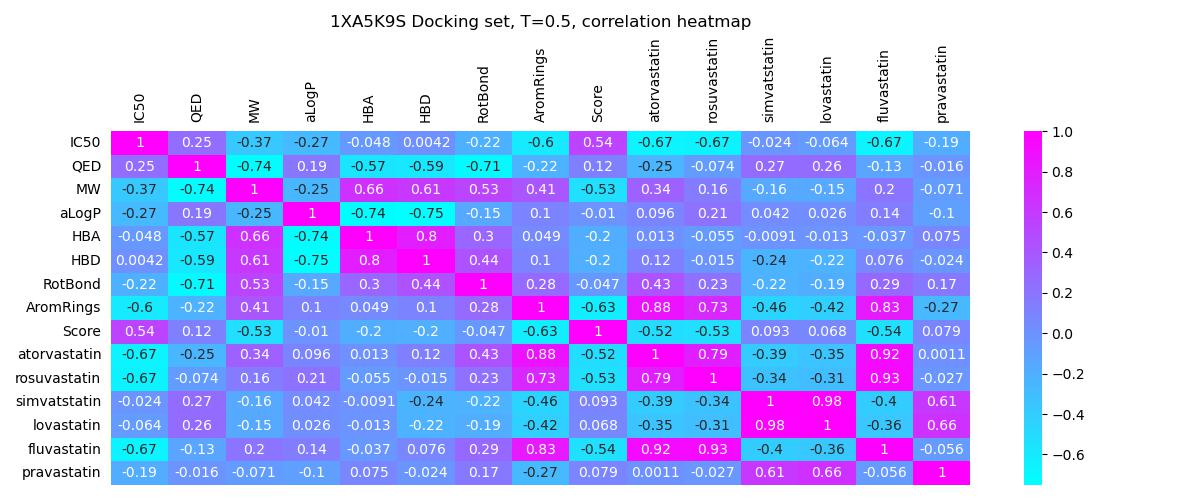

Supplement: Supplementary file 3 — ci4c01309_si_003.zip [file ci4c01309_si_003.zip › Datasets/xfer_Learning_files/1XA5K9S_model_gen_ic50_results/1XA5K9S_Docking_0p5_heatmap.jpg]

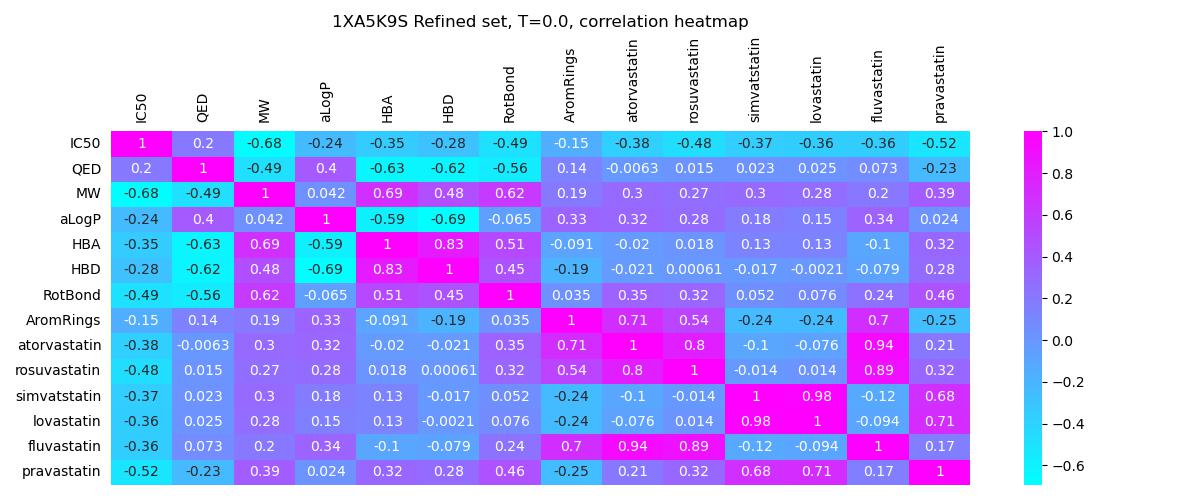

Supplement: Supplementary file 3 — ci4c01309_si_003.zip [file ci4c01309_si_003.zip › Datasets/xfer_Learning_files/1XA5K9S_model_gen_ic50_results/1XA5K9S_Refined_0p0_heatmap.jpg]

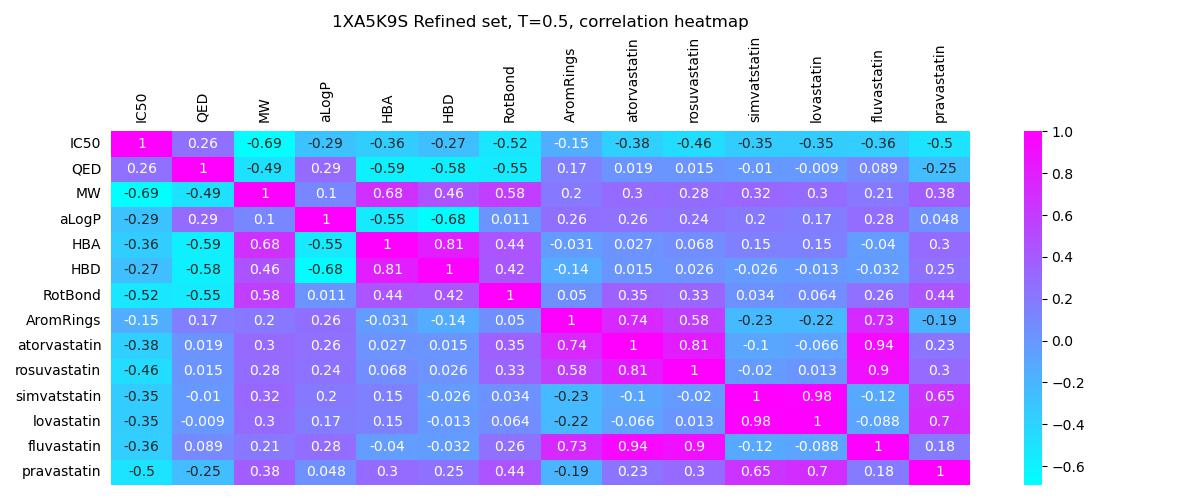

Supplement: Supplementary file 3 — ci4c01309_si_003.zip [file ci4c01309_si_003.zip › Datasets/xfer_Learning_files/1XA5K9S_model_gen_ic50_results/1XA5K9S_Refined_0p5_heatmap.jpg]

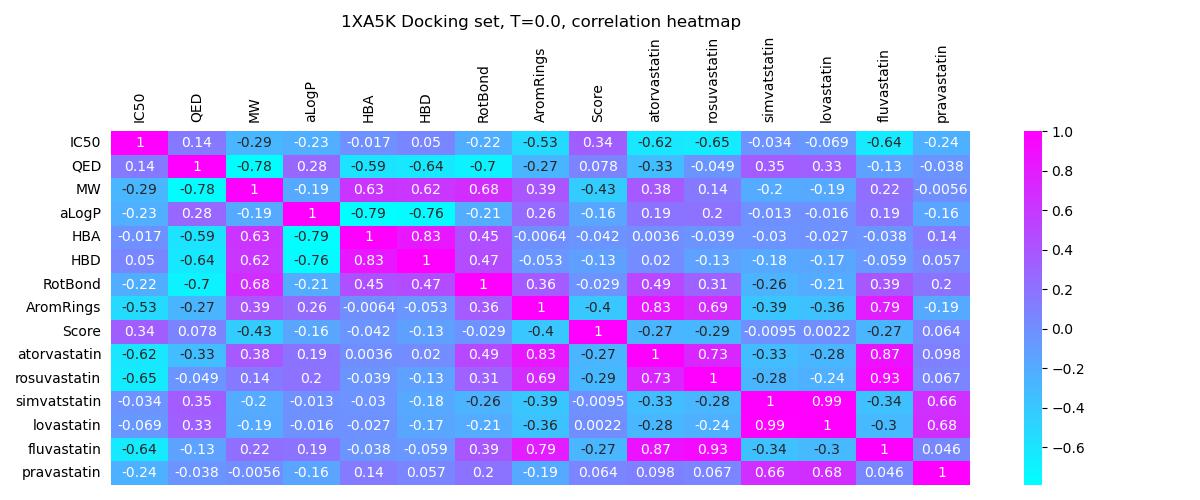

Supplement: Supplementary file 3 — ci4c01309_si_003.zip [file ci4c01309_si_003.zip › Datasets/xfer_Learning_files/1XA5K_model_gen_ic50_results/1XA5K_Docking_0p0_heatmap.jpg]

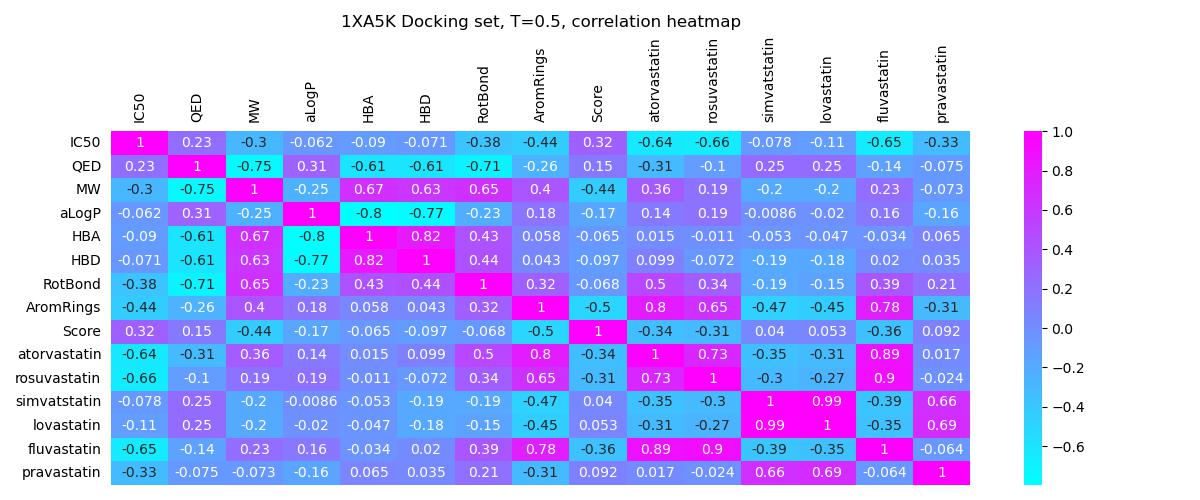

Supplement: Supplementary file 3 — ci4c01309_si_003.zip [file ci4c01309_si_003.zip › Datasets/xfer_Learning_files/1XA5K_model_gen_ic50_results/1XA5K_Docking_0p5_heatmap.jpg]

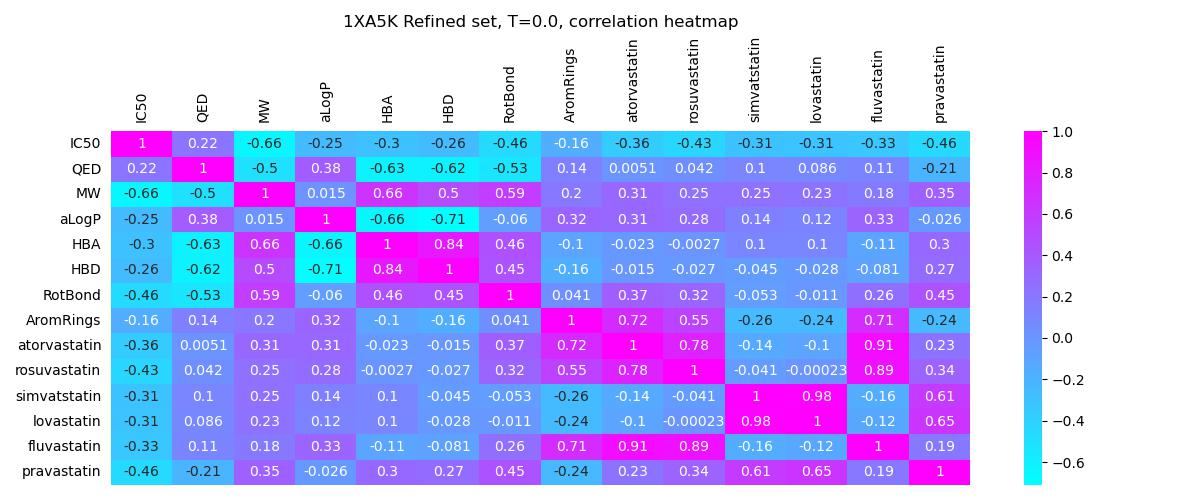

Supplement: Supplementary file 3 — ci4c01309_si_003.zip [file ci4c01309_si_003.zip › Datasets/xfer_Learning_files/1XA5K_model_gen_ic50_results/1XA5K_Refined_0p0_heatmap.jpg]

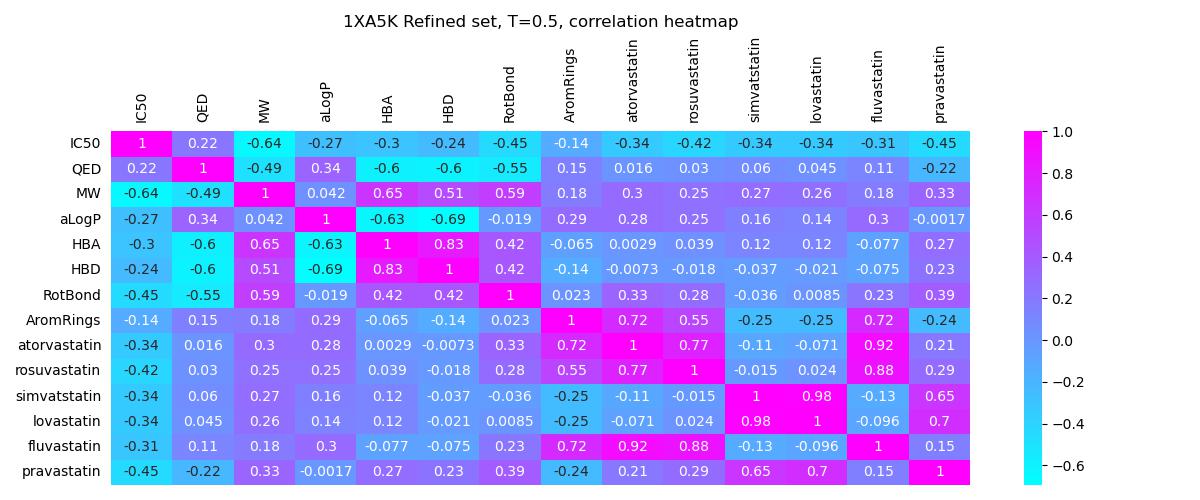

Supplement: Supplementary file 3 — ci4c01309_si_003.zip [file ci4c01309_si_003.zip › Datasets/xfer_Learning_files/1XA5K_model_gen_ic50_results/1XA5K_Refined_0p5_heatmap.jpg]

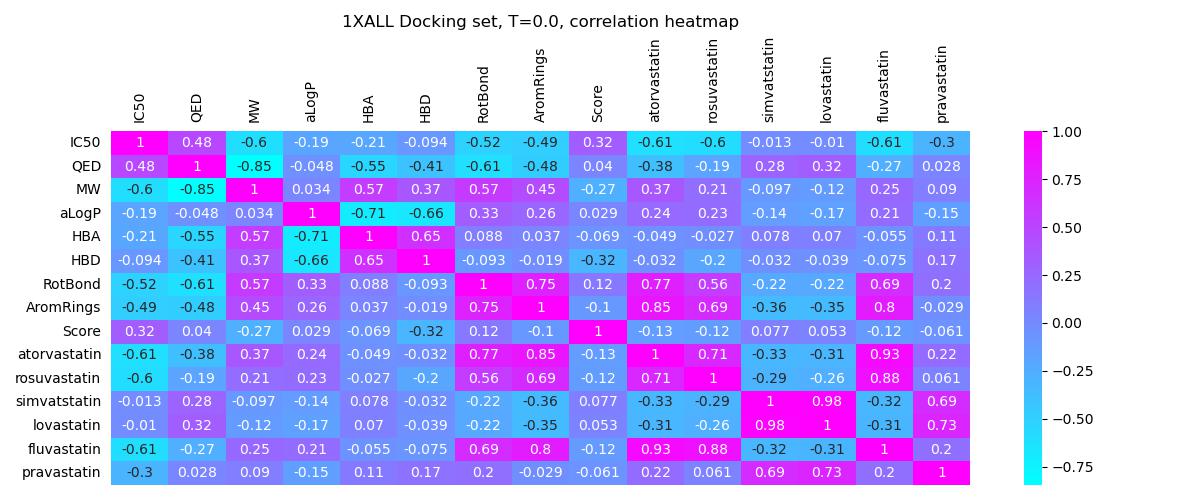

Supplement: Supplementary file 3 — ci4c01309_si_003.zip [file ci4c01309_si_003.zip › Datasets/xfer_Learning_files/1XALL_model_gen_ic50_results/1XALL_Docking_0p0_heatmap.jpg]

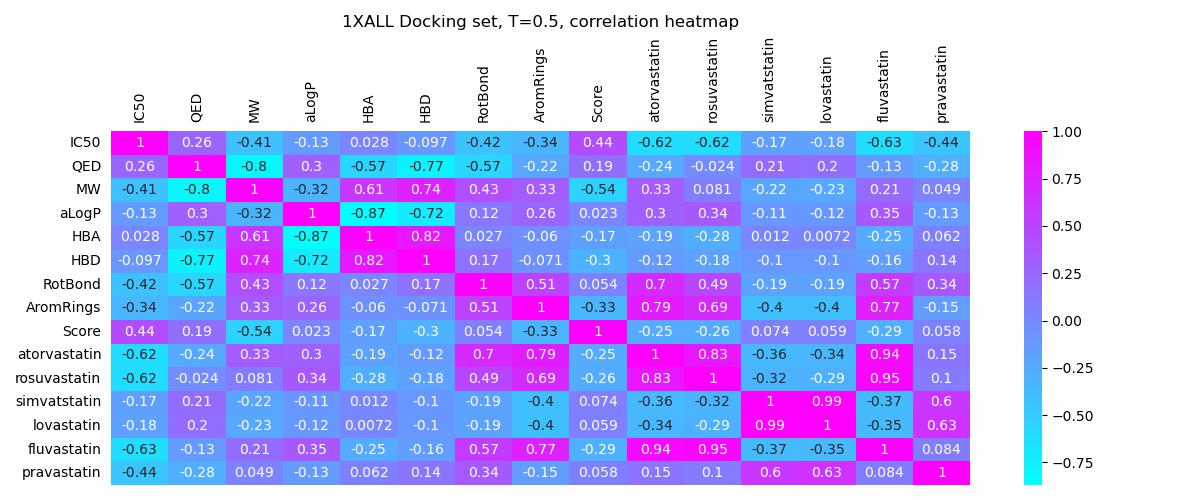

Supplement: Supplementary file 3 — ci4c01309_si_003.zip [file ci4c01309_si_003.zip › Datasets/xfer_Learning_files/1XALL_model_gen_ic50_results/1XALL_Docking_0p5_heatmap.jpg]

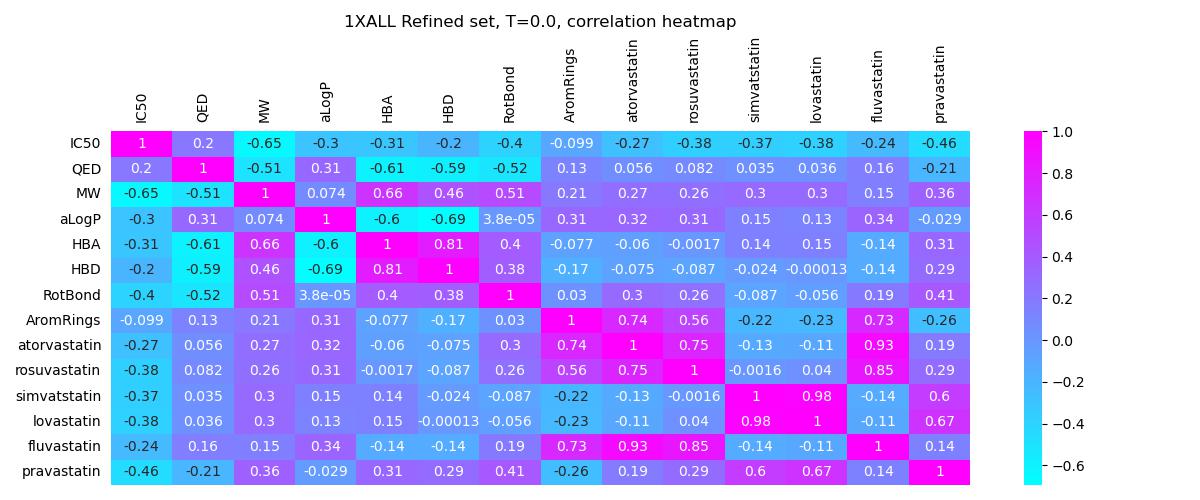

Supplement: Supplementary file 3 — ci4c01309_si_003.zip [file ci4c01309_si_003.zip › Datasets/xfer_Learning_files/1XALL_model_gen_ic50_results/1XALL_Refined_0p0_heatmap.jpg]

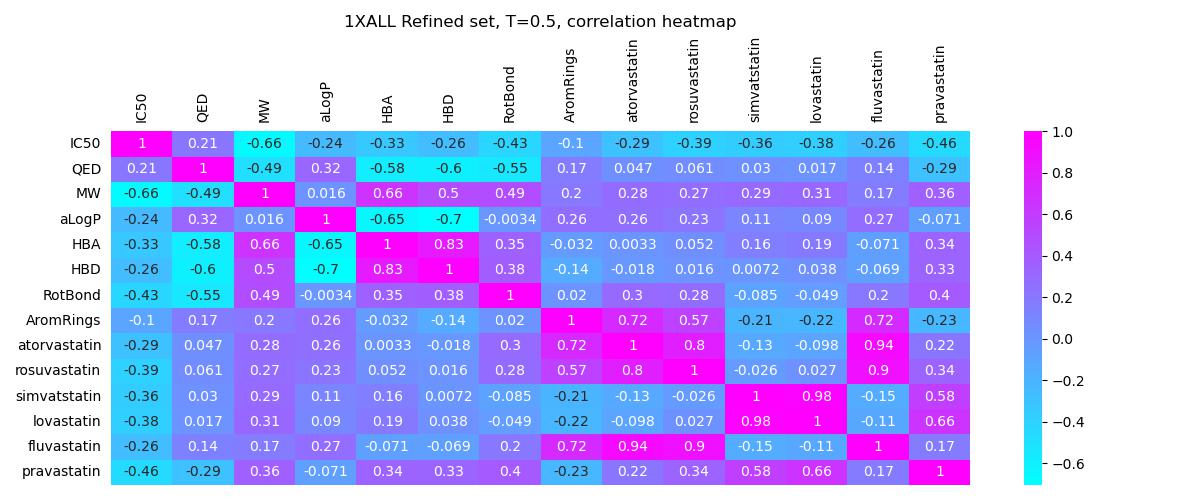

Supplement: Supplementary file 3 — ci4c01309_si_003.zip [file ci4c01309_si_003.zip › Datasets/xfer_Learning_files/1XALL_model_gen_ic50_results/1XALL_Refined_0p5_heatmap.jpg]

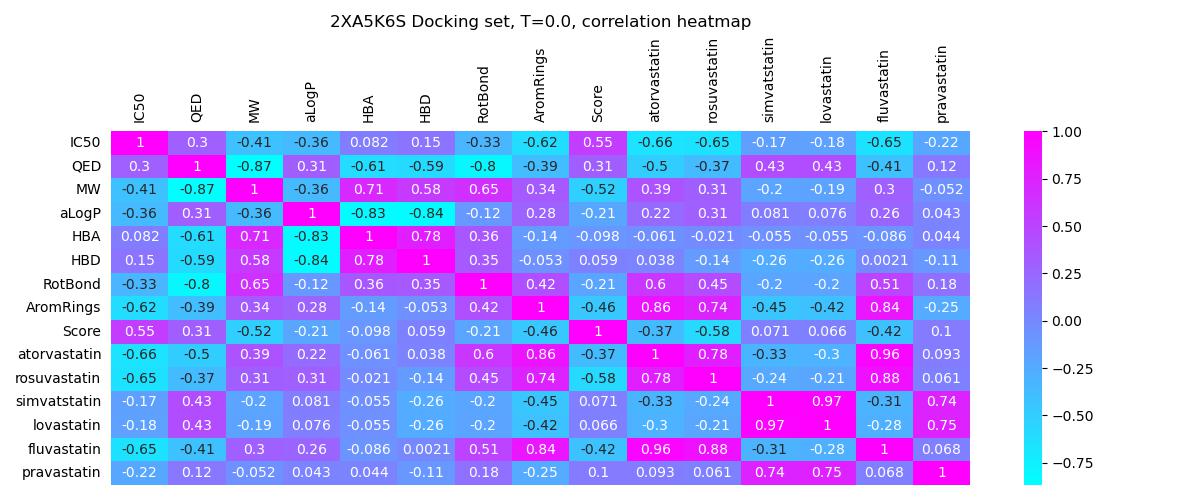

Supplement: Supplementary file 3 — ci4c01309_si_003.zip [file ci4c01309_si_003.zip › Datasets/xfer_Learning_files/2XA5K6S_model_gen_ic50_results/2XA5K6S_Docking_0p0_heatmap.jpg]

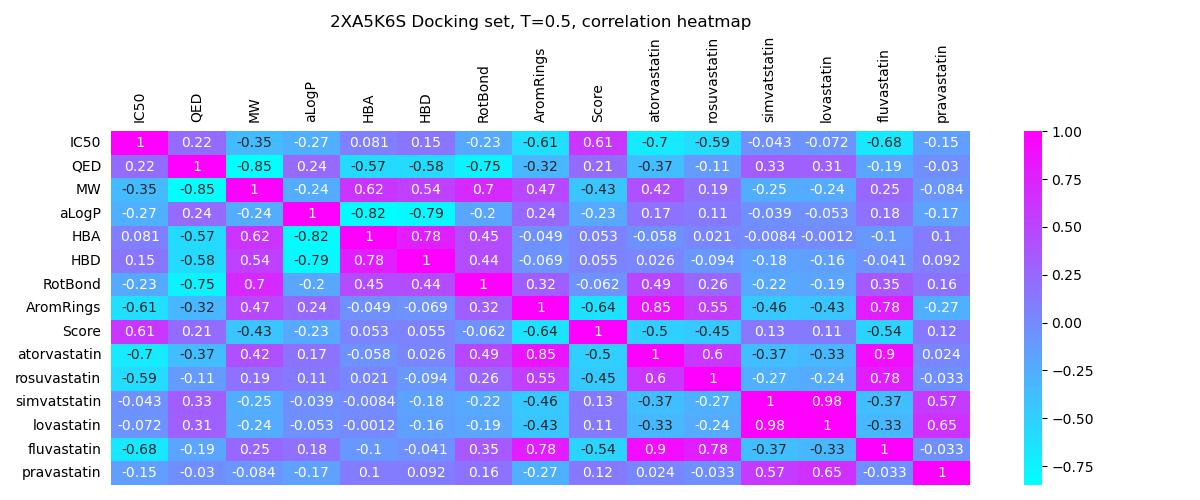

Supplement: Supplementary file 3 — ci4c01309_si_003.zip [file ci4c01309_si_003.zip › Datasets/xfer_Learning_files/2XA5K6S_model_gen_ic50_results/2XA5K6S_Docking_0p5_heatmap.jpg]

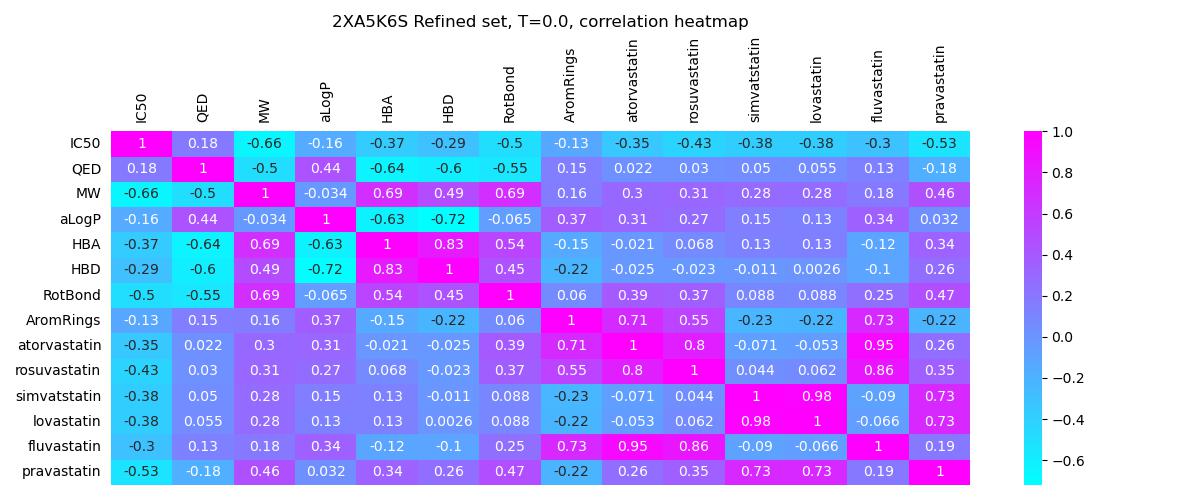

Supplement: Supplementary file 3 — ci4c01309_si_003.zip [file ci4c01309_si_003.zip › Datasets/xfer_Learning_files/2XA5K6S_model_gen_ic50_results/2XA5K6S_Refined_0p0_heatmap.jpg]

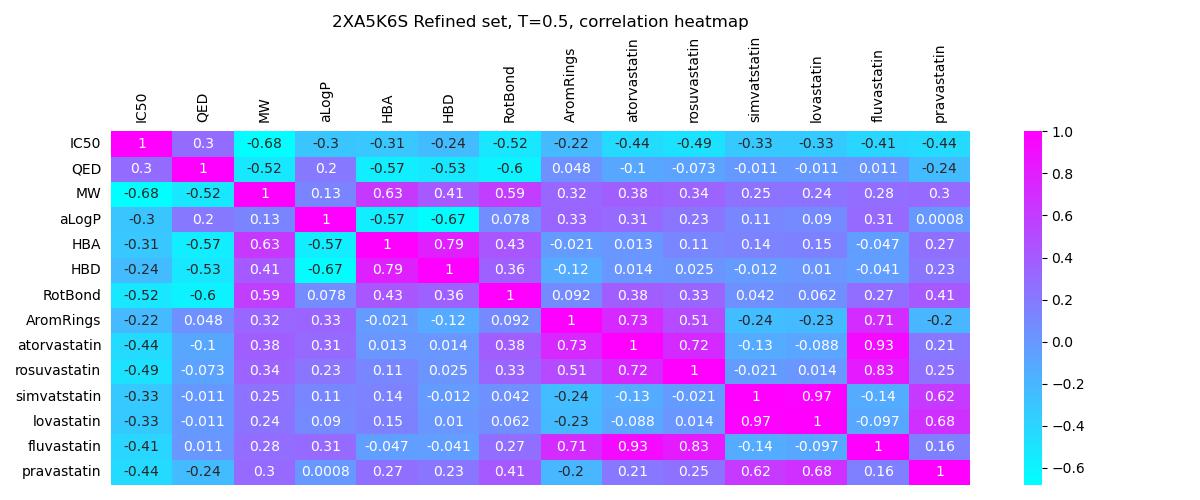

Supplement: Supplementary file 3 — ci4c01309_si_003.zip [file ci4c01309_si_003.zip › Datasets/xfer_Learning_files/2XA5K6S_model_gen_ic50_results/2XA5K6S_Refined_0p5_heatmap.jpg]

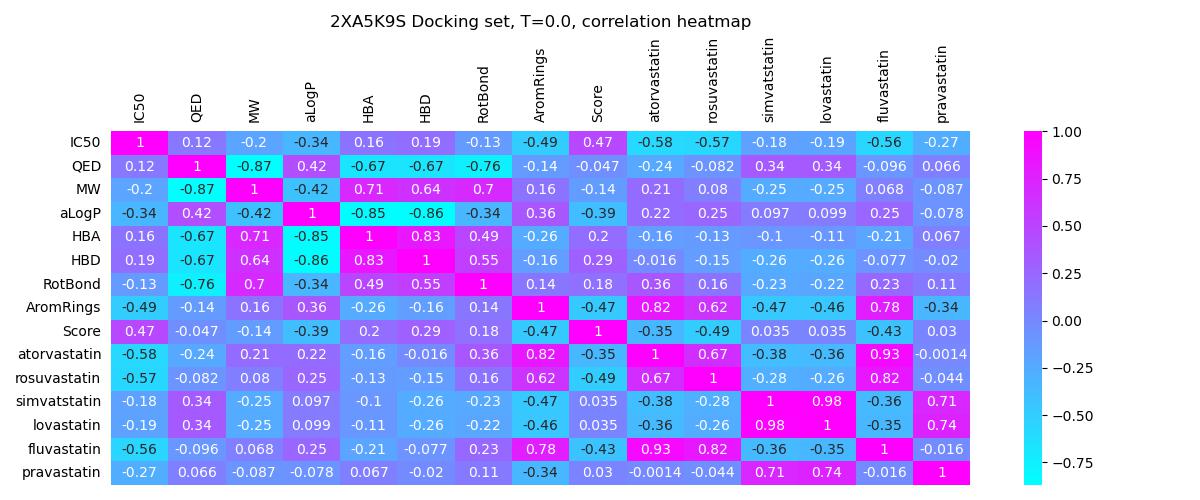

Supplement: Supplementary file 3 — ci4c01309_si_003.zip [file ci4c01309_si_003.zip › Datasets/xfer_Learning_files/2XA5K9S_model_gen_ic50_results/2XA5K9S_Docking_0p0_heatmap.jpg]

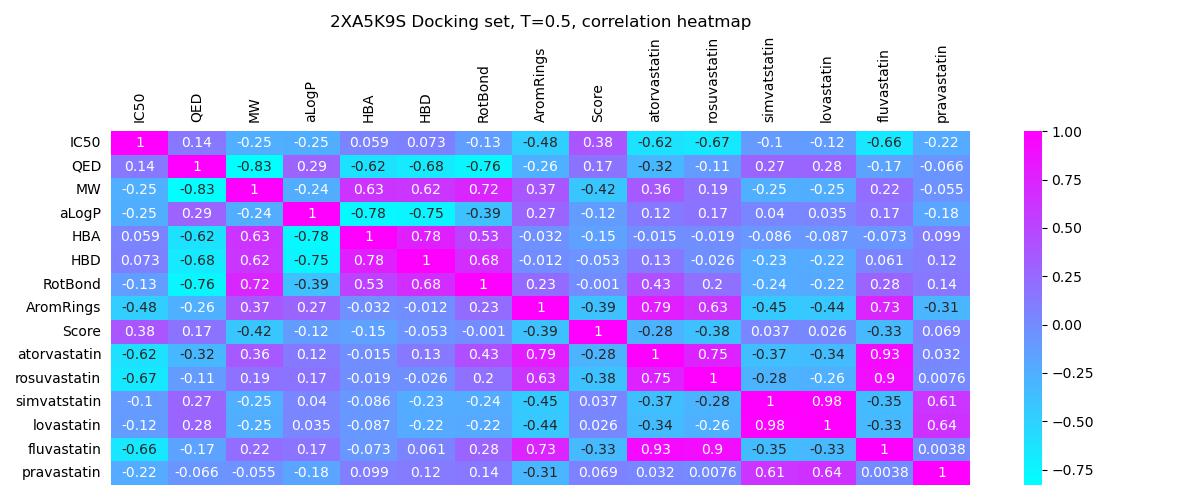

Supplement: Supplementary file 3 — ci4c01309_si_003.zip [file ci4c01309_si_003.zip › Datasets/xfer_Learning_files/2XA5K9S_model_gen_ic50_results/2XA5K9S_Docking_0p5_heatmap.jpg]

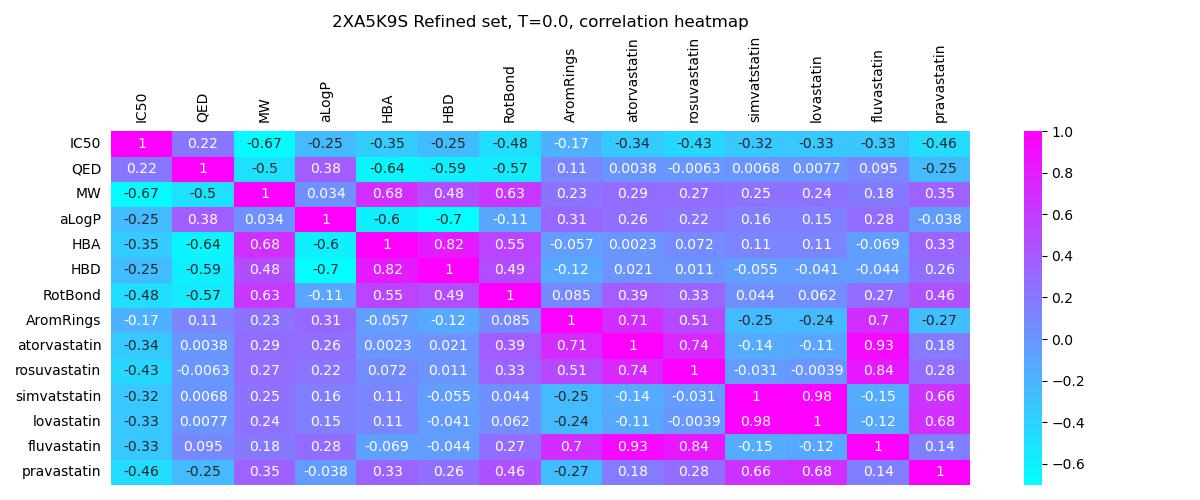

Supplement: Supplementary file 3 — ci4c01309_si_003.zip [file ci4c01309_si_003.zip › Datasets/xfer_Learning_files/2XA5K9S_model_gen_ic50_results/2XA5K9S_Refined_0p0_heatmap.jpg]

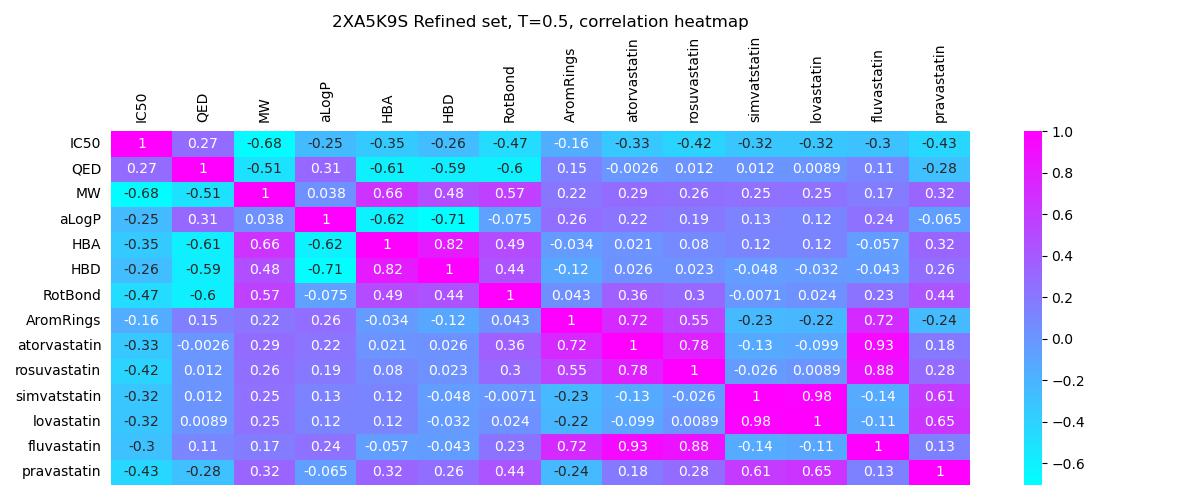

Supplement: Supplementary file 3 — ci4c01309_si_003.zip [file ci4c01309_si_003.zip › Datasets/xfer_Learning_files/2XA5K9S_model_gen_ic50_results/2XA5K9S_Refined_0p5_heatmap.jpg]

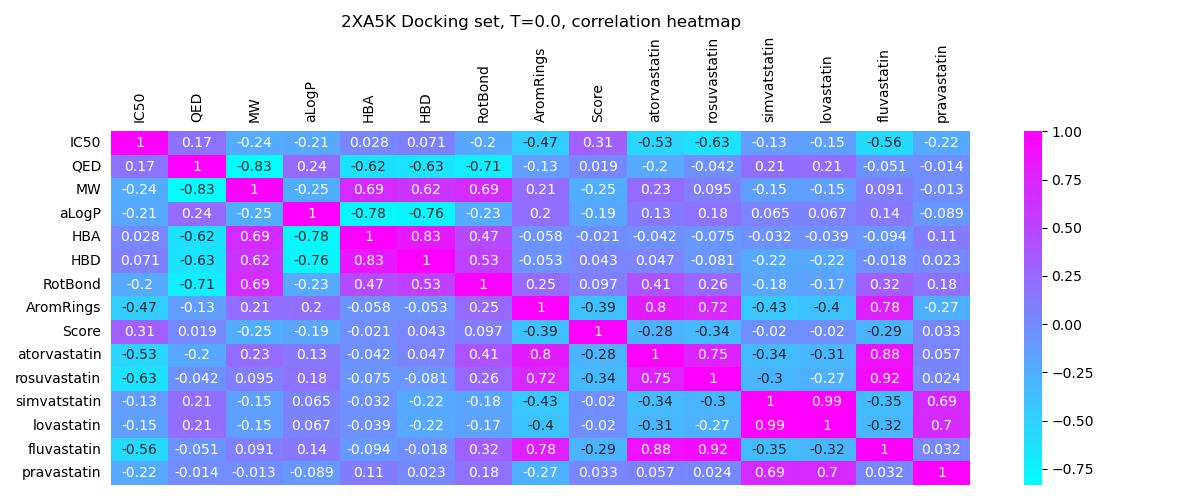

Supplement: Supplementary file 3 — ci4c01309_si_003.zip [file ci4c01309_si_003.zip › Datasets/xfer_Learning_files/2XA5K_model_gen_ic50_results/2XA5K_Docking_0p0_heatmap.jpg]

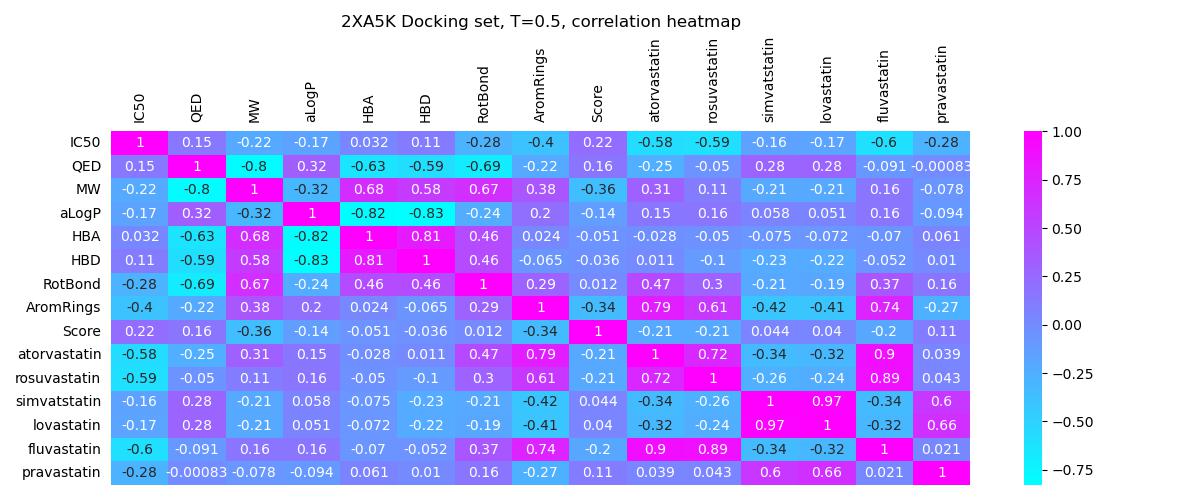

Supplement: Supplementary file 3 — ci4c01309_si_003.zip [file ci4c01309_si_003.zip › Datasets/xfer_Learning_files/2XA5K_model_gen_ic50_results/2XA5K_Docking_0p5_heatmap.jpg]

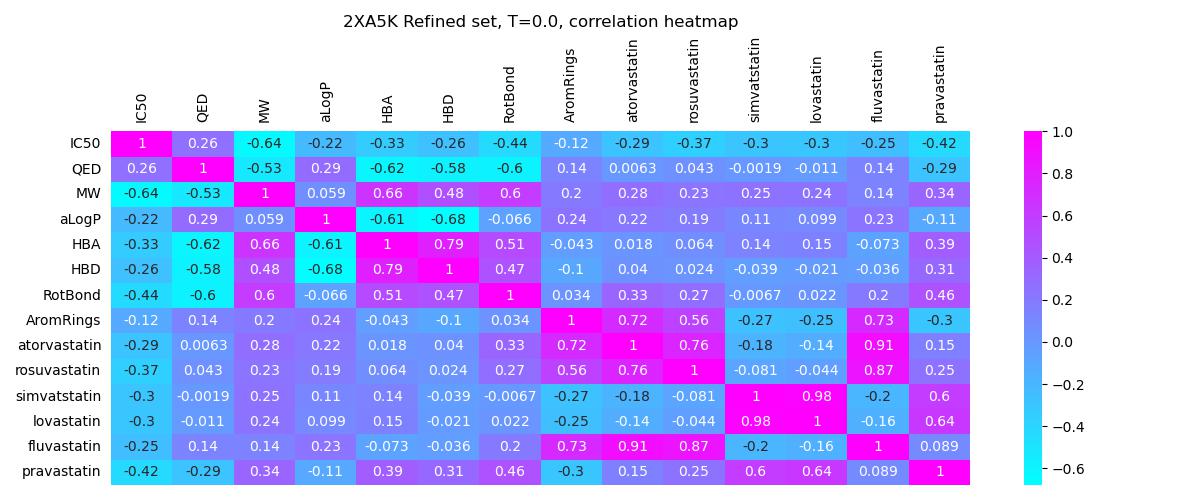

Supplement: Supplementary file 3 — ci4c01309_si_003.zip [file ci4c01309_si_003.zip › Datasets/xfer_Learning_files/2XA5K_model_gen_ic50_results/2XA5K_Refined_0p0_heatmap.jpg]

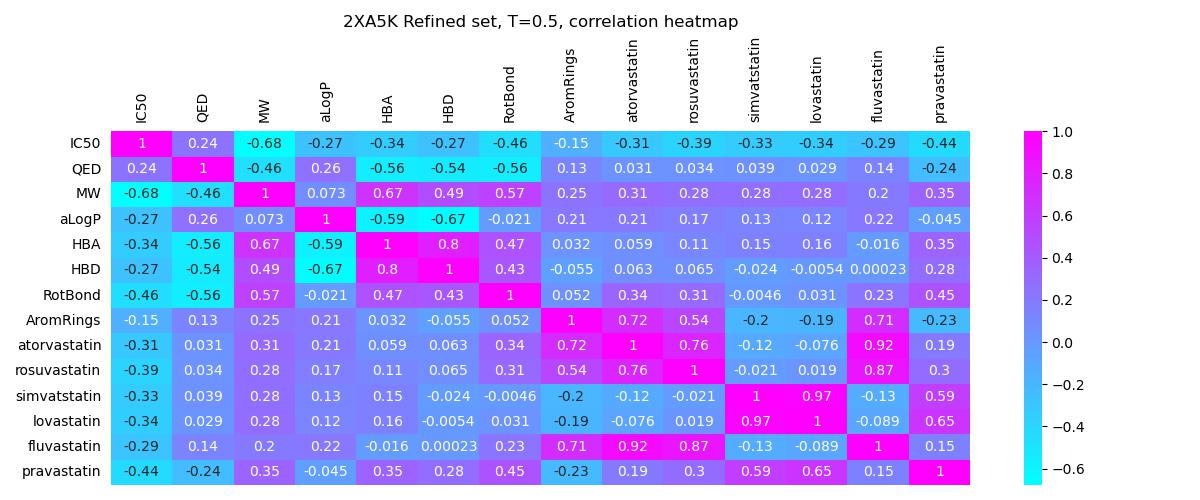

Supplement: Supplementary file 3 — ci4c01309_si_003.zip [file ci4c01309_si_003.zip › Datasets/xfer_Learning_files/2XA5K_model_gen_ic50_results/2XA5K_Refined_0p5_heatmap.jpg]

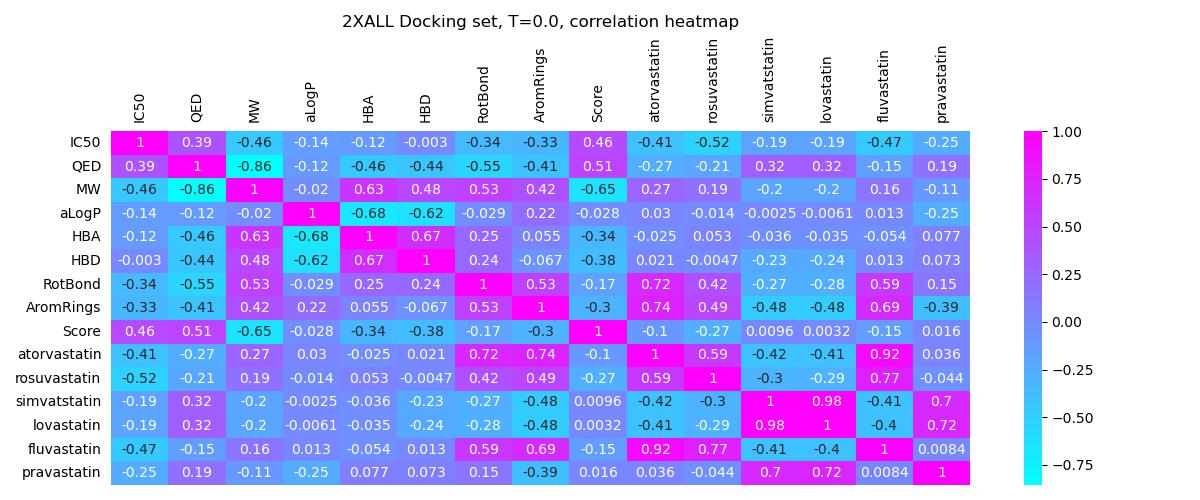

Supplement: Supplementary file 3 — ci4c01309_si_003.zip [file ci4c01309_si_003.zip › Datasets/xfer_Learning_files/2XALL_model_gen_ic50_results/2XALL_Docking_0p0_heatmap.jpg]

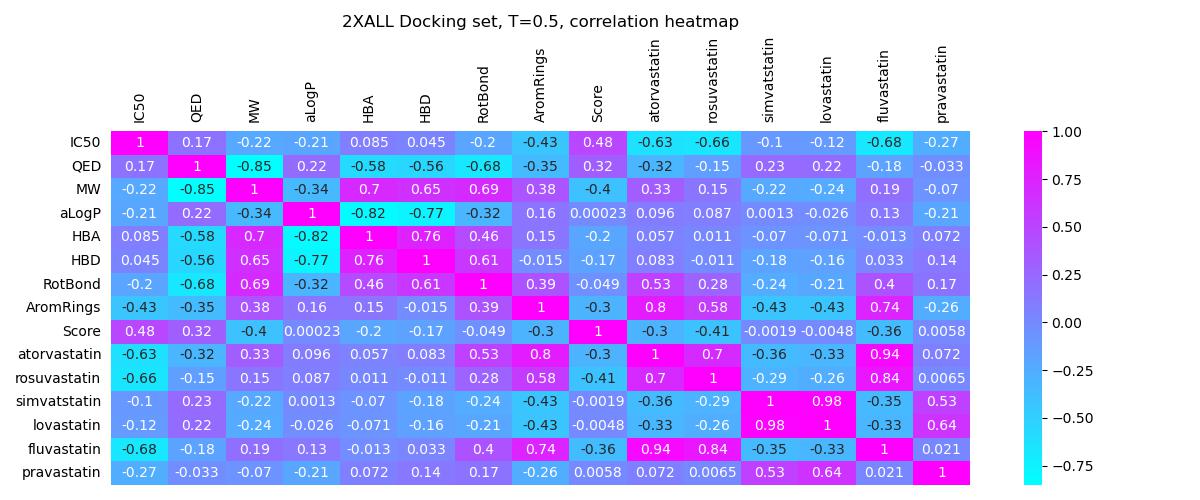

Supplement: Supplementary file 3 — ci4c01309_si_003.zip [file ci4c01309_si_003.zip › Datasets/xfer_Learning_files/2XALL_model_gen_ic50_results/2XALL_Docking_0p5_heatmap.jpg]

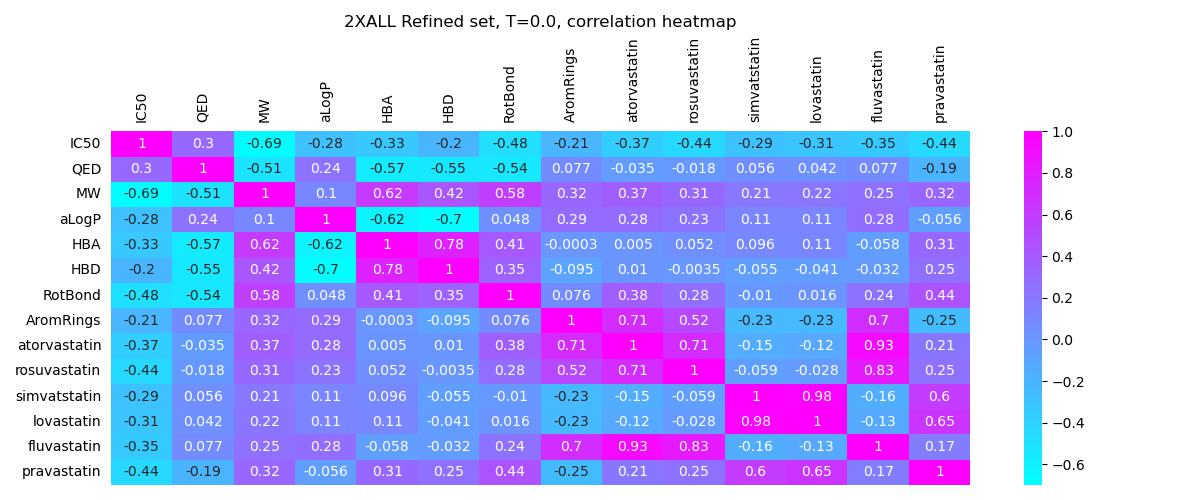

Supplement: Supplementary file 3 — ci4c01309_si_003.zip [file ci4c01309_si_003.zip › Datasets/xfer_Learning_files/2XALL_model_gen_ic50_results/2XALL_Refined_0p0_heatmap.jpg]

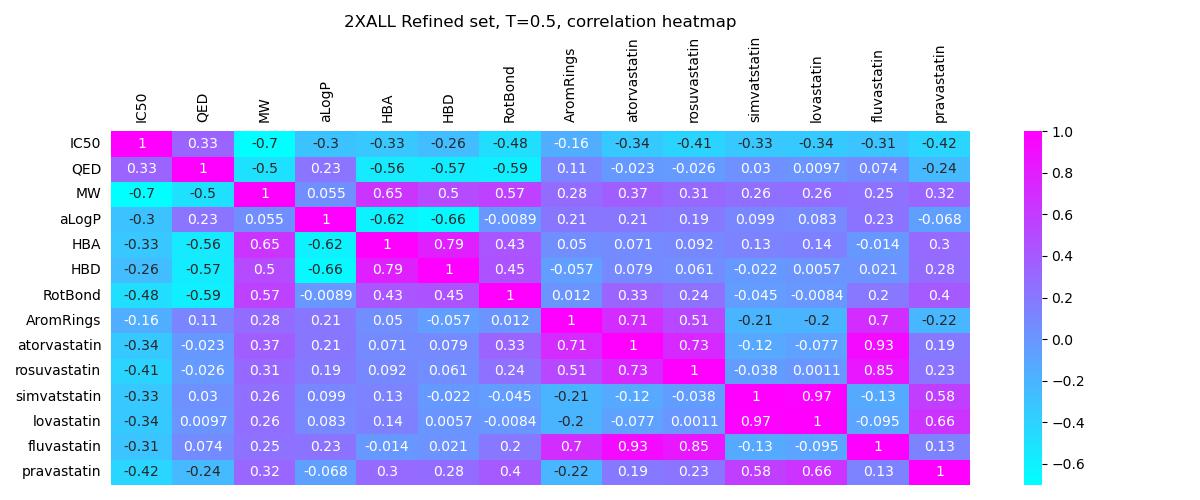

Supplement: Supplementary file 3 — ci4c01309_si_003.zip [file ci4c01309_si_003.zip › Datasets/xfer_Learning_files/2XALL_model_gen_ic50_results/2XALL_Refined_0p5_heatmap.jpg]

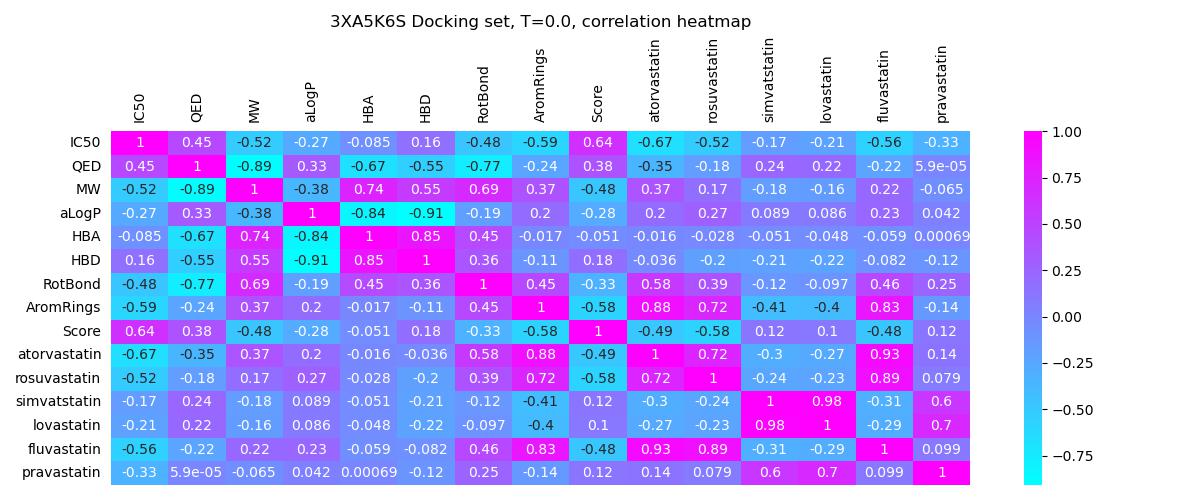

Supplement: Supplementary file 3 — ci4c01309_si_003.zip [file ci4c01309_si_003.zip › Datasets/xfer_Learning_files/3XA5K6S_model_gen_ic50_results/3XA5K6S_Docking_0p0_heatmap.jpg]

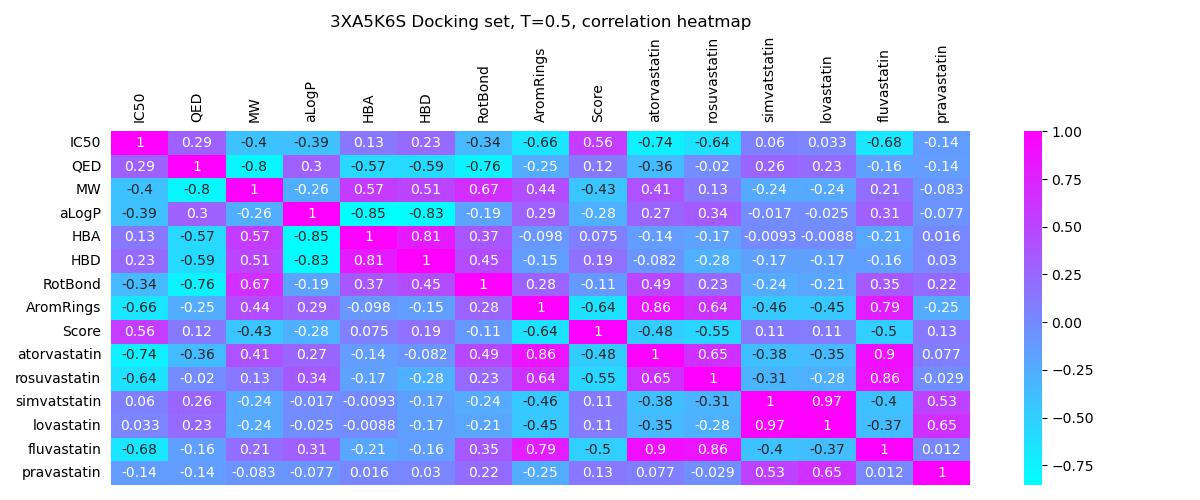

Supplement: Supplementary file 3 — ci4c01309_si_003.zip [file ci4c01309_si_003.zip › Datasets/xfer_Learning_files/3XA5K6S_model_gen_ic50_results/3XA5K6S_Docking_0p5_heatmap.jpg]

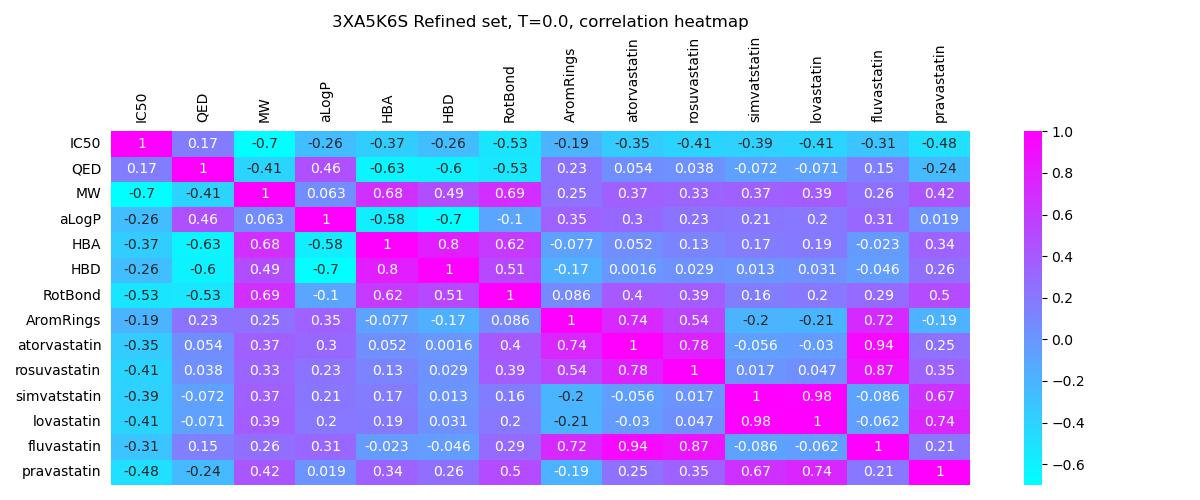

Supplement: Supplementary file 3 — ci4c01309_si_003.zip [file ci4c01309_si_003.zip › Datasets/xfer_Learning_files/3XA5K6S_model_gen_ic50_results/3XA5K6S_Refined_0p0_heatmap.jpg]

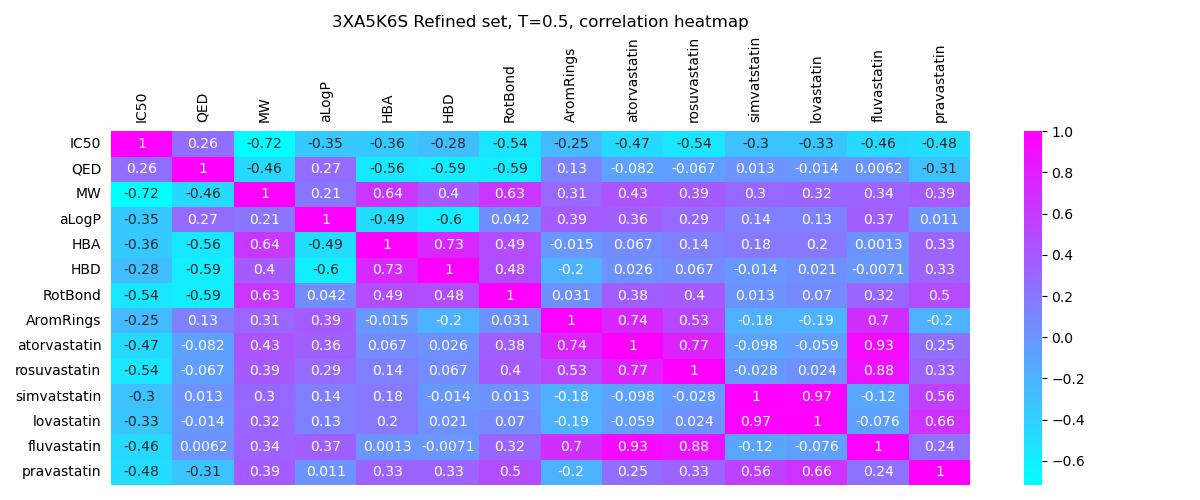

Supplement: Supplementary file 3 — ci4c01309_si_003.zip [file ci4c01309_si_003.zip › Datasets/xfer_Learning_files/3XA5K6S_model_gen_ic50_results/3XA5K6S_Refined_0p5_heatmap.jpg]

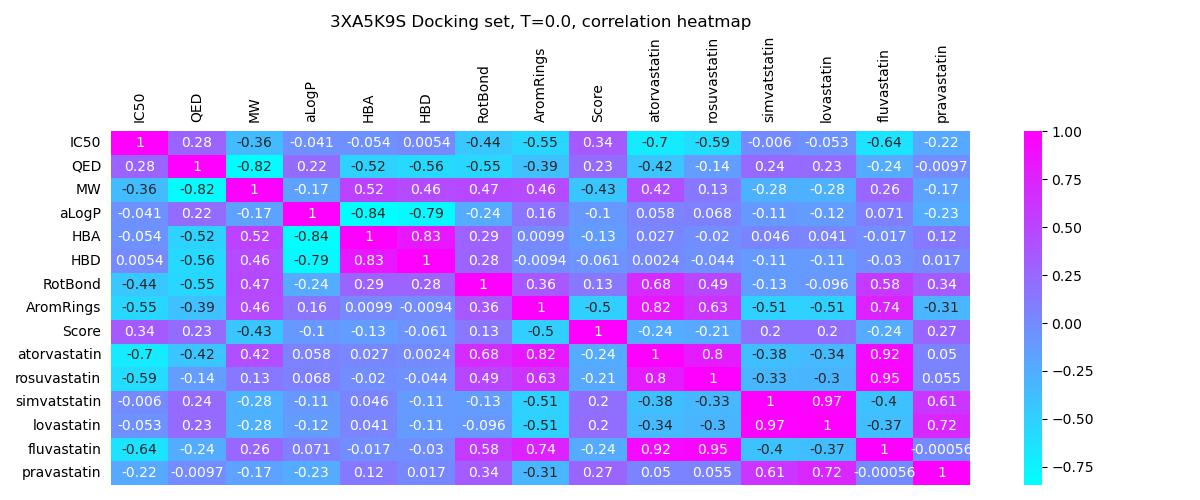

Supplement: Supplementary file 3 — ci4c01309_si_003.zip [file ci4c01309_si_003.zip › Datasets/xfer_Learning_files/3XA5K9S_model_gen_ic50_results/3XA5K9S_Docking_0p0_heatmap.jpg]

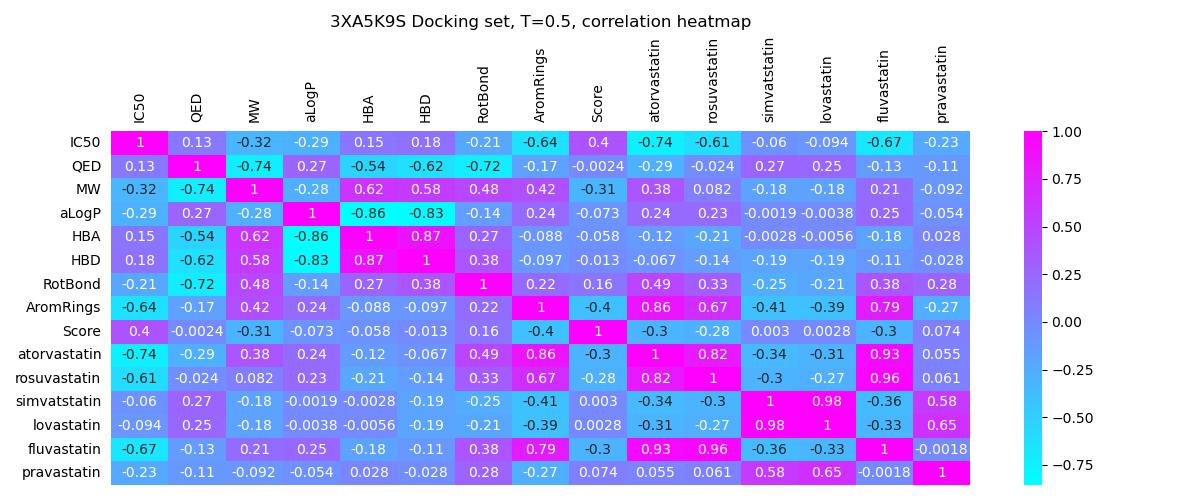

Supplement: Supplementary file 3 — ci4c01309_si_003.zip [file ci4c01309_si_003.zip › Datasets/xfer_Learning_files/3XA5K9S_model_gen_ic50_results/3XA5K9S_Docking_0p5_heatmap.jpg]

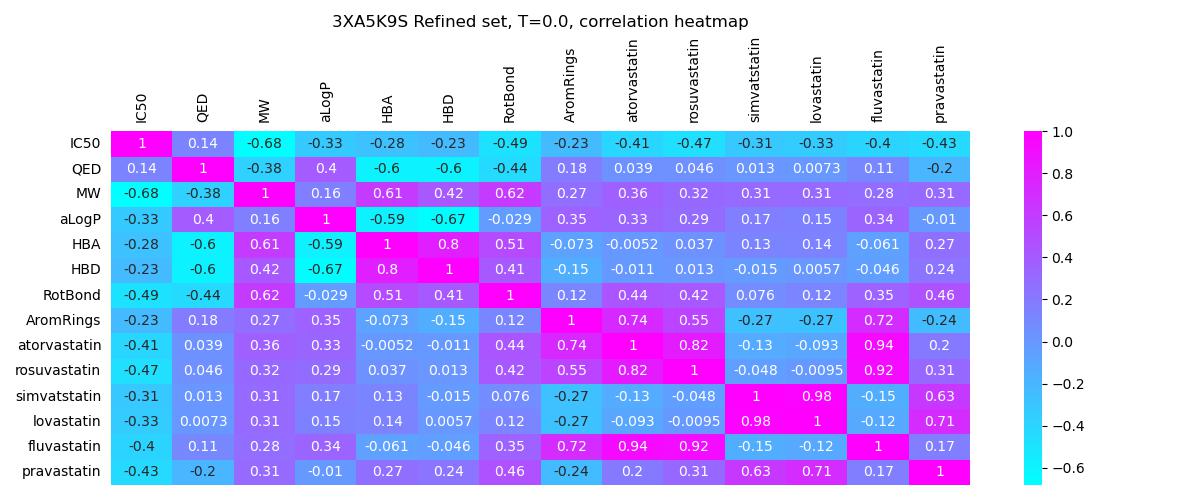

Supplement: Supplementary file 3 — ci4c01309_si_003.zip [file ci4c01309_si_003.zip › Datasets/xfer_Learning_files/3XA5K9S_model_gen_ic50_results/3XA5K9S_Refined_0p0_heatmap.jpg]

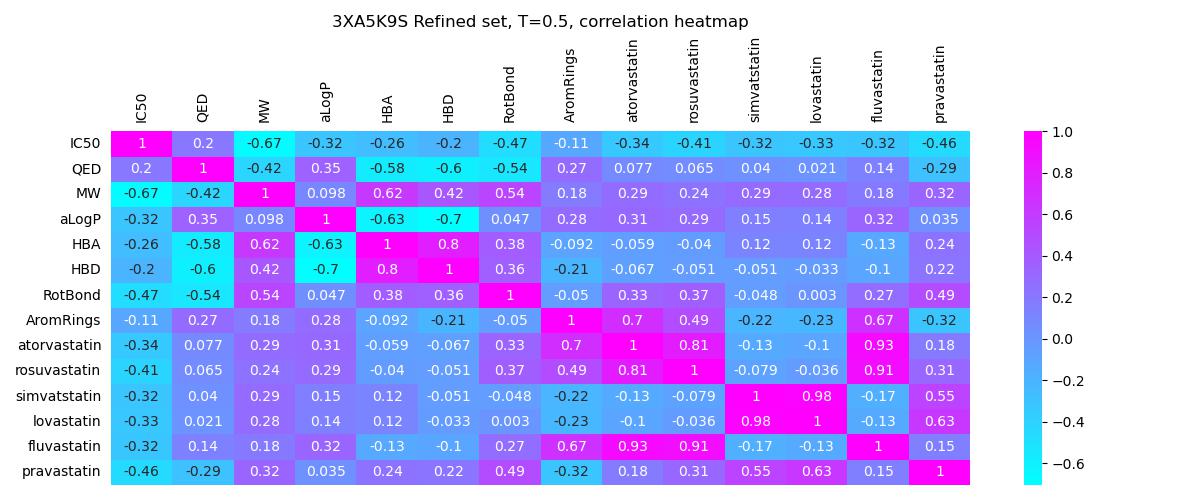

Supplement: Supplementary file 3 — ci4c01309_si_003.zip [file ci4c01309_si_003.zip › Datasets/xfer_Learning_files/3XA5K9S_model_gen_ic50_results/3XA5K9S_Refined_0p5_heatmap.jpg]

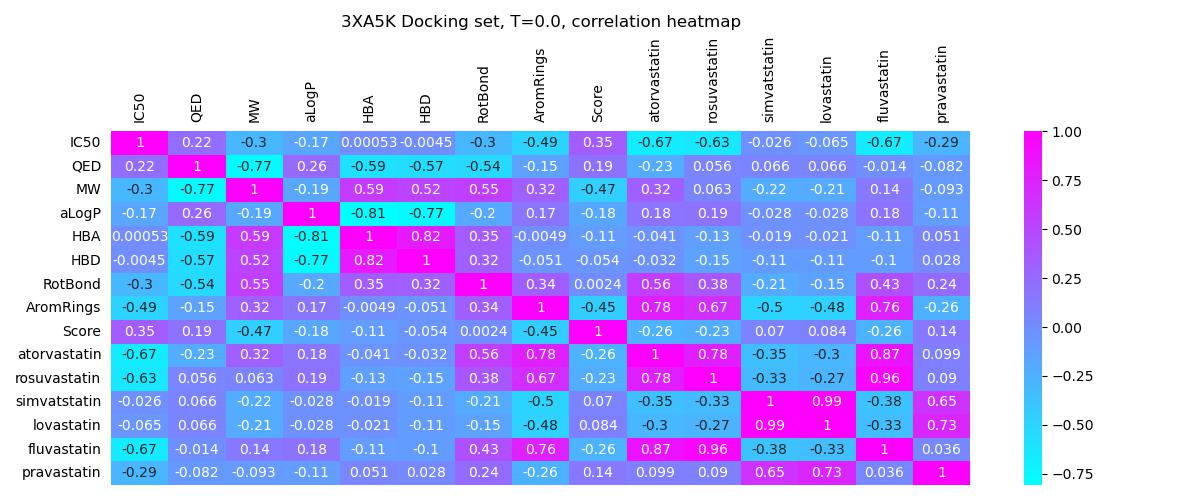

Supplement: Supplementary file 3 — ci4c01309_si_003.zip [file ci4c01309_si_003.zip › Datasets/xfer_Learning_files/3XA5K_model_gen_ic50_results/3XA5K_Docking_0p0_heatmap.jpg]

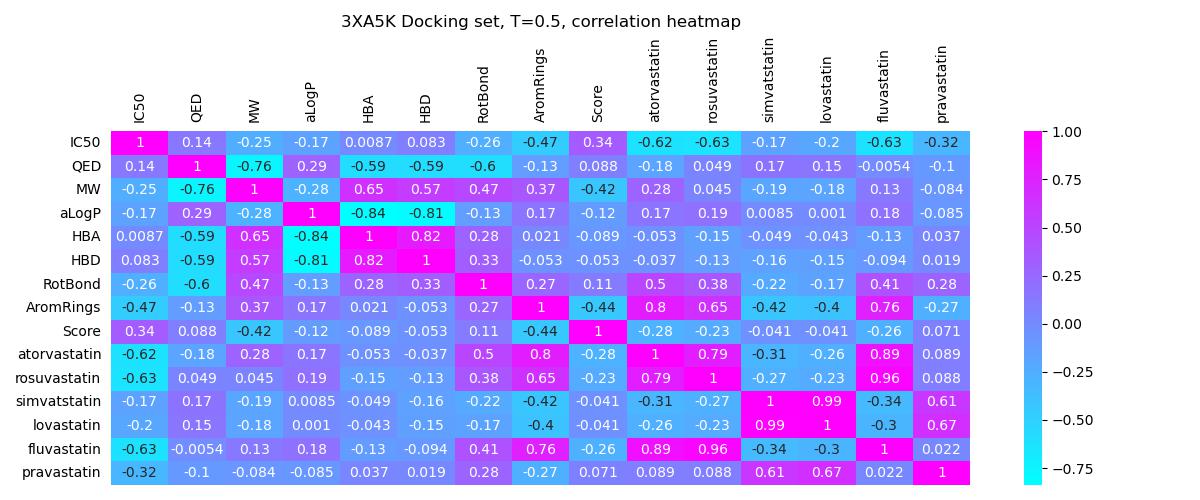

Supplement: Supplementary file 3 — ci4c01309_si_003.zip [file ci4c01309_si_003.zip › Datasets/xfer_Learning_files/3XA5K_model_gen_ic50_results/3XA5K_Docking_0p5_heatmap.jpg]

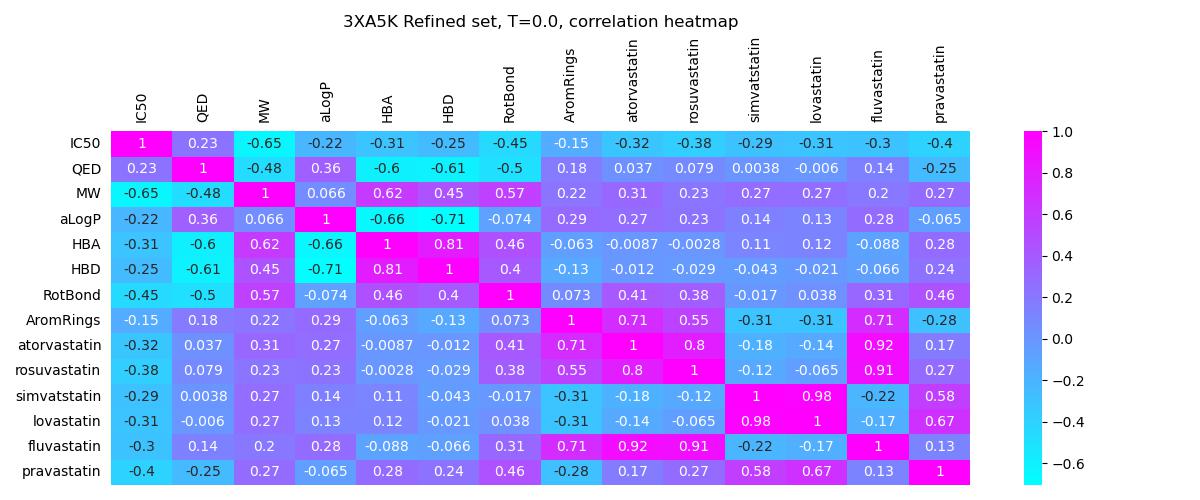

Supplement: Supplementary file 3 — ci4c01309_si_003.zip [file ci4c01309_si_003.zip › Datasets/xfer_Learning_files/3XA5K_model_gen_ic50_results/3XA5K_Refined_0p0_heatmap.jpg]

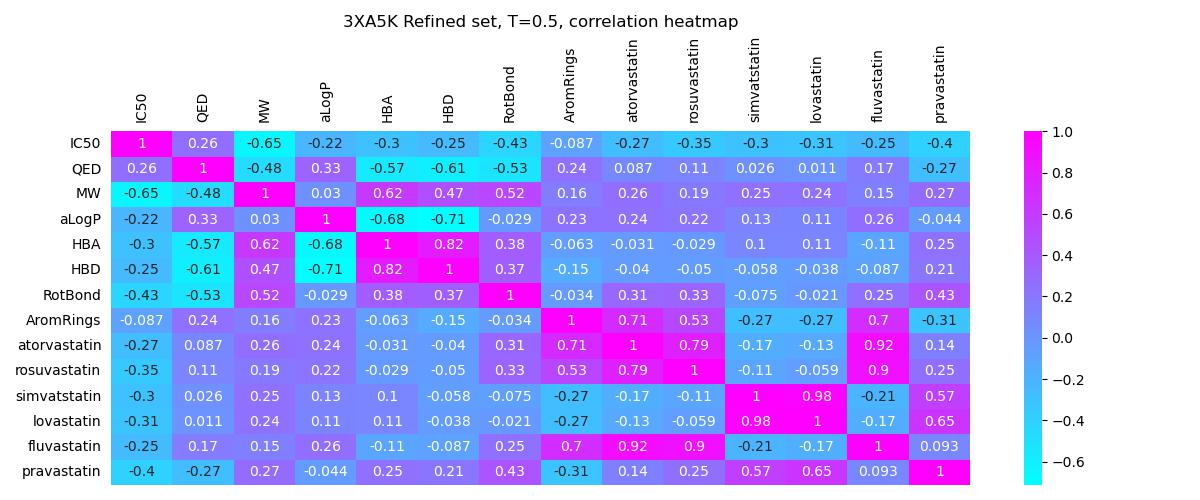

Supplement: Supplementary file 3 — ci4c01309_si_003.zip [file ci4c01309_si_003.zip › Datasets/xfer_Learning_files/3XA5K_model_gen_ic50_results/3XA5K_Refined_0p5_heatmap.jpg]

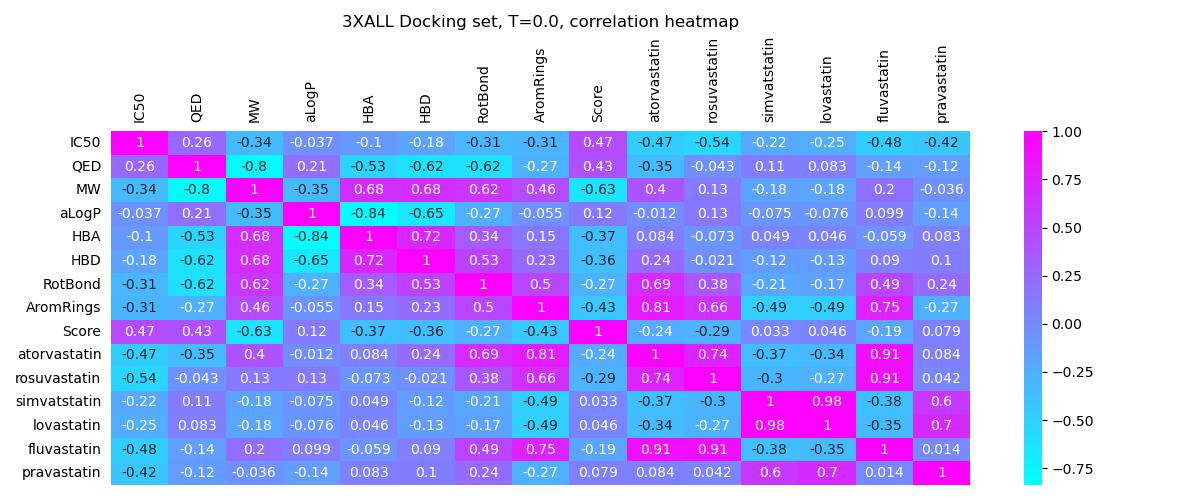

Supplement: Supplementary file 3 — ci4c01309_si_003.zip [file ci4c01309_si_003.zip › Datasets/xfer_Learning_files/3XALL_model_gen_ic50_results/3XALL_Docking_0p0_heatmap.jpg]

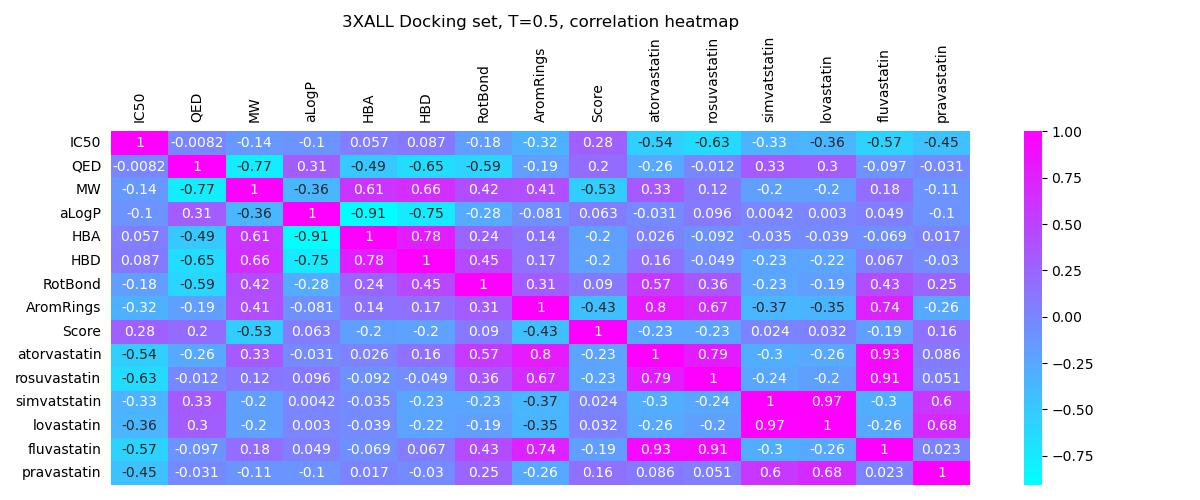

Supplement: Supplementary file 3 — ci4c01309_si_003.zip [file ci4c01309_si_003.zip › Datasets/xfer_Learning_files/3XALL_model_gen_ic50_results/3XALL_Docking_0p5_heatmap.jpg]

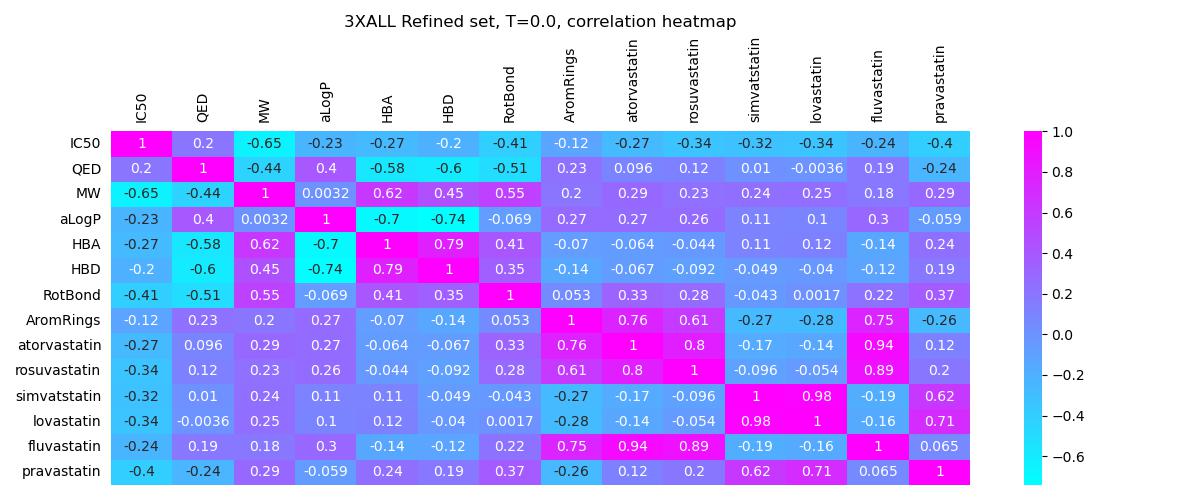

Supplement: Supplementary file 3 — ci4c01309_si_003.zip [file ci4c01309_si_003.zip › Datasets/xfer_Learning_files/3XALL_model_gen_ic50_results/3XALL_Refined_0p0_heatmap.jpg]

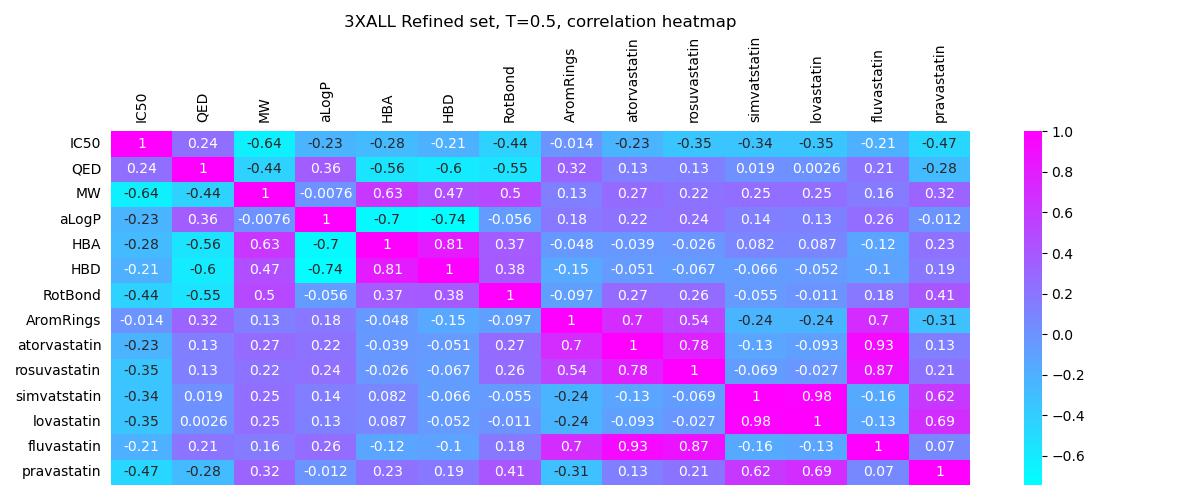

Supplement: Supplementary file 3 — ci4c01309_si_003.zip [file ci4c01309_si_003.zip › Datasets/xfer_Learning_files/3XALL_model_gen_ic50_results/3XALL_Refined_0p5_heatmap.jpg]

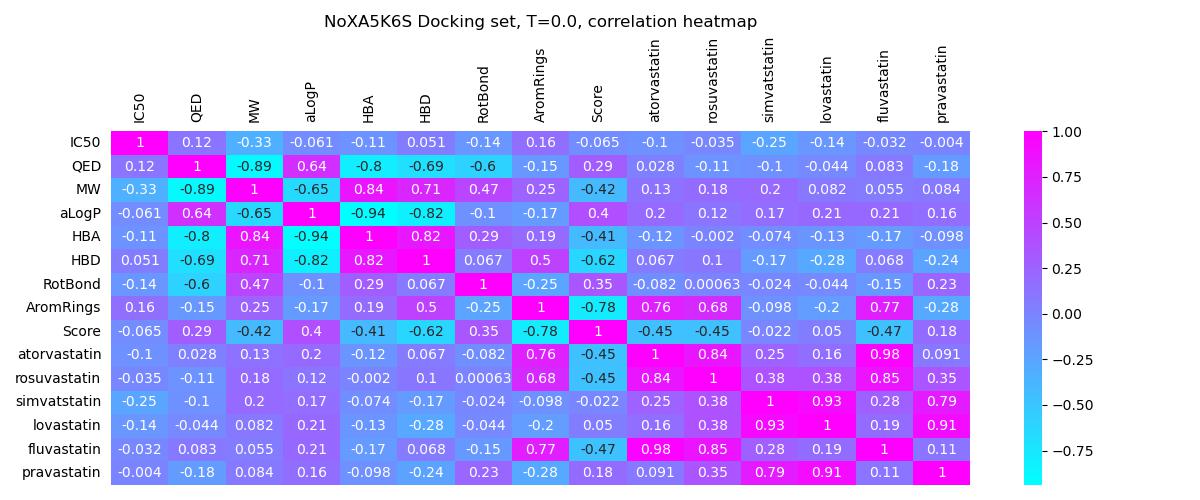

Supplement: Supplementary file 3 — ci4c01309_si_003.zip [file ci4c01309_si_003.zip › Datasets/xfer_Learning_files/NoXA5K6S_model_gen_ic50_results/NoXA5K6S_Docking_0p0_heatmap.jpg]

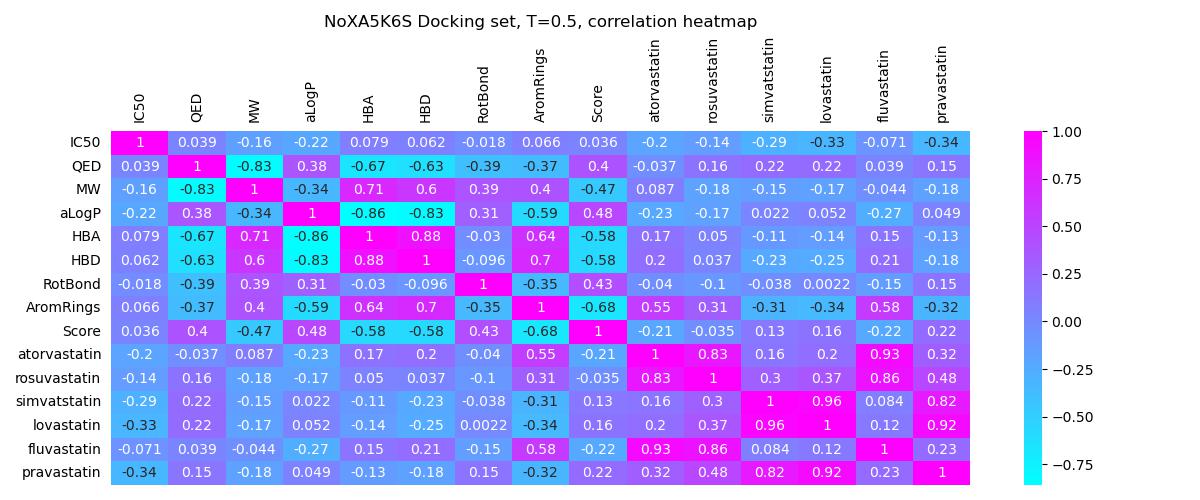

Supplement: Supplementary file 3 — ci4c01309_si_003.zip [file ci4c01309_si_003.zip › Datasets/xfer_Learning_files/NoXA5K6S_model_gen_ic50_results/NoXA5K6S_Docking_0p5_heatmap.jpg]

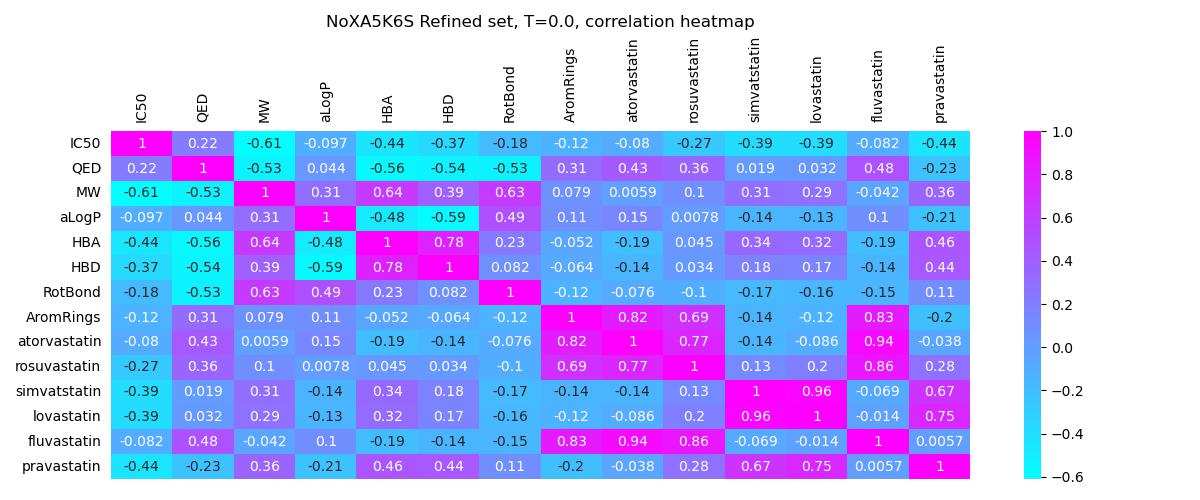

Supplement: Supplementary file 3 — ci4c01309_si_003.zip [file ci4c01309_si_003.zip › Datasets/xfer_Learning_files/NoXA5K6S_model_gen_ic50_results/NoXA5K6S_Refined_0p0_heatmap.jpg]

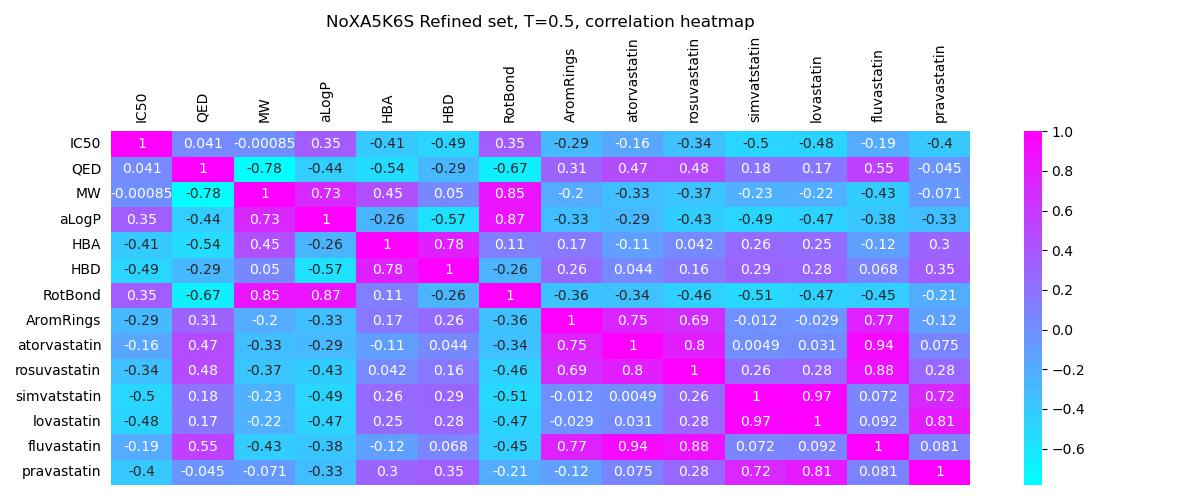

Supplement: Supplementary file 3 — ci4c01309_si_003.zip [file ci4c01309_si_003.zip › Datasets/xfer_Learning_files/NoXA5K6S_model_gen_ic50_results/NoXA5K6S_Refined_0p5_heatmap.jpg]

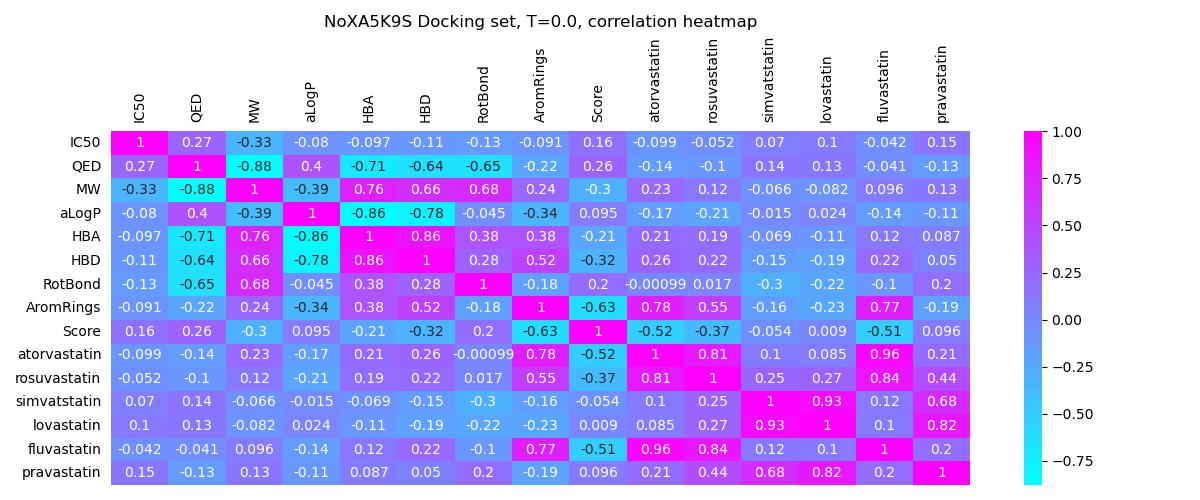

Supplement: Supplementary file 3 — ci4c01309_si_003.zip [file ci4c01309_si_003.zip › Datasets/xfer_Learning_files/NoXA5K9S_model_gen_ic50_results/NoXA5K9S_Docking_0p0_heatmap.jpg]

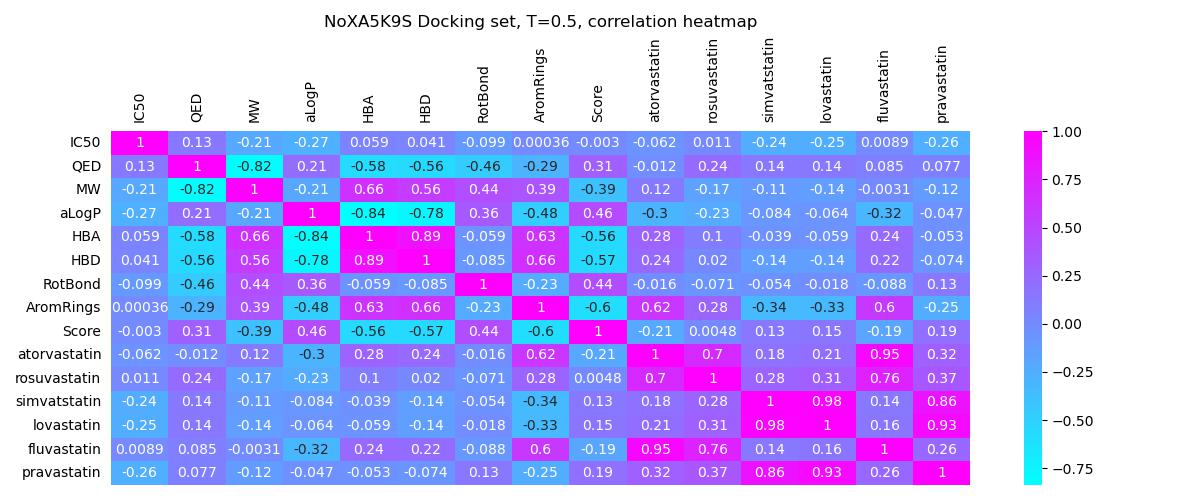

Supplement: Supplementary file 3 — ci4c01309_si_003.zip [file ci4c01309_si_003.zip › Datasets/xfer_Learning_files/NoXA5K9S_model_gen_ic50_results/NoXA5K9S_Docking_0p5_heatmap.jpg]

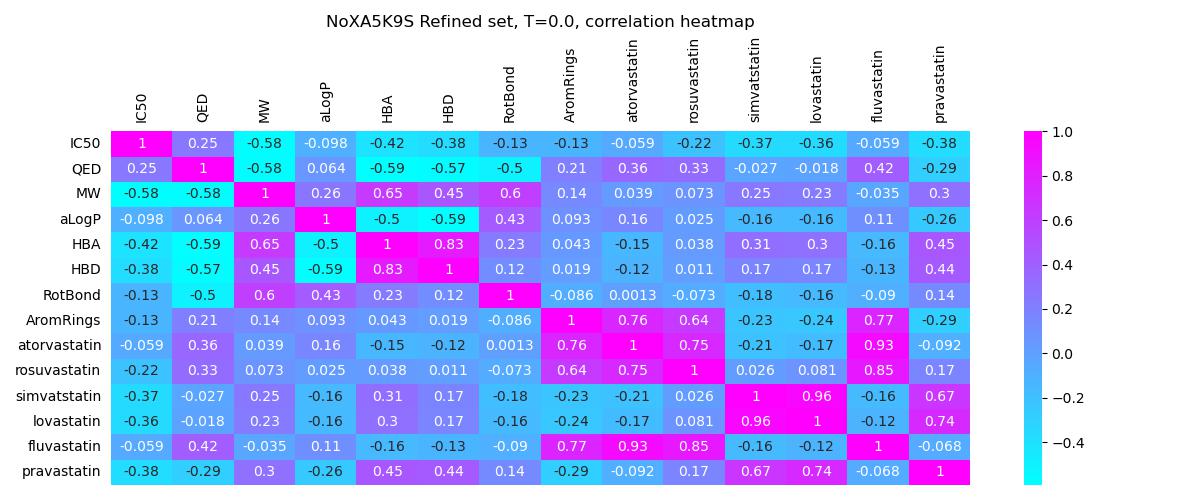

Supplement: Supplementary file 3 — ci4c01309_si_003.zip [file ci4c01309_si_003.zip › Datasets/xfer_Learning_files/NoXA5K9S_model_gen_ic50_results/NoXA5K9S_Refined_0p0_heatmap.jpg]

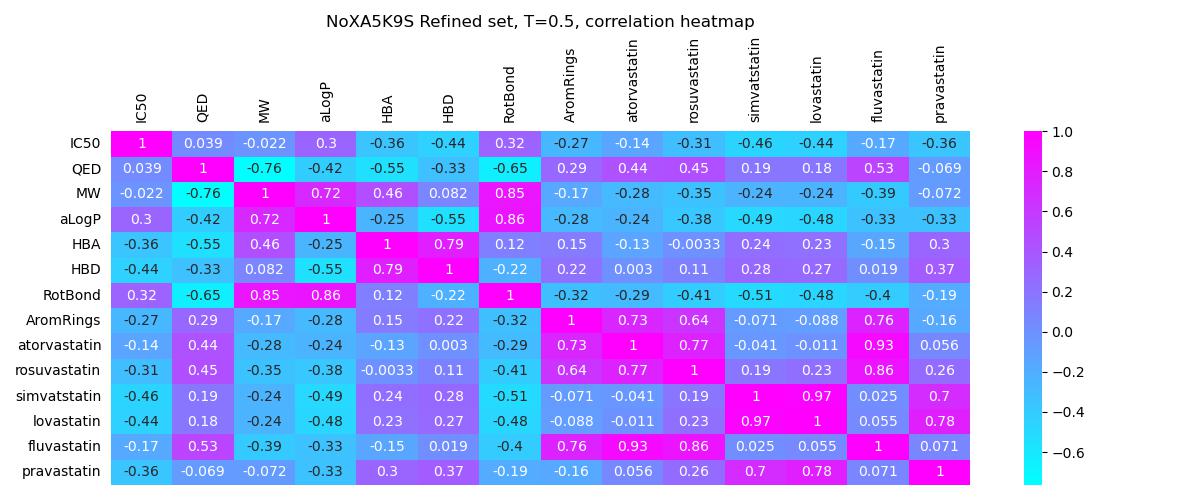

Supplement: Supplementary file 3 — ci4c01309_si_003.zip [file ci4c01309_si_003.zip › Datasets/xfer_Learning_files/NoXA5K9S_model_gen_ic50_results/NoXA5K9S_Refined_0p5_heatmap.jpg]

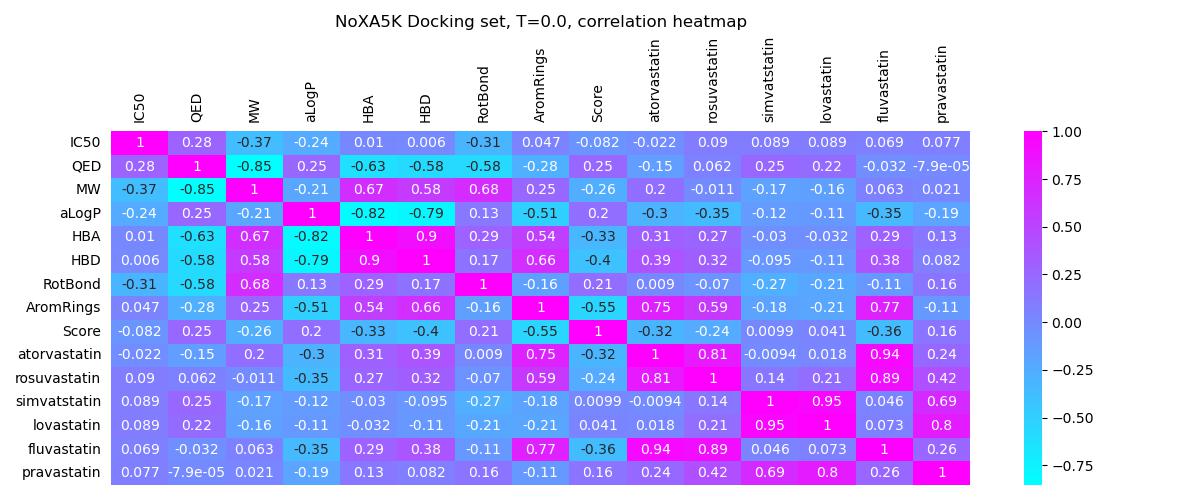

Supplement: Supplementary file 3 — ci4c01309_si_003.zip [file ci4c01309_si_003.zip › Datasets/xfer_Learning_files/NoXA5K_model_gen_ic50_results/NoXA5K_Docking_0p0_heatmap.jpg]

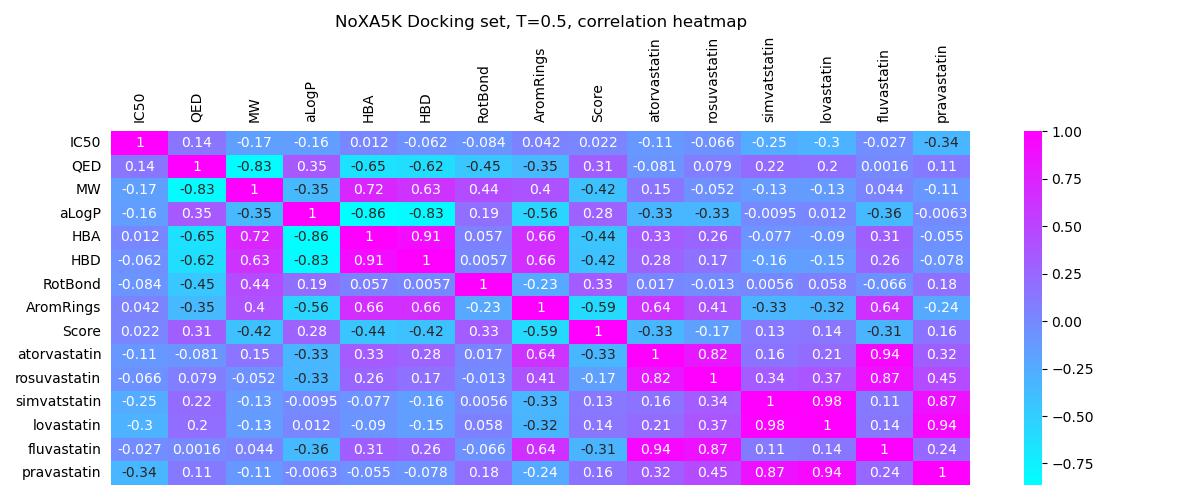

Supplement: Supplementary file 3 — ci4c01309_si_003.zip [file ci4c01309_si_003.zip › Datasets/xfer_Learning_files/NoXA5K_model_gen_ic50_results/NoXA5K_Docking_0p5_heatmap.jpg]

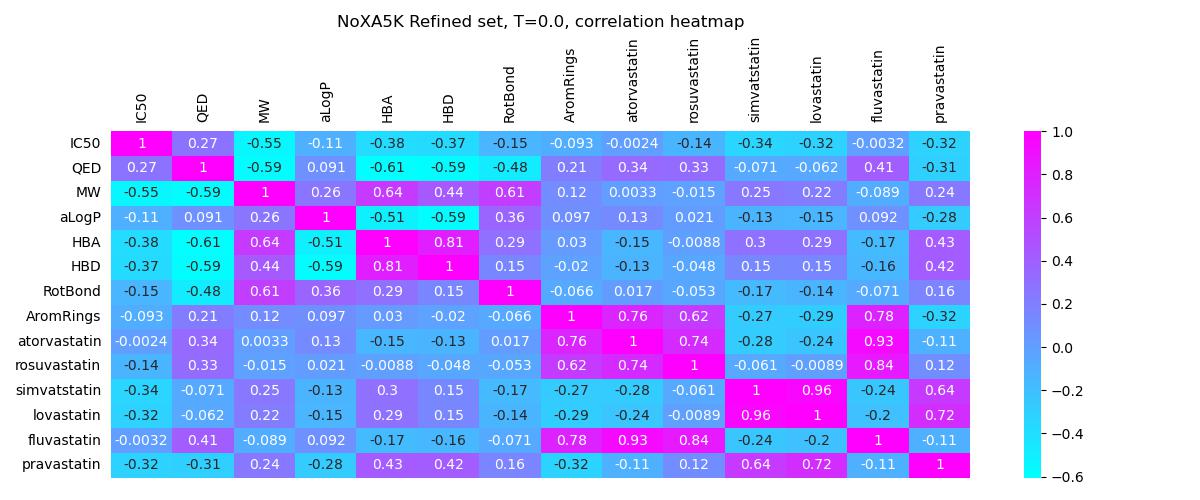

Supplement: Supplementary file 3 — ci4c01309_si_003.zip [file ci4c01309_si_003.zip › Datasets/xfer_Learning_files/NoXA5K_model_gen_ic50_results/NoXA5K_Refined_0p0_heatmap.jpg]

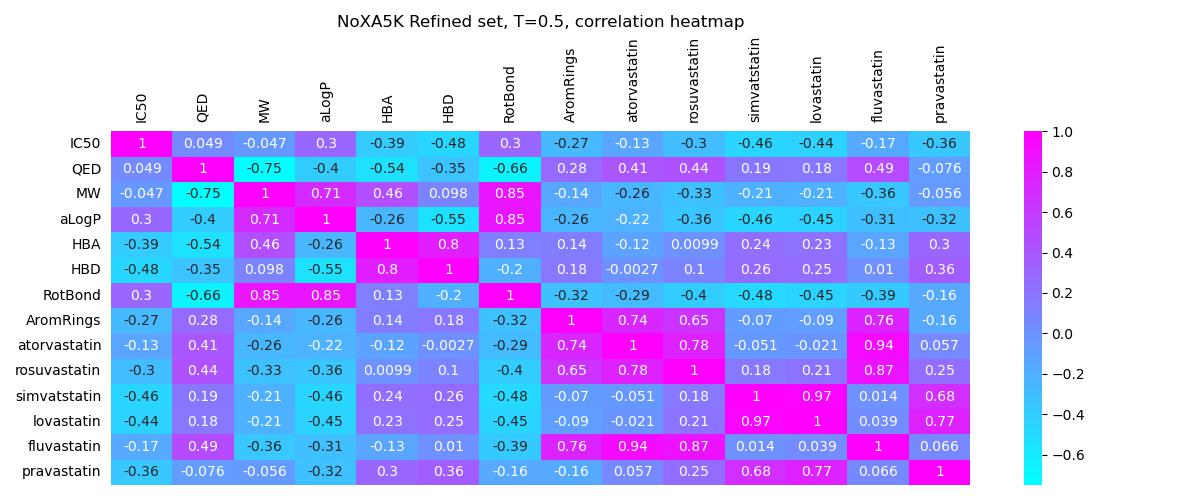

Supplement: Supplementary file 3 — ci4c01309_si_003.zip [file ci4c01309_si_003.zip › Datasets/xfer_Learning_files/NoXA5K_model_gen_ic50_results/NoXA5K_Refined_0p5_heatmap.jpg]
